# Trends in undiagnosed HIV prevalence in England and implications for eliminating HIV transmission by 2030: an evidence synthesis model

**DOI:** 10.1016/S2468-2667(21)00142-0

**Published:** 2021-09-23

**Authors:** Anne M Presanis, Ross J Harris, Peter D Kirwan, Ada Miltz, Sara Croxford, Ellen Heinsbroek, Christopher H Jackson, Hamish Mohammed, Alison E Brown, Valerie C Delpech, O Noel Gill, Daniela De Angelis

**Affiliations:** a Medical Research Council Biostatistics Unit, School of Clinical Medicine, University of Cambridge, Cambridge, UK; b Public Health England, London, UK; c Institute of Global Health, University College London, London, UK

## Abstract

**Background:**

A target to eliminate HIV transmission in England by 2030 was set in early 2019. This study aimed to estimate trends from 2013 to 2019 in HIV prevalence, particularly the number of people living with undiagnosed HIV, by exposure group, ethnicity, gender, age group, and region. These estimates are essential to monitor progress towards elimination.

**Methods:**

A Bayesian synthesis of evidence from multiple surveillance, demographic, and survey datasets relevant to HIV in England was used to estimate trends in the number of people living with HIV, the proportion of people unaware of their HIV infection, and the corresponding prevalence of undiagnosed HIV. All estimates were stratified by exposure group, ethnicity, gender, age group (15–34, 35–44, 45–59, or 60–74 years), region (London, or outside of London) and year (2013–19).

**Findings:**

The total number of people living with HIV aged 15–74 years in England increased from 83 500 (95% credible interval 80 200–89 600) in 2013 to 92 800 (91 000–95 600) in 2019. The proportion diagnosed steadily increased from 86% (80–90%) to 94% (91–95%) during the same time period, corresponding to a halving in the number of undiagnosed infections from 11 600 (8300–17 700) to 5900 (4400–8700) and in undiagnosed prevalence from 0·29 (0·21–0·44) to 0·14 (0·11–0·21) per 1000 population. Similar steep declines were estimated in all subgroups of gay, bisexual, and other men who have sex with men and in most subgroups of Black African heterosexuals. The pace of reduction was less pronounced for heterosexuals in other ethnic groups and people who inject drugs, particularly outside London; however, undiagnosed prevalence in these groups has remained very low.

**Interpretation:**

The UNAIDS target of diagnosing 90% of people living with HIV by 2020 was reached by 2016 in England, with the country on track to achieve the new target of 95% diagnosed by 2025. Reductions in transmission and undiagnosed prevalence have corresponded to large scale-up of testing in key populations and early diagnosis and treatment. Additional and intensified prevention measures are required to eliminate transmission of HIV among the communities that have experienced slower declines than other subgroups, despite having very low prevalences of HIV.

**Funding:**

UK Medical Research Council and Public Health England.

## Introduction

Regular assessment of the burden of HIV is essential to evaluate public health policies aimed at reducing transmission, including treatment as prevention^1^, ^2^, ^3^ and HIV pre-exposure prophylaxis,^4^ and to monitor progress towards elimination of HIV transmission by 2030.^2^ There is no single international consensus definition of elimination; however, a suggested definition for the UK is zero transmission of HIV within the UK by 2030.^5^ Public Health England (PHE) recommends monitoring multiple indicators, including new and late diagnosis, incidence, and undiagnosed prevalence, to assess progress towards the elimination goal. However, incidence and undiagnosed infections are inherently unobservable, so must be estimated.

In the UK, annual estimates of HIV prevalence, both diagnosed and undiagnosed, the number of people living with HIV, and the undiagnosed fraction are published^6^ for: gay, bisexual, and other men who have sex with men (GBM); people who inject drugs (PWID); and heterosexual individuals who in the 2011 UK census self-reported as either Black African or as being from any other ethnic groups.^7^ The latest report^6^ suggests that in 2019, there were an estimated 105 200 (95% credible interval [Crl] 103 300–108 500) people living with HIV in the UK. Of this population, 6600 (4900–9800) or 6% (5–9%) were estimated to be undiagnosed. These estimates are the national indicator for measuring the UK's progress towards the first UNAIDS 90–90–90 by 2020 targets (ie, 90% of all people living with HIV will know their HIV status, 90% of all diagnosed people will receive sustained antiretroviral therapy [ART], and 90% of all ART recipients will have viral suppression), and the first of the updated 95–95–95 by 2025 targets, and for informing HIV testing guidelines^8^ and prevention campaigns.^9^, ^10^ In 2019, the UK estimates of the continuum of care were 94% diagnosed, 98% on treatment, and 97% virally suppressed.^6^ Since 2005, these estimates of HIV prevalence (providing cross-sectional descriptions of the state of the epidemic) have been derived through a multi-parameter evidence synthesis (MPES)—a Bayesian statistical model that combines and triangulates multiple sources of surveillance and survey data.^11^, ^12^ Information on exposure group sizes, the numbers of people diagnosed and in care, and HIV prevalence from prevalence surveys and testing data, are synthesised to estimate the undiagnosed fraction.

**Research in context**

**Evidence before this study**

In the past decade, combination HIV prevention efforts have been intensified. In early 2019, the goal was set by the UK secretary of state for health and social care to eliminate HIV transmission in England by 2030, with an interim target set in 2020 of an 80% reduction in transmission by 2025. It is crucial to understand both the number of undiagnosed HIV infections remaining in each subpopulation and the recent trends in these numbers, to recognise which groups are most at risk, address inequality, focus the testing efforts, and monitor progress, so that no community is left behind in attaining the goal of eliminating transmission. We searched PubMed and Google Scholar on May 19, 2021, using the terms “HIV”, (“undiagnosed prevalence” OR “undiagnosed fraction”), and “England”, for articles published from Jan 1, 2015, to May 19, 2021. We found 93 articles, most of which were either irrelevant or focused on blood-borne viruses or sexually transmitted infections other than HIV, about other countries or diagnosed cohorts, or which reviewed methods but not estimates, or referenced or reviewed other studies of undiagnosed HIV (in particular, the Public Health England annual report on the HIV epidemic in England and the UK). 12 studies estimated undiagnosed HIV prevalence or incidence in specific population subgroups (eg, men who have sex with men) or settings (eg, sexual health clinics) in England. Only the Public Health England annual report provided estimates of undiagnosed prevalence for the whole country, reported by different exposure groups and regions, and synthesised data from multiple sources, including some of those 12 studies in specific contexts, as an annual assessment of the current state of the epidemic. The 12 studies and the Public Health England report suggest that specific exposure groups are disproportionately affected by undiagnosed HIV. However, these studies typically report from a single point in time, rather than provide trends, or trends are reported for specific groups only, so how undiagnosed HIV prevalence across the country and for all subpopulations is changing over time has, to now, been unknown.

**Added value of this study**

To our knowledge, this is the first study to show updated trends in undiagnosed HIV prevalence in England, by exposure group, age group, gender, ethnicity, and region, in the past decade. Our study shows that considerable progress in preventing HIV transmission has been achieved since 2013, with the number and prevalence of undiagnosed HIV infections more than halving in England between 2013 and 2019. However, we have shown that there is substantial variation between different subpopulations in decline in the prevalence of undiagnosed HIV infection.

**Implications of all the available evidence**

To achieve elimination of HIV transmission in England by 2030, it is crucial that the number of people living with undiagnosed HIV continues to be reduced by testing. The challenge of eliminating transmission among heterosexual populations with very low HIV prevalence outside London is compounded by a high refusal rate for HIV testing in those attending sexual health clinics, and not enough testing in those who do not attend sexual health services.

As the epidemic and data sources have changed over time,^6^ the MPES model has evolved structurally since its creation, thereby continuing to make efficient use of the available data. This Article shows the latest extension to our MPES model, integrating sequential cross-sectional estimates to produce trends (with uncertainty) from 2013 to 2019 in the number of undiagnosed HIV infections and HIV prevalence (diagnosed and undiagnosed), by route of probable HIV exposure (exposure group), ethnicity, gender, age, and region, focusing on England.

## Methods

### Study design

For this evidence synthesis study, the adult population of England for 2013–19 was stratified by: exposure group and ethnicity (GBM, including GBM who are PWID; PWID who are not GBM; Black African heterosexuals; heterosexuals in other ethnic groups [which means all ethnicities other than Black African, not the UK census Other ethnic group]); gender (men, women); age (15–34, 35–44, 45–59, 60–74 years); and region (London, outside of London). These groups were defined to be mutually exclusive: individuals belonging to more than one group (eg, GBM and PWID) were classified according to a hierarchy of risk factors for HIV (ie, GBM, PWID, heterosexual). The GBM and heterosexual exposure groups were further subdivided by attendance at a sexual health clinic in the past year in relation to a sexually transmitted infection (clinic attendee, not a clinic attendee). PWID were stratified by recency of injection (current, if in the last year; ex-PWID, if more than a year ago).

### Multi-parameter evidence synthesis

To estimate HIV prevalence in each stratum, data were combined with prior assumptions in a Bayesian model that encodes the relationships between each data source and the quantities to be estimated. Specifically, the MPES approach^11^, ^12^ consists of: defining the key quantities (known as basic parameters) to be estimated, with pre-existing knowledge of these quantities summarised by prior distributions; relating mathematically the information from each data source to the basic parameters, defining the likelihood of the data; and updating the prior distribution with current knowledge, quantified by the likelihood, to obtain a posterior distribution of all parameters, that summarises all uncertainty in both data and parameters. The posterior distribution of any other unknown quantity of interest, which can be expressed as a function of the basic parameters, can also be derived. This method ensures that resulting estimates are consistent with all included data and model assumptions. We used a Markov chain Monte Carlo algorithm to draw samples from the posterior distribution, summarised using their median as a point estimate and their 2·5 and 97·5 percentiles as a 95% CrI. Posterior probabilities of a decrease in each outcome during 2013–19 were also calculated, as the proportion of posterior samples that were smaller in 2019 compared with 2013. These posterior probabilities have a literal interpretation of the probability that a decrease has occurred. We assessed the goodness of fit of the model via deviance summaries and in-sample posterior predictive checks comparing the data with the predictive distribution. All analyses were done using R version 4.0.3, rstan 2.21.2, and Stan 2.21.0.

### Model

We estimated three basic parameters for each stratum *agsrt* defined by age group *a*, exposure group *g*, gender *s*, region *r,* and year *t*: first, ρ_agsrt_ the proportion of the population in stratum *asrt* in exposure group *g*; second, π_agsrt_ HIV prevalence in stratum *agsrt*; and third, δ_agsrt_ the proportion of HIV infections diagnosed in stratum *agsrt*.

Given knowledge of these basic parameters, any related quantity can be estimated as a function of the basic parameters. The key functional parameters are: the number of people living with HIV in each stratum, *N*_asrt_ρ_agsrt_π_agsrt_, where *N*_asrt_ is the total population in stratum *asrt*; the number of undiagnosed infections, *N*_asrt_ρ_agsrt_π_agsrt_ (1 – δ_agsrt_); and the corresponding undiagnosed prevalence, *u*_agsrt_ = π_agsrt_ (1 – δ_agsrt_).

Data availability was uneven across different strata. Estimates of HIV prevalence *π*_agsrt_ and the proportion diagnosed δ_agsrt_ for strata with less data were smoothed by borrowing strength from strata with more data, to increase precision. This smoothing was achieved via a hierarchical random effects model, assuming the log odds ratios (LORs) of prevalence and proportion of people diagnosed in those who did not attend clinics versus clinic attendees might plausibly be similar, but not exactly equal, across strata. Smoothing of trends in the LORs across years was achieved by also linking them via a random walk.

### Data and assumptions

A substantial range of evidence is available in England to inform exposure group sizes and HIV prevalence, either directly or indirectly, as summarised together with key assumptions in table 1. A brief description of the data and their relationships to the parameters follows, with full details given in the appendix (pp 3–28).

**Table 1:** Key data sources and assumptions informing the main parameters of our evidence synthesis, by exposure group

	**Gender**	**Proportion of the population in the exposure group**	**Prevalence of HIV**	**Proportion of people living with HIV whose infection has been diagnosed**	**Prevalence of diagnosed HIV**	**Prevalence of undiagnosed HIV**	**Number of people living with diagnosed HIV**
**GBM**							
Clinic attendee	Men	GUMCAD	NA	NA	GUMCAD; assumed that the mid-year prevalence is between a lower limit of previously diagnosed prevalence and an upper limit of previously diagnosed prevalence plus newly diagnosed prevalence	GUMCAD; assumed that the mid-year prevalence is between a lower limit of undiagnosed HIV prevalence among people either not offered a test or who opted out of testing, and an upper limit of undiagnosed HIV prevalence in that group plus newly diagnosed prevalence; assumed that the proportion of people who would test positive for HIV among those not offered a test is similar to the proportion among people who did test, but with greater uncertainty; assumed that the proportion of people who would test positive for HIV among those who opted out of testing is greater than among those who tested	NA
Not a clinic attendee	Men	Difference between Natsal-3 and GUMCAD (indirect information)	NA	NA	NA	NA	NA
All	Men	Natsal-3; assumed that the proportion of men who are GBM remains constant over time; assumed that the proportion of GBM who are clinic attendees is time-dependent	NA	NA	NA	NA	HARS; expressed as the proportion of the total number of men living with diagnosed HIV who are in the GBM group, in either the clinic attendee or the not a clinic attendee subgroup
**People who inject drugs**							
Current	Men, women	Capture–recapture studies:^13^, ^14^ assumed Hay et al^13^ provides a lower bound for the number of current PWID and that the actual yearly number of current PWID is 1–4 times larger; King et al^14^ for age and gender distributions	UAM	UAM	NA	NA	NA
Ex	Men, women	Sweeting et al^15^ evidence synthesis provided ex:current PWID log odds ratio	NA	Assumed that the proportion diagnosed is larger than for current PWID (indirect information)	NA	NA	NA
All	Men, women	NA	NA	NA	NA	NA	HARS; expressed as a proportion of the total number diagnosed who were in the current-PWID group plus ex-PWID group
**Heterosexuals (Black African)**							
Clinic attendees	Men, women	GUMCAD	NA	NA	GUMCAD; assumed that the mid-year prevalence lies between a lower bound of previously diagnosed prevalence and an upper bound of previously diagnosed prevalence plus newly diagnosed prevalence	GUMCAD; assumed that the mid-year prevalence lies between a lower bound of undiagnosed prevalence among those not offered a test or who opted out of testing, and an upper limit of undiagnosed HIV prevalence in that group plus newly diagnosed prevalence; assumed that the proportion of people who would test positive for HIV among those not offered a test is similar to the proportion among people who did test, but with greater uncertainty; assumed that the proportion of people who would test positive for HIV among those opting out of testing is greater than the proportion positive among those who tested	NA
Not clinic attendees	Men, women	Difference between ONS and GUMCAD data (indirect information)	NA	NA	NA	NA	NA
All	Men, women	ONS 2011 census;^7^ the proportion self-reporting as Black African was applied to the proportion of the population not in the GBM or PWID groups; assumed that the census proportion remains constant over time, whereas the proportion not in the GBM or PWID groups is allowed to vary over time, therefore the overall proportion of people in this group varies over time	NA	African Health and Sex Survey;^16^ the proportion self-reporting ever having had an HIV test indirectly informs the male:female odds ratio of the proportion diagnosed	NA	NA	HARS; expressed as a proportion of the total number of people living with diagnosed HIV who are Black African heterosexuals in both the clinic attendee and the not a clinic attendee subgroups
**Heterosexuals (both Black African and other ethnicity subgroups)**							
All	Women	NA	NA	NA	NA	UK National Study of HIV in Pregnancy and Childhood^17^ and ONS livebirths;^17^ the number diagnosed during current pregnancy (not before), divided by the number of women giving livebirth, informs previously undiagnosed prevalence; data on livebirths were not available by mother's ethnicity, only by mother's region of birth, so a submodel related fertility rates in Black African women to fertility rates in women born in sub-Saharan Africa to infer the number of livebirths by ethnicity	NA
**Heterosexuals (other ethnicities than Black African)**							
Clinic attendees	Men, women	GUMCAD	NA	NA	GUMCAD; mid-year prevalence was assumed to lie between a lower bound of previously diagnosed prevalence and an upper bound of previously diagnosed prevalence plus newly diagnosed prevalence	GUMCAD; assumed that the mid-year prevalence is between a lower limit of undiagnosed HIV prevalence among people either not offered a test or who opted out of testing, and an upper limit of undiagnosed HIV prevalence in that group plus newly diagnosed prevalence; assumed that the proportion of people who would test positive for HIV among those not offered a test is similar to the proportion among people who did test, but with greater uncertainty; assumed that the proportion of people who would test positive for HIV among those who opted out of testing is greater than among those who tested	NA
Not clinic attendees	Men, women	Difference between ONS and GUMCAD data (indirect data)	NA	NA	NA	NA	NA
All	Men, women	ONS 2011 census;^7^ proportion self-reporting an ethnicity other than Black African was applied to the proportion of the population not in a GBM or PWID group; assumed that ONS census proportion was constant over time, whereas the proportion not in the GBM or PWID groups was allowed to vary over time, so that the overall proportion in this group varied over time	National Health Service Blood and Transplant, Public Health England blood donor data;^18^ proportion testing positive among non-GBM blood donors used indirectly to inform the male:female odds ratios of prevalence	NA	NA	NA	HARS; expressed as a proportion of the total number of people living with diagnosed HIV who are heterosexuals in an ethnic group other than Black African, including those in both the clinic attendee and the not a clinic attendee subgroups
**Total**							
All groups	Men, women	ONS mid-year population estimates informed the total population sizes, by gender	NA	NA	NA	NA	HARS; expressed as a sum of the number of people living with diagnosed HIV in each exposure group, in which the sum includes those diagnosed but with an unknown exposure group; by applying the proportions of those with a known exposure route in each group to the total, we inferred the exposure route for those with unknown exposure, assuming exposure was missing at random

GBM=gay, bisexual, and other men who have sex with men. GUMCAD=Genitourinary Medicine Clinic Activity Dataset.^19^ HARS=HIV/AIDS Reporting System.^20^ PWID=people who inject drugs. UAM=Unlinked Anonymous Monitoring.^21^ NA=not available. Natsal-3=third British National Survey of Sexual Attitudes and Lifestyles.^22^ ONS=UK Office for National Statistics.^23^

Annual estimates of the total population of England (by age, gender, and region) were available from the UK Office for National Statistics.^23^ The 2011 UK census^7^ provided information on the proportions of the population by self-reported ethnicity (Black African or any other), which was used to derive the yearly distributions by ethnicity of the heterosexual population. Survey-weighted estimates of the proportion of men who are GBM (by age and region) were available from the third National Survey of Sexual Attitudes and Lifestyles (Natsal-3),^22^ which was a national stratified probability sample survey for Britain from 2010–12**.** Information on PWID (both current and ex) population sizes, based on data from 2005 to 2012,^13^, ^14^, ^15^ was used to estimate relative group sizes for 2013, which were assumed not to change over time. The sizes of the clinic-attending subgroups of GBM and heterosexuals by ethnicity, for each year 2013–19, were available from the Genitourinary Medicine Clinic Activity Dataset,^19^ a disaggregated, pseudonymised data return to PHE submitted by all commissioned sexual health services across England. The appendix (p 8) gives details of the group size parameters, their prior distributions or functional forms, and which datasets informed them.

The sexual health clinic data also provided yearly indirect evidence on HIV prevalence, both diagnosed and undiagnosed, in clinic attendees. Four components of HIV prevalence can be derived from these data combined with model assumptions: previously diagnosed prevalence; newly diagnosed prevalence; prevalence of undiagnosed infection in attendees not offered an HIV test; and prevalence of undiagnosed infection in attendees opting out of an HIV test (appendix p 8). The data directly inform the diagnosed components, whereas the undiagnosed prevalences were estimated by relating them to newly diagnosed prevalence. The Gay Men's Sexual Health Survey^24^ samples GBM at community venues in London every 2–3 years. Participants are offered an HIV test and asked about their sexual health service attendance during the past year and any previous HIV diagnosis. Since participants might be at higher risk of acquiring HIV than GBM in general, this source overestimates HIV prevalence in all GBM. The data were therefore used indirectly, to inform the odds ratio (OR) of (previously) undiagnosed prevalence in clinic-attending versus other GBM (appendix p 9). The Unlinked Anonymous Monitoring annual survey of PWID^21^ recruits attendees at drug services and involves a (self-reported) questionnaire and HIV test. The survey provided information on HIV prevalence, *π*_agsrt_, and the proportion of PWID living with HIV who have ever had an HIV diagnosis, δ_agsrt_, for each year 2013–19. The sampled population was assumed to represent current PWID. For the ex-PWID group, we assumed that the proportion living with HIV whose infection had been diagnosed was larger than for current PWID (appendix p 9).

The UK National Study of HIV in Pregnancy and Childhood^17^ collects data on all HIV diagnoses among pregnant women. Information on diagnoses occurring during pregnancy was combined with data from the UK Office for National Statistics on the annual number of livebirths,^25^ to inform previously undiagnosed prevalence in non-PWID women younger than 45 years (appendix p 10), by ethnicity, age, and region. The African Health and Sex Survey,^16^ done across England in 2014, provided information on the proportion of African people living with HIV who self-reported ever having had an HIV test. We used these data to inform the male-to-female OR of the proportion diagnosed in Black African heterosexuals (appendix p 10). UK National Health Service Blood and Transplant and PHE test blood donors for blood-borne viruses,^18^ with results available by gender, region, and age. The HIV prevalence in this population, which is considered to be at very low risk of HIV infection, informed the male-to-female OR of HIV prevalence in heterosexuals who were not clinic attendees (appendix p 10).

The HIV/AIDS Reporting System^20^ records all new HIV diagnoses, and the clinical status of all patients diagnosed HIV-positive who attend HIV outpatient services for care. These data informed the number of people living with diagnosed HIV, by ethnicity, age, gender, region, and year, and the probable HIV-exposure group distribution of these individuals (appendix p 11). The exposure group recorded does not distinguish between current PWID and ex-PWID, or by clinic attendance. The numbers of people diagnosed were therefore expressed as sums of the numbers in the component subgroups.

### Role of the funding source

The funder of the study had no role in study design, data collection, data analysis, data interpretation, or writing of the report.

## Results

**Figure 1:**
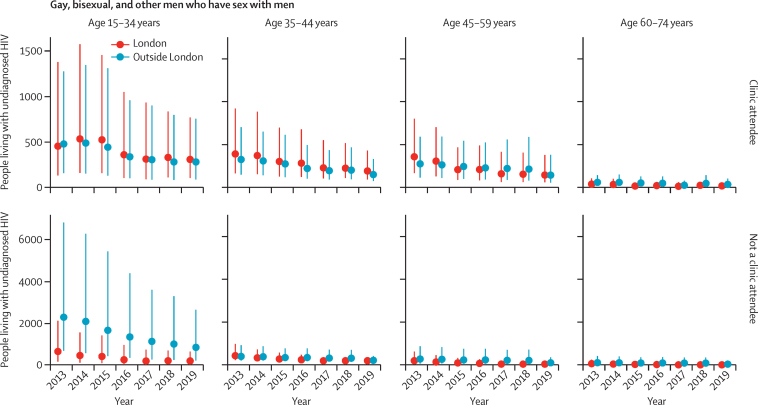
Number of gay, bisexual, and other men who have sex with men in England living with undiagnosed HIV, by clinic attendance, region, age, and year for 2013–19 Note the differing scales of the y-axes by clinic attendance. Dots indicate the posterior median number of people living with undiagnosed HIV. Error bars show the 95% credible interval.

**Figure 2:**
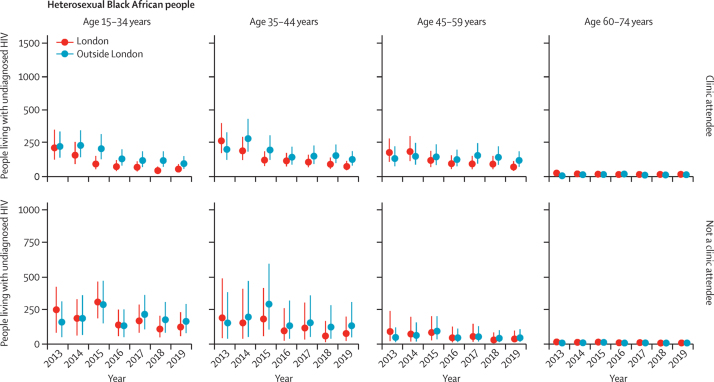
Number of Black African heterosexual people in England living with undiagnosed HIV, by clinic attendance, region, age, and year for 2013–19 Dots indicate the posterior median number of people living with undiagnosed HIV. Error bars show the 95% credible interval.

**Figure 3:**
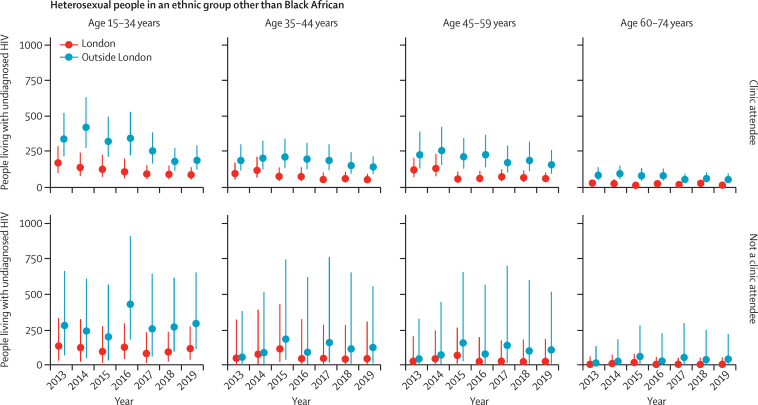
Number of heterosexual people in an ethnic group other than Black African in England living with undiagnosed HIV, by clinic attendance, region, age, and year for 2013–19 Dots indicate the posterior median number of people living with undiagnosed HIV. Error bars show the 95% credible interval.

**Figure 4:**
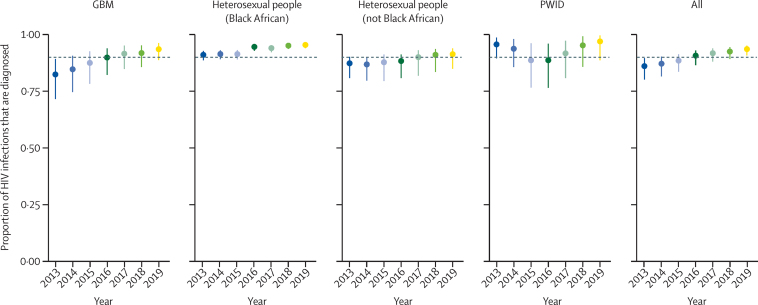
Proportion of people in England living with HIV whose infection is diagnosed, by exposure group and year for 2013–19 Dots indicate the posterior median number of people living with undiagnosed HIV. Error bars show the 95% credible interval. The UNAIDS target of 90% diagnosed is shown by the horizontal dashed black line. Line colours distinguish years. GBM=gay, bisexual, and other men who have sex with men. PWID=people who inject drugs.

In England, the estimated number of people living with HIV aged 15–74 years who were unaware of their infection decreased from 11 600 (95% CrI 8300–17 700) in 2013 to 5900 (4400–8700) in 2019, posterior probability 0·996 (table 2). This decrease corresponded to a halving in undiagnosed HIV prevalence from 0·29 (0·21–0·44) to 0·14 (0·11–0·21) per 1000 population during the study period. The decreases in number of people living with undiagnosed HIV and undiagnosed HIV prevalence were greater in London than outside of London (table 2). Similarly, probabilities of a decrease were higher for clinic attendees than for people who did not attend clinics (Figure 1, Figure 2, Figure 3). An increase in the number of people living with diagnosed HIV resulted in the total number of people living with HIV rising from 83 500 (80 200–89 600) to 92 800 (91 000–95 600) in 2013–19. The percentage of people living with HIV whose infection was diagnosed therefore steadily increased from 86% (80–90%) in 2013 to 94% (91–95%) in 2019, reaching the UNAIDS 90% diagnosed target in 2016, and even earlier, in 2013, for Black African heterosexuals (figure 4). Overall, the burden of HIV, both diagnosed and undiagnosed, was concentrated in the group aged 45–59 years, with the number of people living with HIV within this age group steadily increasing between 2013 and 2019 across all subgroups (appendix p 33).

**Table 2:** Estimated undiagnosed HIV infections in England in 2013 and 2019, by exposure group and region

		**Number of people unaware of their HIV infection**			**Prevalence of undiagnosed HIV per 1000 population**		
		2013	2019	Posterior probability	2013	2019	Posterior probability
Gay, bisexual, and other men who have sex with men*							
London		2600 (1400–5300)	1000 (600–1900)	0·98	18·12 (9·34–35·44)	6·70 (3·55–12·51)	0·99
Outside of London		4300 (2000–9500)	1800 (800–4000)	0·95	11·69 (5·49–25·69)	4·72 (2·18–10·47)	0·95
England		7100 (4000–13 200)	2900 (1600–5300)	0·99	13·86 (7·96–25·33)	5·42 (3·11–9·83)	0·99
**People who inject drugs**†							
London		30 (10–80)	20 (0–80)	0·68	2·33 (0·68–6·34)	1·42 (0·16–5·91)	0·69
Outside of London		40 (10–120	30 (0–140)	0·61	0·47 (0·14–1·26)	0·35 (0·04–1·50)	0·61
England		70 (20–200)	50 (10–220)	0·63	0·71 (0·21–1·86)	0·49 (0·06–2·03)	0·64
Heterosexuals*							
Black African							
	London	1300 (1000–1700)	500 (300–700)	1·00	3·13 (2·36–4·33)	1·13 (0·80–1·67)	1·00
	Outside of London	1000 (700–1300)	700 (500–1000)	0·90	3·39 (2·55–4·65)	2·55 (1·92–3·51)	0·91
	England	2200 (1800–2900)	1200 (900–1600)	1·00	3·25 (2·58–4·22)	1·71 (1·33–2·29)	1·00
Other ethnicities than Black African							
	London	700 (500–1200)	500 (300–900)	0·87	0·12 (0·08–0·21)	0·07 (0·05–0·15)	0·90
	Outside of London	1300 (1000–2200)	1200 (800–2200)	0·64	0·04 (0·03–·07)	0·03 (0·02–0·07)	0·68
	England	2000 (1500–3300)	1600 (1100–3100)	0·76	0·05 (0·04–0·09)	0·04 (0·03–0·08)	0·79
All							
	London	2000 (1600–2700)	1000 (700–1500)	1·00	0·32 (0·25–0·43)	0·15 (0·11–0·22)	1·00
	Outside of London	2300 (1800–3200)	1900 (1500–3000)	0·79	0·07 (0·05–0·10)	0·06 (0·04–0·09)	0·83
	England	4300 (3500–5700)	2900 (2200–4400)	0·96	0·11 (0·09–0·14)	0·07 (0·06–0·11)	0·97
Total*							
	London	4700 (3300–7400)	2100 (1400–3100)	1·00	0·74 (0·52–0·12)	0·31 (0·22–0·46)	1·00
	Outside of London	6700 (4300–12 000)	3800 (2600–6200)	0·96	0·20 (0·13–0·36)	0·11 (0·08–0·18)	0·97
	England	11 600 (8300–17 700)	5900 (4400–8700)	1·00	0·29 (0·21–0·44)	0·14 (0·11–0·21)	1·00

Numbers are posterior median (95% credible interval) and posterior probability of a decrease over the study period.

* Rounded to the nearest 100.

† Rounded to the nearest 10.

A closer look at each subgroup revealed considerable variability in the pace of reduction of undiagnosed infections (table 2; appendix p 30). The decrease in the number of GBM living with undiagnosed HIV was notable in all subgroups (figure 1), with the total more than halving from 7100 (4000–13 200) in 2013 to 2900 (1600–5300) in 2019 (posterior probability 0·986; table 2). The corresponding undiagnosed prevalence dropped from 13·9 (8·0–25·3) to 5·4 (3·1–9·8) per 1000 population, with similar decreases within and outside of London (figure 4). The number of GBM living with undiagnosed HIV was largest, but also most uncertain, among those aged 15–34 years, with probabilities of a decrease lowest in clinic attendees (posterior probability 0·700 in London, 0·767 outside of London). The decrease in undiagnosed prevalence for this age group of clinic attendees was more pronounced (posterior probability 0·809 in London, 0·915 outside of London). The disparity in regional trends was greatest among people aged 60–74 years, with posterior probabilities of a decrease estimated to be 0·856 in London and 0·754 outside of London.

Because of small sample sizes, estimates of the number of PWID living with undiagnosed HIV were uncertain, with the probability of any decrease during 2013–19 lower than in other groups (posterior probability 0·633; table 2). Undiagnosed prevalence among PWID remained seven times higher than that of all heterosexuals in 2019 (table 2).

Progress in reducing the proportion undiagnosed among Black African heterosexuals was more evident than for people of other ethnicity (Figure 2, Figure 3), particularly in London. Overall, the number of Black African heterosexuals unaware of their HIV infection decreased during 2013–19 from 2200 (1800–2900) to 1200 (900–1600), corresponding to almost a halving in undiagnosed prevalence from 3·3 (2·6–4·2) to 1·7 (1·3–2·3) per 1000 population (table 2). The decrease in the number of people aged 45–59 years living with undiagnosed HIV, both those attending clinics (posterior probability 0·615) and not attending clinics (0·523), during the study period, was marginal, whereas undiagnosed prevalence in the clinic-attending group decreased from 38 (22–64) to 20 (12–31) per 1000 population (posterior probability 0·960). This decrease reflects a substantial increase during the same period in the denominator of clinic attendees, from 3600 (3500–3700) to 6000 (5900–6200).

Although the 90% target was achieved by 2017 for most subgroups, including both Black African heterosexuals and heterosexuals in other ethnic groups as a whole, the target was not reached until 2019 for other ethnicity heterosexuals outside of London who were clinic attendees. Furthermore, there was less evidence than for other subgroups of an increase in the percentage diagnosed with HIV for other ethnicity heterosexuals (appendix p 30), as reflected by the uncertainty in the estimates of the number undiagnosed (figure 3). The total number of other ethnicity heterosexuals living with undiagnosed HIV reduced from 2000 (1500–3300) to 1600 (1100–3100; posterior probability 0·760) during 2013–19, with a corresponding drop in undiagnosed prevalence from 0·052 (0·039–0·085) to 0·041 (0·028–0·077) per 1000 population (table 2). However, such a decrease was not discernible in the two older age groups in this subpopulation outside London, particularly in people who were not clinic attendees (appendix pp 36–37). Despite an estimated decline in the number of people living with undiagnosed HIV among clinic-attending heterosexuals of other ethnicity, the undiagnosed prevalence in this group remained much larger than for those of other ethnic groups who did not attend a clinic (appendix pp 36–37).

For all heterosexuals, trends in the number of people living with HIV unaware of their infection were similar for men and women (appendix pp 31–32), although among Black African clinic-attending heterosexuals, consistently higher numbers of women than men remained undiagnosed (appendix p 31).

## Discussion

In England, GBM, PWID, and Black African heterosexuals remain disproportionately affected by HIV, with considerably higher undiagnosed prevalence per population in 2019 than other ethnicity heterosexuals. We have shown that this prevalence has decreased remarkably from 2013 to 2019, with the first 90 of the UNAIDS 90–90–90 targets achieved in 2016 among people aged 15–74 years in England. The estimated number of people living with HIV unaware of their infection halved to 5900 (4400–8700) during the study period, with similar reductions estimated in all GBM and most Black African heterosexual subgroups.

However, there were three less encouraging findings from our analysis. First, undiagnosed prevalence outside London is not decreasing as fast as in London. Second, although sexual health clinics provide free and confidential HIV testing to all clinic attendees, we estimated that among heterosexuals of an ethnicity other than Black African, undiagnosed prevalence in clinic attendees in 2019 was more than 30 times greater than in those who had not attended in the past year. This disparity implies that many opportunities for testing clinic attendees are being missed. Indeed, the latest PHE report on HIV testing^26^ found that of eligible clinic attendees who were not either GBM, Black African, or born in a high-prevalence country, the proportion declining an HIV test had increased during the previous 5 years, to 27% in 2016. Third, despite the small magnitude of undiagnosed prevalence in heterosexuals of other ethnicity than Black African who did not attend a clinic, we estimated low (40%) probability of a decrease between 2013 and 2019, albeit with large uncertainty in the estimates (figure 3).

Our MPES approach integrates the most recently available datasets to provide estimates of the latest trends in the unobserved burden of HIV in England. A key strength of our model is the quantification of temporal changes in different population strata, which is crucial to prioritising policies and monitoring progress towards elimination of HIV transmission.^8^, ^9^, ^10^ Our estimates rely on model assumptions to identify unobservable quantities, for example, relating undiagnosed prevalence to new diagnoses and smoothing constraints to address data sparsity. We judge these assumptions to be plausible, particularly because robustness is ensured by appropriately allowing for uncertainty. For example, in the estimation of prevalence from the sexual health data, we allowed for the dynamic nature of prevalence by assuming that within each year, prevalence lay between the year-start and year-end prevalence. Model assessment via deviance summaries and posterior predictive checks revealed a very good fit of the model to almost all of the data sources used (appendix pp 47–74). We found some slight lack of fit to the prevalence and group size data on PWID groups, and to the HIV diagnosis data for young GBM. However, the small PWID sample sizes and the large GBM sample sizes both result in absolute differences between the point estimate and data that do not have any practical public health meaning, relative to the uncertainty in the estimates.

A consequence of the HIV epidemic and available data sources evolving over time is the continuing adaptation of the MPES model. One outstanding issue is that the population using opiates, including PWID, is thought to be ageing,^27^ so that the age–gender distribution assumed might be outdated. However, given the low and uncertain estimates of absolute numbers of people living with HIV among PWID, our overall estimates are reasonably robust to this ageing. Changes in migration and other population patterns might also have occurred, such that group sizes have changed since the UK Office for National Statistics census and the Natsal-3 survey were done in 2011. Newer data sources are therefore being sought to supplement the evidence base for subsequent years, with accompanying model development to make better and more efficient use of existing and new data sources. This ongoing work includes a new round of the Natsal survey in 2021–22, updating estimates of the PWID population size, incorporation of information from community and online surveys,^28^, ^29^ extending the MPES model to datasets collected by the other UK countries, and extending the model to propagate the uncertainty in the proportion diagnosed to the rest of the continuum of care—ie, the proportions treated and virally suppressed. Finally, a few of the data sources used (African Health and Sex Survey,^16^ Gay Men's Sexual Health Survey,^24^ and the Unlinked Anonymous Monitoring study of PWID^21^) rely on self-report of previous HIV diagnosis, which might be biased, owing to a reluctance to disclose awareness of status. However, we limited the possible effect of such bias by using data from the two community surveys indirectly, via ORs of either the proportion of HIV infections diagnosed or the undiagnosed prevalence in one group compared with another group. Furthermore, the self-reported proportion diagnosed observed in the PWID study is assumed to be a lower bound for the actual proportion diagnosed.

Other approaches have been used to estimate trends in the number of undiagnosed HIV infections in England. First, a back-calculation approach,^30^ using data on diagnoses over time and CD4 cell count close to time of diagnosis, generates estimates of the number of GBM living with undiagnosed HIV that are consistent with ours, providing a complementary understanding of the HIV epidemic in this group. For the MPES approach, incorporating CD4 cell counts at diagnosis to further validate our undiagnosed prevalence estimates would be complicated by having to account for migration to and from the UK (as indeed the back-calculation approach was). Estimates from PHE^6^ show that, in the past decade, the majority of heterosexual migrants who are diagnosed in the UK are likely to have acquired HIV outside the UK, before migration.^31^ Migrants might, or might not, have already been diagnosed and on treatment at the time of migration. Furthermore, differential rates of migration into particular regions might influence the proportion of people living with undiagnosed HIV.

Second, estimates of the number of people living with HIV from transmission modelling,^32^, ^33^ aimed at forecasting the epidemic and the effects of different possible interventions—particularly in the GBM population—are broadly consistent with our estimates. Third, the UNAIDS Estimation and Projection Package^34^ (a mathematical model of transmission and demographic dynamics to derive prevalence and incidence) provides similar estimates of undiagnosed HIV prevalence in key groups (Kirwan PD, unpublished).

Our estimates have important implications for efforts to eliminate HIV transmission in England and the UK, providing crucial context to the 90–90–90 metrics and key estimates of undiagnosed prevalence for monitoring progress in particular subgroups. Diagnosing HIV infections in heterosexuals of other ethnic groups, especially outside of London, is particularly challenging, whether or not they attend a clinic; the large population and low undiagnosed prevalence imply that in general, members of this group might have no particular reason to consider themselves at risk of HIV, so finding and encouraging those who are at risk to attend and test is not simple. Given that undiagnosed prevalence in heterosexuals of other ethnic groups who were clinic attendees during the past year is more than 30 times greater than in those who had not attended during this period (appendix p 37), the priority must be to ensure that all sexual health clinic attendees are offered and encouraged to accept an HIV test, regardless of ethnicity, rather than the 73% that currently do test.^26^ If clinic attendees living with HIV increasingly are diagnosed, and improved partner notification^35^ is used to accelerate reduction of the undiagnosed fraction of the wider population, the prospect of eliminating HIV transmission becomes increasingly likely.

## Data sharing

Relevant data on which this analysis is based are available on request to PHE in accordance with PHE's HIV and STI data sharing policy at https://www.gov.uk/government/publications/hiv-and-sti-data-sharing-policy. All requests for data access will need to specify the planned use of data and will require approval from PHE before data release.

## Declaration of interests

HM was a PHE technical advisor on the UK National Institute for Health and Care Excellence pending guideline on Reducing sexually transmitted infections (GID-NG10142). All other authors declare no competing interests.
