# Supplementary material for: Trends in undiagnosed HIV prevalence in England and implications for eliminating HIV transmission by 2030: an evidence synthesis model
Source: Lancet Public Health. 2021 Sep 23;6(10):e739–51. doi: 10.1016/S2468-2667(21)00142-0 (PMC8481938; doi:10.1016/S2468-2667(21)00142-0)
Supplement: Supplementary appendix [file mmc1.pdf]

THE LANCET
Public Health

Supplementary appendix

This appendix formed part of the original submission and has been peer reviewed. We post it as supplied by the authors.

Supplement to: Presanis AM, Ross J Harris RJ, Kirwan PD, et al. Trends in undiagnosed HIV prevalence in England and implications for eliminating HIV transmission by 2030: an evidence synthesis model. *Lancet Public Health* 2021; **6:** 739–51.

Technical Appendix

Contents

1 Model overview **3**

2 Bayesian evidence synthesis **5**

3 Model details **6**
3.1 GBM 6
3.1.1 Group sizes 6
3.1.2 Undiagnosed prevalence 6
3.1.3 Diagnosed prevalence 12
3.1.4 Prevalence and proportion diagnosed 13
3.2 PWID 13
3.2.1 Group sizes 13
3.2.2 Prevalence 14
3.2.3 Proportion diagnosed 15
3.2.4 Diagnosed and undiagnosed prevalence 16
3.3 SHC-attending groups 16
3.3.1 Group sizes 16
3.3.2 Prevalence 17
3.4 Heterosexual groups, by ethnicity 21
3.4.1 Group sizes 21
3.4.2 Undiagnosed prevalence in women 22
3.4.3 Proportion diagnosed in the Black African population 23
3.4.4 Prevalence in the lowest risk group 24
3.4.5 Prevalences and proportion diagnosed in non-SHC-attending groups 24
3.5 Number diagnosed, all groups 25
3.6 Hierarchical structure, non-PWID groups 26

4 Selected estimates **29**

5 Model assessment **47**
5.1 Selected data and deviance summaries 47
5.2 Selected posterior predictive checks 55

List of Acronyms

AHSS African Health and Sex Survey

CrI credible interval

DAG directed acyclic graph

GBM gay, bisexual and other men who have sex with men

GMSHS Gay Men's Sexual Health Survey

GUM Genito-urinary medicine

GUMCAD Genito-Urinary Medicine Clinic Activity Dataset

HARS HIV/AIDS Reporting System

HIV Human Immuno-deficiency Virus

HO Home Office

NATSAL National Survey of Sexual Attitudes and Lifestyles

NSHPC National Study of HIV in Pregnancy and Childhood

ONS Office for National Statistics

PLWH people living with Human Immuno-deficiency Virus (HIV)

PWID people who inject drugs

SHC sexual health clinic

SHS Sexual Health Service

SSA sub-Saharan Africa

STI sexually transmitted infection

UAM Unlinked Anonymous Monitoring survey in people who inject drugs (PWID)

1 Model overview

We aim to estimate three key parameters for each of a number of strata:

$\boldsymbol{\rho_{agsrt}}$ the proportion of the population in stratum $asrt$ who are in exposure group g;

$\boldsymbol{\pi_{agsrt}}$ the prevalence of HIV in stratum $agsrt$;

$\boldsymbol{\delta_{agsrt}}$ the proportion of HIV infections in stratum $agsrt$ that are diagnosed;

where a indexes age, g indexes exposure group, s indexes gender, r indexes region and t indexes year. The strata considered are:

Age: $a \in$ {15-34, 35-44, 45-59, 60-74};

Group: $g \in$ {SG,NSG,CIDU,PIDU,SA,NSA,SO,NSO}, representing:

> **SG:** sexual health clinic (SHC)-attending gay, bisexual and other men who have sex with men (GBM), i.e. those who have attended in the last year, including those who inject drugs;
>
> **NSG:** non-SHC-attending GBM, i.e. those who have not attended in the last year, including those who inject drugs;
>
> **CIDU:** non-GBM PWID who have injected recently, i.e. in the last year;
>
> **PIDU:** non-GBM PWID who have not injected in the last year;
>
> **SA:** non-GBM non-PWID Black African heterosexuals who have attended a SHC in the last year;
>
> **NSA:** non-GBM non-PWID Black African heterosexuals who have not attended a SHC in the last year;
>
> **SO:** non-GBM non-PWID heterosexuals of other ethnicities who have attended a SHC in the last year;
>
> **NSO:** non-GBM non-PWID heterosexuals of other ethnicities who have not attended a SHC in the last year.

Gender: $s \in$ {M,W} representing Men and Women;

Region: $r \in$ {L,R} representing London and England outside London;

Year: $t \in \{2012, 2013, 2014, 2015, 2016, 2017\}$.

The number of strata considered is therefore $4 \times 8 \times 2 \times 2 \times 6 = 768$, but $4 \times 2 \times 1 \times 2 \times 6 = 96$ of these are "women GBM" who don't exist, and for whom the three parameters $\rho_{agsrt}, \pi_{agsrt}, \delta_{agsrt}$ are fixed to zero. The total number of parameters we want to estimate is therefore $(768 - 96) \times 3 = 2,016$, although the effective number of parameters is smaller, due to borrowing of strength and model assumptions.

For ease of notation/reference, we define different sets of exposure groups in Table 1.

Group name	Description	Group	Members
ALL	All groups	$\mathcal{G}_{\mathrm{A}}$	SG, NSG, CIDU, PIDU, SA, NSA, SO, NSO
M	Men	$\mathcal{G}_{\mathrm{M}}$	SG, NSG, CIDU, PIDU, SA, NSA, SO, NSO
W	Women	$\mathcal{G}_{\mathrm{W}}$	CIDU, PIDU, SA, NSA, SO, NSO
GBM	GBM	$\mathcal{G}_{\mathrm{GBM}}$	SG, NSG
PWID	PWID	$\mathcal{G}_{\mathrm{PWID}}$	CIDU, PIDU
A	Black African heterosexuals	$\mathcal{G}_{\mathrm{A}}$	SA, NSA
O	other ethnicity heterosexuals	$\mathcal{G}_{\mathrm{O}}$	SO, NSO
S	SHC attendees	$\mathcal{G}_{\mathrm{S}}$	SG, SA, SO
NS	non-SHC-attendees	$\mathcal{G}_{\mathrm{NS}}$	NSG, NSA, NSO
Shet	SHC-attending heterosexuals	$\mathcal{G}_{\mathrm{Shet}}$	SA, SO
NShet	non-SHC-attending heterosexuals	$\mathcal{G}_{\mathrm{NShet}}$	NSA, NSO
H	groups in hierarchy	$\mathcal{G}_{\mathrm{H}} = \mathcal{G}_{\mathrm{GBM}} \bigcup \mathcal{G}_{\mathrm{A}} \bigcup \mathcal{G}_{\mathrm{O}}$	GBM, A, O

Table 1: Exposure group definitions.

2 Bayesian evidence synthesis

To estimate diagnosed and undiagnosed HIV prevalence in each stratum, we combine data from all sources with prior assumptions, in a graphical model that encodes the relationships between the data from each source and the quantities to be estimated. This method, known variously as 'Bayesian evidence synthesis', 'multi-parameter evidence synthesis' or 'generalised evidence synthesis' [1–3], ensures that resulting estimates are consistent with all included data and model assumptions, and that uncertainty is correctly propagated from each data source to the final estimates. The method consists of the following steps:

1. Define the key quantities or 'basic parameters' to be estimated.

   - In our case, these are: the proportion of the population in each HIV exposure-by-ethnicity group g, for each age-gender-region-year ($asrt$) stratum, ρ_{agsrt}; the corresponding stratum-specific HIV prevalence, π_{agsrt}; and the corresponding stratum-specific proportion of HIV infections that are diagnosed, δ_{agsrt}.

2. Define the quantity that the data from each source directly informs, as well as how that quantity or 'functional parameter' relates to the basic parameters.

   - For example, if the data $y^{(i)}_{agsrt}$ from source (i) directly informs a parameter $\psi^{(i)}_{agsrt}$ that represents undiagnosed prevalence in the $agsrt$ stratum, then we can express $\psi^{(i)}_{agsrt}$ in terms of the basic parameters via the function
   $$\psi^{(i)}_{agsrt} = \pi_{agsrt}\left(1 - \delta_{agsrt}\right).$$

3. The likelihood of the data from each source given the parameters is then defined.

   - For example, the data $y^{(i)}_{agsrt}$ might be considered a Binomial sample from a denominator $n^{(i)}_{agsrt}$, in which case the likelihood $L(y^{(i)}_{agsrt} \mid n^{(i)}_{agsrt}, \psi^{(i)}_{agsrt}) = L(y^{(i)}_{agsrt} \mid n^{(i)}_{agsrt}, \pi^{(i)}_{agsrt}, \delta^{(i)}_{agsrt})$ can be expressed in terms of the Binomial distribution.

   - The total likelihood is defined as the product over all independent data sources of the likelihood contribution of the data from each source (i):
   $$L\left(\boldsymbol{y} \mid \boldsymbol{\psi}\right) = \prod_{i,agsrt} L\left(y^{(i)}_{agsrt} \mid \boldsymbol{\psi}\right)$$

   where $\boldsymbol{y} = \bigcup_{i,agsrt} y^{(i)}_{agsrt}$ is the collection of all observations.

4. Finally, inference is carried out in a Bayesian framework, encoding any prior knowledge in a joint prior distribution of the basic parameters, $p(\boldsymbol{\psi})$, and updating this prior with our current knowledge summarised by the likelihood, to obtain the posterior distribution,
$$p(\boldsymbol{\psi} \mid \boldsymbol{y}) \propto p(\boldsymbol{\psi}) L\left(\boldsymbol{y} \mid \boldsymbol{\psi}\right),$$
which summarises our updated knowledge, including all uncertainty in the estimates from both the data and parameters. We use Markov chain Monte Carlo, specifically the No U-Turn Sampler/Hamiltonian Monte Carlo algorithms implemented in the Stan platform[4] to simulate posterior samples from this distribution, which are summarised by their median and a 95% credible interval (credible interval (CrI)) defined by the 2.5 and 97.5%-iles. Posterior probabilities of a decrease from 2013 to 2019 in different quantities, e.g. prevalence, are also calculated as the proportion of posterior samples that are smaller in 2019 compared to 2013, denoted $Pr(2019 < 2013)$. Deviance summaries[5] and posterior predictive checks (Section 5) were used for model assessment; and the Deviance Information Criterion (DIC) to compare models during the model development phase, where necessary. All analyses were carried out in R version 4.0.3[6], using the `rstan` package version 2.21.2[7].

3 Model details

A schematic overview of the model and how the data related to the parameters is given in the directed acyclic graph (DAG) in Figure 1.

Model details are summarised in Table 2 (group sizes), Table 3 (prevalence parameters for GBM, SHC-attendees and PWID), Table 4 (prevalence parameters for heterosexuals) and Table 5 (parameters for numbers of people living with HIV (PLWH)). The model details by group are explained in full in the following subsections.

3.1 GBM

3.1.1 Group sizes

The size of the GBM population is informed by sampling-weighted estimates of the proportion of men who are GBM from the National Survey of Sexual Attitudes and Lifestyles (NATSAL) 2011[8]. The sampling-weighted number of GBM by age group and region, $y_{ar}^{(\text{NATSAL})}$, is considered a binomial sample from the sampling-weighted denominator of men in age group a and region r, $n_{ar}^{(\text{NATSAL})}$, with proportion equal to the proportion of men who are GBM in 2013, ρ_{agsrt}, which has a vaguely informative Beta(1,2) prior:

$$\begin{aligned} y_{ar}^{(\text{NATSAL})} &\sim \text{Binomial}\left(n_{ar}^{(\text{NATSAL})};\ \rho_{agsrt}\right), \quad g = \text{GBM}, s = \text{M}, t = 2013, \\ \rho_{agsrt} &\sim \text{Beta}(1,2). \end{aligned}$$

The age groups for which we have NATSAL data are $a \in \{\text{15-34,35-44,45-74}\}$: we don't stratify ages 45-74 into the two core groups 45-59, 60-74, due to small sample sizes in the older age groups. We therefore assume

$$\rho_{\text{60-74},gsrt} = \rho_{\text{45-59},gsrt} = \rho_{\text{45-74},gsrt}, \quad g = \text{GBM}, s = \text{M}, t = 2013.$$

We further assume that the proportion who are GBM does not change over time, i.e. that

$$\rho_{agsrt} = \rho_{agsr,2013}, \quad g = \text{GBM}, s = \text{M}, t = 2014, \ldots, 2019.$$

To identify the proportion of GBM who have attended a SHC in the last year, the number of GBM attending Sexual Health Service (SHS)s observed in the Genito-Urinary Medicine Clinic Activity Dataset (GUMCAD) sexually transmitted infection (STI) surveillance system, a disaggregated, pseudonymised dataset of all attendances at commissioned SHSs in England[9] (section 3.3.1), $y_{agsrt,1}^{(\text{GUMCAD})}$, are modelled as a binomial sample from the estimated yearly total number of GBM in the population, $N_{asrt}\rho_{agsrt},\ g = \text{GBM}, s = \text{M}$:

$$y_{agsrt,1}^{(\text{GUMCAD})} \sim \text{Binomial}\left(N_{asrt}\rho_{agsrt};\ \xi_{agsrt}^{(\text{SHC})}\right), \quad g = \text{GBM}, s = \text{M},$$

where each proportion $\xi_{agsrt}^{(\text{SHC})} \sim \text{Beta}(1,2)$ has a vaguely informative prior distribution.

The proportions of men who are GBM in the SG and NSG groups are then

$$\begin{aligned} \rho_{a,\text{SG},srt} &= \xi_{a,\text{GBM},srt}^{(\text{SHC})}\rho_{a,\text{GBM},srt}, \\ \rho_{a,\text{NSG},srt} &= \left(1 - \xi_{a,\text{GBM},srt}^{(\text{SHC})}\right)\rho_{a,\text{GBM},srt}, \quad s = \text{M}. \end{aligned}$$

3.1.2 Undiagnosed prevalence

Undiagnosed prevalence in the SG group is informed by GUMCAD data, see Section 3.3. Undiagnosed prevalence is not directly observed in the NSG group, but the Gay Men's Sexual Health Survey (GMSHS)[10] gives indirect information. The survey is carried out in locations in London where higher risk GBM may socialise, such as bars and pubs.

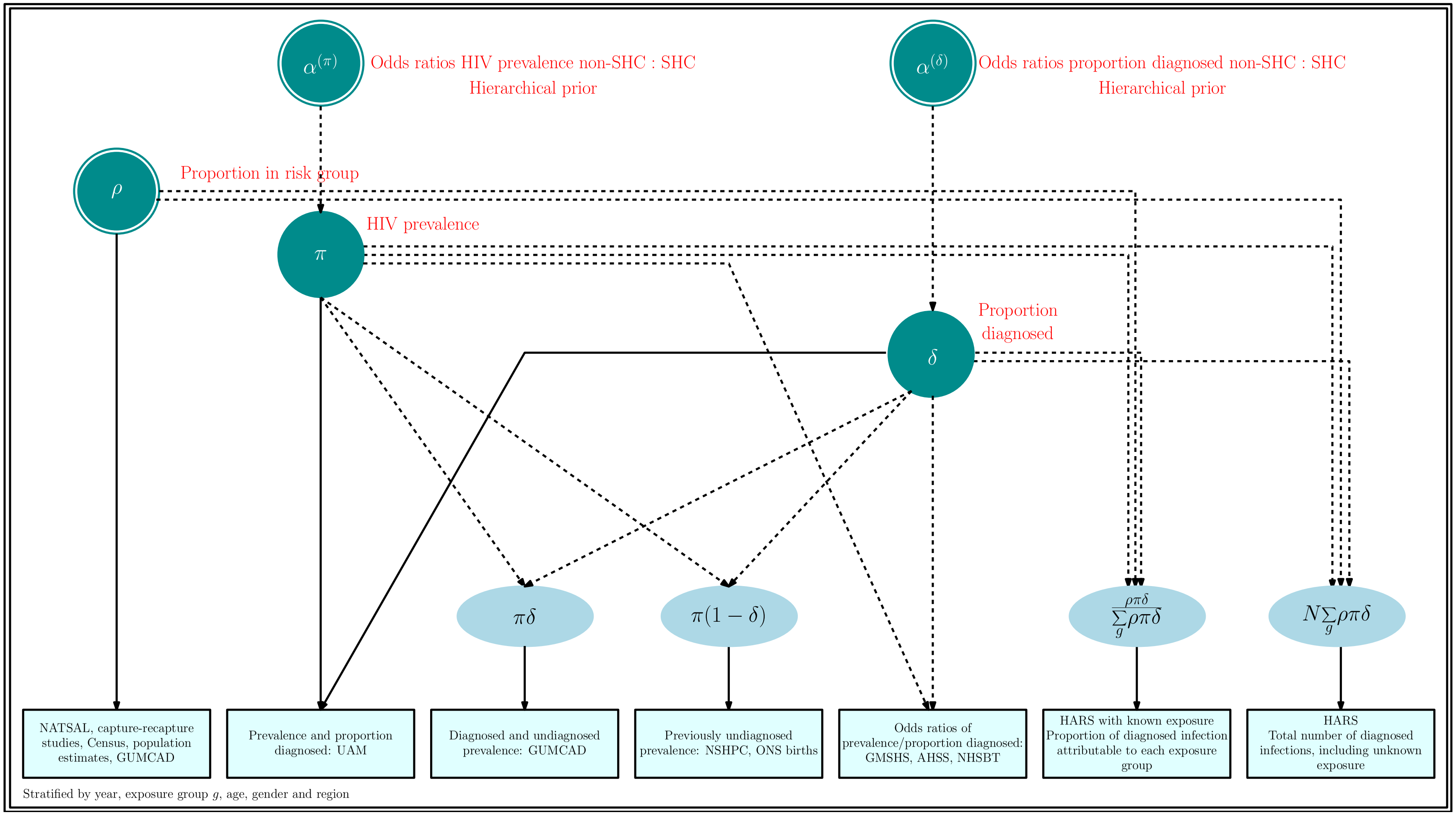

Figure 1: Schematic DAG of the model and how the data relate to the parameters. Circles represent parameters to be estimated, double circles are basic parameters with prior distributions (possibly hierarchical). Rectangles represent data. Solid arrows represent distributional (stochastic) relationships, whereas dashed arrows represent functional (deterministic) relationships. The double rectangle framing the model represents stratification.

Parameter	Description	Prior distribution or functional form	Rationale	Evidence	
				Direct	Indirect
$\rho_{a,\text{GBM},\text{M},r,2012}$	Proportion of men who are GBM in 2013	Beta(1,2)	Vaguely informative prior, mean 33%, median 29%, 95% prior interval (1%, 84%).	NATSAL 2011	
$\rho_{a,\text{GBM},\text{M},r,t}$	Proportion of men who are GBM in 2014-2019	$\rho_{a,\text{GBM},\text{M},rt} = \rho_{a,\text{GBM},\text{M},r,2013}$, $t > 2013$	Assume the same proportion for each year. Given the growth in the total population N_{asrt} over time, the number of GBM, $N_{asrt}\rho_{a,\text{GBM},\text{M},rt}$ also increases over time.		NATSAL 2011
$\xi^{(\text{SHC})}_{agsrt}$	Proportion of group g (GBM, Black African heterosexuals, other ethnicity heterosexuals) who have attended a SHC in the last year	Beta(1,2)	Vaguely informative prior, mean 33%, median 29%, 95% prior interval (1%, 84%).	Numerator: GUMCAD	Denominator: modelled number in group g, $N_{asrt}\rho_{agsrt}$
$\rho_{a,\text{S}gsrt}$	Proportion of all men/women who are in the SHC-attending sub-group of group g (GBM, Black African heterosexuals, other ethnicity heterosexuals).	$\rho_{a,\text{S}gsrt} = \xi^{(\text{SHC})}_{agsrt}\rho_{agsrt}$		As above	
$\lambda_{\text{CIDU}r}$	Lower bound for the number of current PWID (g = CIDU) summed over age and gender for each region r and $t = 2012$, $N_{rt}\,\rho_{grt}$.	Log-linearly regressed on region, regression coefficients given Normal$(0, 5^2)$ priors.	Vague prior, mean 0, median 0, 95% prior interval (-9.8, 9.8).	Home Office & PHE-commisioned capture-recapture estimates, Hay et al (2011).	
$N_{rt}\,\rho_{\text{CIDU}rt}$	Number of current PWID, summed over age and gender for each region r and $t = 2012$.	Sum, on log-scale, of lower bound $\lambda_{\text{CIDU}r}$ and a log-ratio given a log-normal prior expressing that 95% prior mass for the ratio lies between 1 and 4.	Capture-recapture estimates considered to underestimate the actual number of current PWID.		Home Office & PHE-commisioned capture-recapture estimates, Hay et al (2011).
$\xi^{(\text{KING})}_{bsr,b\in\{\text{15-34, 35-64}\}}$	Age-gender breakdown of current PWID - proportion in each of the four age-gender sub-groups.	Dirichlet(1,1,1,1)	Vague prior ensuring the four proportions sum to 1, with mean 25% in each group.	King et al (2014).	
$\rho_{a,\text{CIDU},srt}$	Proportion of all men/women who are current PWID.	$\rho_{a,\text{CIDU},srt} = \begin{cases} \xi^{(\text{KING})}_{\text{15-34},sr} \times N_{..rt}\,\rho_{.\text{CIDU}.rt}/N_{asrt}, & \text{if } a = \text{15-34} \\ 0.5 \times \xi^{(\text{KING})}_{\text{35-64},sr} \times N_{..rt}\,\rho_{.\text{CIDU}.rt}/N_{asrt}, & \text{if } a = \text{35-44} \\ 0.25 \times \xi^{(\text{KING})}_{\text{35-64},sr} \times N_{..rt}\,\rho_{.\text{CIDU}.rt}/N_{asrt}, & \text{otherwise.} \end{cases}$	The King et al (2014) estimates only directly inform two age groups - the first matches the 15-34 age group, but the second is broader, so needs to be distributed across the three age groups 35-44, 45-59, 60-74.		King et al (2014).
$\alpha^{(\rho)}_{\text{15-34,PIDU:CIDU},sr}$	Odds ratio of proportion of population aged 15-34 in 2012 who are ex-PWID relative to corresponding proportion who are current-PWID.	$\text{logit}(\rho_{a,\text{PIDU},srt}) = \text{logit}(\rho_{a,\text{CIDU},srt}) + \log\left(\alpha^{(\rho)}_{a,\text{PIDU:CIDU},sr}\right)$.	Sweeting et al (2009) is an evidence synthesis providing estimates of the ex:current log-odds ratio of group size in the population aged 15-34. The same relationship is assumed for the older age groups, except with the log-odds ratio assumed to increase with age.		Sweeting et al (2009).
$\rho^{(\text{CENSUS})}_{a,\text{A},sr}$	Proportion of the population who report their ethnicity as Black African in 2011, by age, gender and region.	Beta(1,2)	Vaguely informative prior, mean 33%, median 29%, 95% prior interval (1%, 84%).	ONS Census 2011	
ρ_{agsrt}	Proportion of the non-GBM, non-PWID population, by ethnicity.	$\rho_{a,\text{A},srt} = \rho^{(\text{CENSUS})}_{a,\text{A},sr}\left(1 - \sum_{g\in\{\text{GBM,PWID}\}}\rho_{agsrt}\right)$ $\rho_{a,\text{O},srt} = \left(1 - \rho^{(\text{CENSUS})}_{a,\text{A},sr}\right)\left(1 - \sum_{g\in\{\text{GBM,PWID}\}}\rho_{agsrt}\right)$	Assume the same proportion as informed by the Census for each year 2013-2019, applied to the non-GBM, non-PWID population.		ONS Census 2011

Table 2: Model details for exposure group size parameters: their prior distributions or functional forms; and evidence (direct or indirect) contributing to the parameter estimates.

Parameter	Description	Prior distribution or functional form	Rationale	Evidence	
				Direct	Indirect
g_{agsrt1}	Previously diagnosed prevalence among SHC-attending groups.	Beta(1,2)	Vaguely informative prior, mean 33%, median 29%, 95% prior interval (1%, 84%).	GUMCAD	
g_{agsrt2}	Newly diagnosed prevalence among SHC-attending groups.	Normal prior on logit scale.	Based on slight uncertainty around number of new diagnoses in clinic and diagnoses made elsewhere.	GUMCAD	
d_{agsrt}	Diagnosed prevalence among SHC-attending groups.	$d_{agsrt} \in [g_{agsrt1}, g_{agsrt1} + g_{agsrt2}]$	A period prevalence over the whole year is required, so we assume that diagnosed prevalence lies in an interval with lower bound being previously diagnosed prevalence and upper bound additionally including newly diagnosed prevalence.		GUMCAD
g_{agsrt3}	Prevalence of undiagnosed infection due to not being offered a HIV test.	Proportion not offered a test muliplied by positivity among those who accept testing,, but with extra uncertainty.	Assume that those not offered a test have the same prevalence as those who accept a test, but with greater uncertainty expressed via an odds ratio with prior 95% interval (0.5,2).		GUMCAD
g_{agsrt4}	Prevalence of undiagnosed infection due to opting out of HIV testing.	Proportion opting out of testing, multiplied by a positivity that is constrained to be larger than the positivity among those who accept testing.	Assume those who opt out of testing are higher risk than those who opt in. Constraint implemented by a group-specific odds ratio, with 95% prior interval (1,10) for GBM; (1,3) for Black African heterosexuals; and (1,2) for other ethnicity heterosexuals.		GUMCAD
u_{agsrt}	Undiagnosed prevalence among SHC-attending groups.	$u_{agsrt} \in [g_{agsrt3} + g_{agsrt4}, g_{agsrt2} + g_{agsrt3} + g_{agsrt4}]$	Similarly to diagnosed prevalence, a period prevalence over the year t is required, so to account for new diagnoses during the year, we assume undiagnosed lies between a lower bound given by the undiagnosed components, and an upper bound that also includes the newly diagnosed prevalence.		GUMCAD
$\text{logit}\left(u^{(\text{GMSHS})}_{a,\text{NSG},rt}\right) - \text{logit}\left(u^{(\text{GMSHS})}_{a,\text{SG},rt}\right)$	Log-odds ratio of previously undiagnosed prevalence in non-SHC-attending GBM compared to SHC-attending GBM surveyed in GMSHS.	Each undiagnosed prevalence in the two compared groups is assigned a Beta(1,2) prior.	Vaguely informative prior, mean 33%, median 29%, 95% prior interval (1%, 84%).	GMSHS	
$u_{a,\text{NSG,M},rt}$	Undiagnosed prevalence in GBM who have not attended a SHC in the last year.	$\text{logit}(u_{a,\text{NSG,M},rt}) = \text{logit}(u_{a,\text{SG,M},rt}) + \text{logit}\left(u^{(\text{GMSHS})}_{a,\text{NSG},rt}\right) - \text{logit}\left(u^{(\text{GMSHS})}_{a,\text{SG},rt}\right)$.	The GMSHS sample is thought to be higher risk for undiagnosed HIV than the average GBM population, so we use the odds ratio of non-SHC to SHC-attendees rather than absolute prevalences.		GMSHS
$\pi_{a,\text{CIDU},srt}$	Prevalence among current PWID.	Regressed logistically on age, gender, region and year, main effects only. Regression coefficients given $\text{Normal}(0, 5^2)$ priors.	Vague prior, mean 0, median 0, 95% prior interval (-9.8, 9.8).	UAM	
$\delta_{a,\text{CIDU},srt}$	Proportion diagnosed among current PWID.	Regressed logistically on age, gender and year, main effects only. Regression coefficients given $\text{Normal}(0, 5^2)$ priors.	Vague prior, mean 0, median 0, 95% prior interval (-9.8, 9.8). Data on proportion self-reporting a previous HIV diagnosis assumed to represent a lower bound for the proportion diagnosed.	UAM	
$\delta_{a,\text{PIDU},srt}$	Proportion diagnosed among ex-PWID	$\text{logit}\left(\delta_{a,\text{PIDU},srt}\right) = \text{logit}\left(\delta_{a,\text{CIDU},srt}\right) + \log\left(\alpha^{(\delta)}_{a,\text{PIDU:CIDU},srt}\right)$.	Assume the stratum-specific proportion diagnosed is larger than the corresponding current PWID proportion diagnosed, via an odds ratio $\alpha^{(\delta)}_{a,\text{PIDU:CIDU},srt}$ with 95% prior interval (1,13).		UAM and assumption.

Table 3: Model details for prevalence parameters for GBM, SHC-attendees and PWID: their prior distributions or functional forms; and evidence (direct or indirect) contributing to the parameter estimates.

Parameter	Description	Prior distribution or functional form	Rationale	Evidence	
				Direct	Indirect
$u^{(\text{PW})}_{a,\text{A},rt}$	Undiagnosed prevalence in Black African women giving birth.	$u^{(\text{PW})}_{a,\text{A},rt} = \frac{\rho^{(\text{PW})}_{a,\text{SA},rt} u_{a,\text{SA},srt} + \rho^{(\text{PW})}_{a,\text{NSA},rt} u_{a,\text{NSA},srt}}{\rho^{(\text{PW})}_{a,\text{SA},rt} + \rho^{(\text{PW})}_{a,\text{NSA},rt}}$	Assumed to be a weighted average of undiagnosed prevalence in the SHC and non-SHC-attending sub-groups of Black African women, weighted to account for differential fertility by ethnicity compared to the general population of women. The overall undiagnosed prevalence is informed directly by a numerator given by the new HIV diagnoses observed in NSHPC; and indirectly by ONS live birth data by mothers' country of birth. Live births by mothers' ethnicity are inferred from the live births by mothers' country of birth by assuming similar birth rates between Black African women and women born in sub-Saharan Africa (SSA).	Numerator: NSHPC new diagnoses	Denominator: ONS live births
$u^{(\text{PW})}_{a,\text{O},rt}$	Undiagnosed prevalence in Black African women giving birth, age groups 15-34 and 35-44.	$u^{(\text{PW})}_{a,\text{O},rt} = \frac{\rho^{(\text{PW})}_{a,\text{SO},rt} u_{a,\text{SO},srt} + \rho^{(\text{PW})}_{a,\text{NSO},rt} u_{a,\text{NSO},srt}}{\rho^{(\text{PW})}_{a,\text{SO},rt} + \rho^{(\text{PW})}_{a,\text{NSO},rt}}$	Assumed to be a weighted average of undiagnosed prevalence in the SHC and non-SHC-attending sub-groups of women of other ethnicities, weighted to account for differential fertility by ethnicity compared to the general population of women. The overall undiagnosed prevalence is informed directly by a numerator given by the new HIV diagnoses observed in NSHPC; and indirectly by ONS live birth data by mothers' country of birth. Live births by mothers' ethnicity are inferred from the live births by mothers' country of birth by assuming similar birth rates between Black African women and women born in SSA.	Numerator: NSHPC new diagnoses	Denominator: ONS live births
θ_{asrt}	Proportion self-reporting ever testing for HIV, among African respondents to the AHSS.	Beta(2,1) for women, men defined in terms of women and a log-odds ratio: $\text{logit}(\theta_{a,\text{M},rt}) = \text{logit}(\theta_{a,\text{W},rt}) + \log\left(\alpha^{(\text{AHSS})}_{art}\right)$.	Vaguely informative prior, mean 67%, median 71%, 95% prior interval (16%, 99%).	AHSS	
$\alpha^{(\text{AHSS})}_{art}$	Odds ratio of proportion diagnosed in African men compared to African women.	$\log\left(\alpha^{(\text{AHSS})}_{art}\right) = \text{logit}\left(\delta_{ag,\text{M},rt}\right) - \text{logit}\left(\delta_{ag,\text{W},rt}\right), \quad g = \text{A},$	Assume the AHSS-informed odds ratio is a proxy for the male-to-female odds ratio of the proportion of HIV infections diagnosed in the Black African population. The proportions diagnosed are defined as weighted averages of the proportions diagnosed in the SHC- and non-SHC-attending sub-groups.		AHSS
$\pi^{(\text{BD})}_{asrt}$	HIV prevalence in blood donors.	Beta(1,2) for women, men defined in terms of women and a log-odds ratio: $\text{logit}\left(\pi^{(\text{BD})}_{a,\text{M},rt}\right) = \text{logit}\left(\pi^{(\text{BD})}_{a,\text{W},rt}\right) + \log\left(\alpha^{(\text{BD})}_{art}\right)$.	Vaguely informative prior, mean 33%, median 29%, 95% prior interval (1%, 84%).	NHSBT	
$\alpha^{(\text{BD})}_{art}$	Odds ratio of HIV prevalence in men compared to women among non-SHC-attending heterosexual individuals of other ethnicities.	$\log\left(\alpha^{(\text{BD})}_{art}\right)$	$= \text{logit}\left(\pi_{a,\text{NSO},\text{M},rt}\right) - \text{logit}\left(\pi_{a,\text{NSO},\text{W},rt}\right)$.	Assume the NSHBT-informed odds ratio represents the male-to-female odds ratio of HIV prevalence in the lowest risk non-SHC-attending other ethnicity heterosexual group.	
NHSBT					

Table 4: Model details for prevalence parameters for heterosexuals: their prior distributions or functional forms; and evidence (direct or indirect) contributing to the parameter estimates. Heterosexual sub-groups by ethnicity and SHC attendance are denoted by: SA - SHC-attending Black African heterosexuals; NSA - non-SHC-attending Black African heterosexuals; SO - SHC-attending other ethnicity heterosexuals; NSO - non-SHC-attending other ethnicity heterosexuals.

Parameter	Description	Prior distribution or functional form	Rationale	Evidence	
				Direct	Indirect
μ_{asrt}	Total number of men or women living with diagnosed HIV.	$\mu_{asrt} = \sum_{g \in \mathcal{G}_s} \mu_{agsrt}$, where $\mathcal{G}_s$ refers to the Men or Women's subgroups respectively for $s =$ M,W.	The total counts, including those with missing transmission mode information, stratified by age, gender, region and year, are assumed to be the sums over exposure groups of the diagnosed cases.	HARS	
μ_{agsrt}	Exposure group-specific number living with diagnosed HIV.	$\mu_{agsrt} = \nu_{agsrt} N_{asrt} \rho_{agsrt} \pi_{agsrt} \delta_{agsrt}$	Defined in terms of the proportions of the population at risk, group-specific HIV prevalence and proportion diagnosed, adjusted for under-/over-reporting by ν_{agsrt}, which has a prior allowing for up to +/- 3% for all groups except PWID, for whom the adjustment allows up to 30%.		HARS and rest of model
$\xi^{(\text{HARS})}_{agsrt}$	The proportion of diagnosed cases in each exposure group.	$\xi^{(\text{HARS})}_{agsrt} = \frac{\mu_{agsrt}}{\mu_{asrt}}$.	The risk group distribution of diagnosed cases is informed by the observed distribution among cases with non-missing information on transmission mode, using a missing-at-random assumption.	HARS	

Table 5: Model details for parameters for number of people living with diagnosed HIV in each exposure group: their prior distributions or functional forms; and evidence (direct or indirect) contributing to the parameter estimates.

The survey includes a biological sampling component, giving participants an HIV test, and also asks whether participants have a previous HIV diagnosis. A further question determines whether participants have attended a SHC in the last year or not. Data are therefore available from the GMSHS on undiagnosed prevalence in both the SG and NSG groups, but since participants may be higher risk than average GBM, the data are thought to provide over-estimates of HIV prevalence in the whole GBM population. The data are therefore used indirectly, to inform the log-odds ratio of undiagnosed prevalence in NSG compared to SG:

$$y_{agrt}^{(\text{GMSHS})} \sim \text{Binomial}\left(n_{agrt}^{(\text{GMSHS})};\ u_{agrt}^{(\text{GMSHS})}\right), \quad g \in \mathcal{G}_{\text{GBM}}.$$

Due to small sample sizes over time when stratifying by both age group and SHC attendance, the data from the last three surveys, in 2011, 2013 and 2016, have been aggregated, as have the two older age groups 45-59 and 60-74. They are then repeated for each age-region-year combination, assuming the corresponding odds ratios of GMSHS-observed undiagnosed prevalences relative to the actual undiagnosed prevalences are similar across time and region:

$$\begin{aligned}\text{logit}\left(u_{a,\text{NSG},rt}^{(\text{GMSHS})}\right) = {} & \text{logit}\left(u_{a,\text{SG},rt}^{(\text{GMSHS})}\right) + \\ & \text{logit}\left(u_{a,\text{NSG,M},rt}\right) - \text{logit}\left(u_{a,\text{SG,M},rt}\right). \end{aligned} \qquad (1)$$

The GMSHS-observed undiagnosed prevalence in the SHC-attending group, $u_{a,\text{SG}}^{(\text{GMSHS})}$, is given a vaguely informative Beta(1,2) prior, whereas in the non-SHC-attending group, undiagnosed prevalence is already defined functionally in equation (1).

The actual undiagnosed prevalences, u_{agsrt}, are defined for the SG group in section 3.3 (equation (3.3.2)), based on SHC attendance data from GUMCAD. For the NSG group, undiagnosed prevalence in the two younger age groups is defined in terms of the prevalence and proportion diagnosed:

$$u_{a,\text{NSG,M},rt} = \pi_{a,\text{NSG,M},rt}\left(1 - \delta_{a,\text{NSG,M},rt}\right), \qquad a \in \{\text{15-34, 35-44}\}.$$

For the two older age groups, to compensate for less information directly informing these groups, undiagnosed prevalence in the NSG group is subject to age-ordering constraints, by defining $u_{a,\text{NSG,M},rt}$ in terms of the age group $a-1$ and a log-odds ratio given a prior such that undiagnosed prevalence decreases with age:

$$\begin{aligned}&\text{logit}\left(u_{a,\text{NSG,M},rt}\right) = \text{logit}\left(u_{a-1,\text{NSG,M},rt}\right) + \log\left(\alpha_{a,\text{NSG,M},rt}^{(\text{age},u)}\right) \qquad a \in \{\text{45-59, 60-74}\}\\ &\log\left(\alpha_{a,\text{NSG,M},rt}^{(\text{age},u)}\right) \sim \text{Normal}(-0.7, 0.3^2).\end{aligned}$$

The age odds-ratio has prior mean 0.5 and prior standard deviation such that the odds ratio lies approximately in (0.3, 0.9).

3.1.3 Diagnosed prevalence

As for undiagnosed prevalence in the SG group, diagnosed prevalences, d_{agsrt}, are defined in section 3.3 (equation (3.3.2)), based on SHC attendance data from GUMCAD. For the NSG group, diagnosed prevalence in the two younger age groups is defined in terms of the prevalence and proportion diagnosed:

$$d_{a,\text{NSG,M},rt} = \pi_{a,\text{NSG,M},rt}\delta_{a,\text{NSG,M},rt}, \qquad a \in \{\text{15-34, 35-44}\}.$$

For the two older age groups, analogously to the undiagnosed prevalence age-ordering, diagnosed prevalence $d_{a,\text{NSG,M},rt}$ in the NSG group is defined in terms of the age group $a-1$ and a log-odds ratio:

$$\begin{aligned}&\text{logit}\left(d_{a,\text{NSG,M},rt}\right) = \text{logit}\left(d_{a-1,\text{NSG,M},rt}\right) + \log\left(\alpha_{a,\text{NSG,M},rt}^{(\text{age},d)}\right) \qquad a \in \{\text{45-59, 60-74}\}\\ &\log\left(\alpha_{a,\text{NSG,M},rt}^{(\text{age},d)}\right) \sim \text{Normal}(0, 1^2).\end{aligned}$$

However, in contrast to the undiagnosed prevalence age-ordering prior constraint, the age odds-ratio for diagnosed prevalence is given a flatter prior, allowing the age ordering to be determined by the available data on numbers diagnosed (section 3.5) and the rest of the indirect data informing diagnosed prevalence. This flatter prior, on the odds-ratio scale, has mean 1 and standard deviation such that the odds ratio lies approximately in (0.14, 7).

3.1.4 Prevalence and proportion diagnosed

Prevalence π_{agsrt} and the proportion diagnosed δ_{agsrt} in the SG group are defined in terms of the corresponding diagnosed and undiagnosed prevalences:

$$\begin{aligned}\pi_{agsrt} &= d_{agsrt} + u_{agsrt} \\ \delta_{agsrt} &= d_{agsrt}/\pi_{agsrt}, \qquad g = \text{SG}, s = \text{M}.\end{aligned} \tag{2}$$

For the NSG group, to borrow strength across strata, the prevalence $\pi_{a,\text{NSG,M},rt}$ and proportion diagnosed $\delta_{a,\text{NSG,M},rt}$ for the two younger age groups are defined in terms of the hierarchical prior defined in section 3.6. For the two older NSG age groups, prevalence and the proportion diagnosed are defined, as for the SG group in equation (2), in terms of diagnosed and undiagnosed prevalences:

$$\begin{aligned}\pi_{agsrt} &= d_{agsrt} + u_{agsrt} \\ \delta_{agsrt} &= d_{agsrt}/\pi_{agsrt}, \qquad a \in \{\text{45-59, 60-74}\}, g = \text{NSG}, s = \text{M}.\end{aligned}$$

3.2 PWID

3.2.1 Group sizes

The sub-model for PWID group sizes, both current (CIDU) and ex (PIDU) groups, makes use of Home Office (HO) capture-recapture-based estimates of the CIDU population by region from their 2011/2012 release [11]; an age and gender breakdown of the CIDU population from estimates in [12] using 2005/2006 data; and a past:current log odds ratio from estimates in [13]. Since only data from 2005-2012 are available, the group sizes are estimated for one time point, 2013, and are assumed not to change over time.

We assume the HO-based mean estimates, $y_r^{(\text{HO})}$, represent a lower bound, $\lambda_{\text{CIDU},r}$, for the number of current PWID summed over age and gender for each region r and $t = 2013$, $N_{..rt}\ \rho_{.\text{CIDU}.rt}$. The HO estimates are Poisson regressed on region, with a log link:

$$\begin{aligned}y_r^{(\text{HO})} &\sim \text{Poisson}\left(\lambda_{\text{CIDU},r}\right) \\ \log\left(\lambda_{\text{CIDU},r}\right) &= \begin{cases}\beta^{(\rho)}_{\text{CIDU},r}, & \text{if } r = \text{L} \\ \beta^{(\rho)}_{\text{CIDU,L}} + \beta^{(\rho)}_{\text{CIDU,R}}, & \text{otherwise.}\end{cases}\end{aligned}$$

The regression coefficients, $\beta^{(\rho)}_{\text{CIDU},r}$, are given vague Normal$(0, 5^2)$ prior distributions.

The lower bound is enforced by expressing $N_{..rt}\ \rho_{.\text{CIDU}.rt}$, on a log scale, as the sum of the lower bound and a (positive) log-ratio $\text{lr}_{\text{CIDU},r}$:

$$\log\left(N_{..rt}\ \rho_{.\text{CIDU}.rt}\right) = \log\left(\lambda_{\text{CIDU},r}\right) + \text{lr}_{\text{CIDU},r},$$

where $\log\left(\text{lr}_{\text{CIDU},r}\right) \sim \text{Normal}(-2.35, 1.37^2)$ is given a log-normal prior expressing that 95% prior mass for the ratio $\exp(\text{lr}_{\text{CIDU},r})$ lies between 1 and 4.

The age/gender breakdown of the CIDU population from [12] is for two age groups ($b \in \{\text{15-34, 35-64}\}$) by region. The 'observed' numbers (posterior medians from [12]) in each of the four age by gender groups per region, $y_{bsr}^{(\text{KING})}$, are assumed realisations of a multinomial distribution with the corresponding proportions of the total population denoted $\xi_{bsr}^{(\text{KING})}$:

$$\left(y_{bsr}^{(\text{KING})}\right) \sim \text{Multinomial}\left(\xi_{bsr}^{(\text{KING})}; \sum_{bs} y_{bsr}^{(\text{KING})}\right).$$

The proportions $\xi_{bsr}^{(\text{KING})}$ have a vague Dirichlet(1,1,1,1) prior such that they sum to 1.

Out of the total male/female population in each of the modelled age groups $a \in${15-34, 35-44, 45-59, 60-74}, region and year, N_{asrt}, the proportion who are current PWID, $\rho_{a,\text{CIDU},srt}$, is then obtained as the function

$$\rho_{a,\text{CIDU},srt} = \begin{cases} \xi^{\text{(KING)}}_{\text{15-34},sr} \times N_{..rt}\ \rho_{.\text{CIDU}.rt}/N_{asrt}, & \text{if } a = \text{15-34} \\ 0.5 \times \xi^{\text{(KING)}}_{\text{35-64},sr} \times N_{..rt}\ \rho_{.\text{CIDU}.rt}/N_{asrt}, & \text{if } a = \text{35-44} \\ 0.25 \times \xi^{\text{(KING)}}_{\text{35-64},sr} \times N_{..rt}\ \rho_{.\text{CIDU}.rt}/N_{asrt}, & \text{otherwise.} \end{cases}$$

We obtain a past:current log-odds ratio for the 15-34 age group in 2012 by gender and region, $\log\left(\alpha^{(\rho)}_{15-34,\text{PIDU:CIDU},sr}\right)$, from[13]: the 'observations' $y^{\text{(SWEETING)}}_{gsr}$, $g \in \mathcal{G}_{\text{PWID}}$ are posterior estimates that have been converted to pseudo-data numerators/denominators such that the pseudo-data reflect the posterior uncertainty from[13]. They are assumed binomial realisations with proportion parameters $\rho^{\text{(SWEETING)}}_{gsr}$ that have vaguely informative Beta(1,2) priors:

$$\begin{aligned} y^{\text{(SWEETING)}}_{gsr} &\sim \text{Binomial}\left(n^{\text{(SWEETING)}}_{gsr}, \rho^{\text{(SWEETING)}}_{gsr}\right) \\ \rho^{\text{(SWEETING)}}_{gsr} &\sim \text{Beta}(1,2). \end{aligned}$$

The ex:current log-odds ratio is then

$$\log\left(\alpha^{(\rho)}_{15-34,\text{PIDU:CIDU},sr}\right) = \text{logit}\left(\rho^{\text{(SWEETING)}}_{\text{PIDU},sr}\right) - \text{logit}\left(\rho^{\text{(SWEETING)}}_{\text{CIDU},sr}\right).$$

For each of the older age groups 35-44, 45-59, 60-74, we assume the ex:current log-odds ratio increases with age:

$$\log\left(\alpha^{(\rho)}_{a,\text{PIDU:CIDU},sr}\right) = \log\left(\alpha^{(\rho)}_{a-1,\text{PIDU:CIDU},sr}\right) + \text{lr}^{(\rho)}_{a-1,\text{PIDU:CIDU},sr}$$

where the log-ratio $\text{lr}^{(\rho)}_{a-1,\text{PIDU:CIDU},sr}$ is positive. This positivity is ensured by assigning a log-normal prior to the log-ratio such that 95% prior mass lies in $[1.01, 3]$ with mean 1.1:

$$\log(\text{lr}^{(\rho)}_{a,\text{PIDU:CIDU},sr}) \sim \text{Normal}(-2.35, 1.25^2), \qquad a \in \{\text{35-44, 45-59, 60-74}\}.$$

Finally, of the total male/female population in each age group, region and year, N_{asrt}, the proportion who are past PWID, $\rho_{a,\text{PIDU},srt}$, is then obtained as the function

$$\text{logit}(\rho_{a,\text{PIDU},srt}) = \text{logit}(\rho_{a,\text{CIDU},srt}) + \log\left(\alpha^{(\rho)}_{a,\text{PIDU:CIDU},sr}\right).$$

3.2.2 Prevalence

The Unlinked Anonymous Monitoring survey in PWID (UAM)[14] measures HIV prevalence in the PWID population attending needle exchange/methadone treatment centres in England, as well as the proportion of positive participants who were previously aware of their infection (see below). This PWID population is considered to represent only current injectors, so HIV prevalence is observed in the group g = CIDU, and is stratified by age (the three youngest age groups $a \in${15-34, 35-44, 45-59}), gender, region and year. The observed number positive for HIV is assumed to be a realisation from the following Binomial distribution:

$$y^{(\text{UAM},\pi)}_{asrt} \sim \text{Binomial}(n^{(\text{UAM},\pi)}_{asrt}, \pi_{a,\text{CIDU},srt}), \qquad a \in \{\text{15-34, 35-44, 45-59}\}.$$

The CIDU prevalences are regressed logistically on age, gender, region and year, including main effects only:

$$\text{logit}\left(\pi_{a,\text{CIDU},srt}\right) = \beta^{(\pi,\text{int})} + \beta^{(\pi,\text{age})}_a + \beta^{(\pi,\text{gender})}_s + \beta^{(\pi,\text{region})}_r + \beta^{(\pi,\text{year})}_t$$

where the coefficients in the baseline categories ($a = \{15\text{-}44\}, s = \text{M}, r = \text{L}, t = 2013$) are set equal to 0, and each other coefficient has a vague independent Normal$(0, 5^2)$ prior.

For the past injecting group, we assume a vague prior for the prevalence in each age-gender-region-year stratum, $\pi_{a,\text{PIDU},srt} \sim \text{Beta}(1, 2)$.

For the oldest age group, where no prevalence is directly observed, we assume for both the CIDU and PIDU groups that $\pi_{60\text{-}74,gsrt} \leq \pi_{45\text{-}59,gsrt}$ via a log-normal prior on the log-odds ratio of prevalence in the 45-59 group relative to the 60-74 group, constraining the odds ratio to be larger than 1:

$$\begin{aligned}
\text{logit}(\pi_{60\text{-}74,gsrt}) &= \text{logit}(\pi_{45\text{-}59,gsrt}) - \log\left(\alpha_{gsrt}^{\pi,\text{age}}\right), \qquad g \in \mathcal{G}_{\text{PWID}} \\
\log\left(\log\left(\alpha_{gsrt}^{\pi,\text{age}}\right)\right) &\sim \text{Normal}(0, 1^2).
\end{aligned}$$

3.2.3 Proportion diagnosed

The proportion of HIV-positive participants self-reporting ever having a previous diagnosis of HIV in the UAM survey is assumed to represent a lower bound for the proportion of the CIDU group who are diagnosed. Due to small sample sizes, the data are summed over regions, and some prior smoothing is applied by using three-year rolling sums over time to both numerators and denominators. We still stratify by age and gender. A binomial likelihood of the resulting data is again assumed:

$$y_{ast}^{(\text{UAM},\delta)} \sim \text{Binomial}(n_{ast}^{(\text{UAM},\delta)}, \delta_{ast}^{(\text{UAM})}), \qquad a \in \{15\text{-}34, 35\text{-}44, 45\text{-}59\}$$

where the UAM-observed proportion diagnosed is defined in terms of the proportion diagnosed in London:

$$\text{logit}\left(\delta_{ast}^{(\text{UAM})}\right) = \text{logit}(\delta_{a,\text{CIDU},srt}) - \log\left(\alpha_{a,\text{CIDU},srt}^{(\delta)}\right), \qquad r = \text{L}$$

where the lower bound is enforced by assuming the log-odds ratio of the region-specific proportion diagnosed compared to the UAM-measured proportion diagnosed is on the positive real line, with 95% prior mass between 1 and 13 summarised by a log-normal prior:

$$\log\left(\log\left(\alpha_{a,\text{CIDU},srt}^{(\delta)}\right)\right) \sim \text{Normal}(-2, 1.5^2), \qquad r = \text{L}.$$

Outside of London, the log odds ratio is defined in terms of the region-specific proportion diagnosed and the region-independent lower bound:

$$\log\left(\alpha_{a,\text{CIDU},srt}^{(\delta)}\right) = \text{logit}(\delta_{a,\text{CIDU},srt}) - \text{logit}(\delta_{ast}^{(\text{UAM})}), \qquad r = \text{R}.$$

The CIDU proportions diagnosed are regressed logistically on age, gender and year, including main effects only:

$$\text{logit}\left(\delta_{a,\text{CIDU},srt}\right) = \beta^{(\delta,\text{int})} + \beta_a^{(\delta,\text{age})} + \beta_s^{(\delta,\text{gender})} + \beta_t^{(\delta,\text{year})}$$

where the coefficients in the baseline categories ($a = \{15\text{-}34\}, s = \text{M}, t = 2013$) are set equal to 0, and each other coefficient has a vague independent Normal$(0, 5^2)$ prior.

For the PIDU group, we assume their (stratum-specific) proportion diagnosed is larger than the corresponding CIDU group proportion diagnosed, via a log-normal prior implying the odds ratio lies between 1 and 13:

$$\begin{aligned}
\text{logit}\left(\delta_{a,\text{PIDU},srt}\right) &= \text{logit}\left(\delta_{a,\text{CIDU},srt}\right) + \log\left(\alpha_{a,\text{PIDU:CIDU},srt}^{(\delta)}\right) \\
\log\left(\log\left(\alpha_{a,\text{PIDU:CIDU},srt}^{(\delta)}\right)\right) &\sim \text{Normal}(-2, 1.5^2).
\end{aligned}$$

Finally, for the oldest age group, where no proportion diagnosed is directly observed, we assume for both the CIDU and PIDU groups that $\delta_{60\text{-}74,gsrt} \geq \delta_{45\text{-}59,gsrt}$ via a log-normal prior on the log-odds ratio of proportion diagnosed in the 60-74 group relative to the 45-59 group, constraining the odds ratio to be larger than 1:

$$\begin{aligned}
\text{logit}(\delta_{60\text{-}74,gsrt}) &= \text{logit}(\delta_{45\text{-}59,gsrt}) + \log\left(\alpha^{\delta,\text{age}}_{gsrt}\right), \qquad g \in \mathcal{G}_{\text{PWID}} \\
\log\left(\log\left(\alpha^{\delta,\text{age}}_{gsrt}\right)\right) &\sim \text{Normal}(0, 1^2).
\end{aligned}$$

3.2.4 Diagnosed and undiagnosed prevalence

Diagnosed and undiagnosed prevalences in all PWID groups are defined in terms of the corresponding prevalence and proportion diagnosed parameters:

$$\begin{aligned}
d_{agsrt} &= \pi_{agsrt}\delta_{agsrt} \\
u_{agsrt} &= \pi_{agsrt}\left(1 - \delta_{agsrt}\right), \qquad g \in \mathcal{G}_{\text{PWID}}.
\end{aligned}$$

3.3 SHC-attending groups

3.3.1 Group sizes

As specified in sections 3.1.1 and 3.4.1, the sizes of the SHC-attending sub-groups of GBM, Black African heterosexuals and other ethnicity heterosexuals are informed directly by the numbers of these individuals observed in GUMCAD[9] attending sexual health services in the last year. Here we describe our definition of the SHC-attending population in more detail.

Figure 2 displays a tree representation of the count data available in GUMCAD, where each count is defined as follows:

nGUMatt: Number of individuals who have attended a clinic in GUMCAD in year t, of whom:

nContrOnly: have attended only for contraception at all attendances in year t;

nPrevDiag: have previously been diagnosed with HIV at some date *prior* to their first attendance in year t, of whom:

nHIVcareOnly: have attended a clinic in GUMCAD in year t for HIV-related care *only*, at each attendance in year t;

nPDnoHIVcare: have attended a clinic in GUMCAD in year t, *not* for HIV-related care at any attendance in year t.

nPDother: have attended a clinic in GUMCAD for HIV-related care, and *at least once* in year t for any reason (including other SHC testing) other than HIV-related care.

nTestNA: have a code "test not appropriate" and are therefore not eligible for a sexual health screen/HIV test at any of their attendances in year t. This might be because they have tested recently, either in a clinic in GUMCAD or elsewhere;

nEligSHSnotPD: are the remainder who are eligible for a sexual health screen/HIV test at at least one of their attendances in t, of whom:

nOffTest: are offered an HIV test at at least 1 attendance in t, of whom:

nAccTest: accept HIV testing at at least 1 attendance in t, of whom:

nNewDiag: are newly diagnosed with HIV in year t, of whom:

nNDfirst: are newly diagnosed with HIV at their first attendance in year t;

nNDsub: are newly diagnosed at a subsequent attendance in year t, of whom:

nNDsubInClinic: are newly diagnosed in a GUMCAD clinic at a subsequent attendance in year t;

nNDsubOutClinic: are newly diagnosed at another location, then first *present* at the GUMCAD clinic post-diagnosis at a subsequent attendance in year t.

nNegTest: test negative at all attendances where they have accepted HIV testing in year t.

nOptOut: opt out of HIV testing at all attendances where offered in t.

nNotOff: are not offered any HIV test at any attendance in t.

For each exposure group ($\mathcal{G}_{\text{H}} = \{\text{GBM, A, O}\}$), we consider using SHC attendance as a proxy for higher vs lower risk, i.e. to divide each group into two sub-populations at different risk of HIV infection. Ignoring for now the different strata of SHC attendees (by age, gender, region, exposure group and year), we therefore define the SHC-attending population we are interested in, as a proxy for "higher risk for HIV infection", as the number of attendees excluding: those who attend *only* for contraception or HIV care at all their attendances in the year t under consideration; and those with a "Test Not Appropriate" code at each of their attendances in the year t. This definition, in terms of the counts displayed in Figure 2, is:

$$\begin{aligned}\textbf{nGUMattHR} &= \textbf{nGUMatt} - \textbf{nContrOnly} - \textbf{nHIVcareOnly} - \textbf{nTestNA} \\ &= \textbf{nPDother} + \textbf{nPDnoHIVcare} + \textbf{nEligSHSnotPD}.\end{aligned}$$

Those attending for contraception only are not considered to be part of the higher risk population; and those who have been previously diagnosed with HIV and who have attended *only* for their HIV care in a particular year are considered to have moved to a lower risk population (no longer at risk) upon their diagnosis. Those diagnosed who also attend for other reasons, such as SHC testing, are considered still part of the higher risk population, even though they are no longer at risk of HIV, due to their similarity in behaviour to other individuals in the higher risk population.

In each stratum, the observed number attending a SHC, denoted $y^{(\text{GUMCAD})}_{agsrt,1}$ in sections 3.1.1 and 3.4.1, is therefore the stratum-specific count **nGUMattHR**.

3.3.2 Prevalence

We assume HIV prevalence in the SHC-attending population can be decomposed into four components: previously diagnosed prevalence g_{agsrt1}, where "previously" refers to before the current year t; newly diagnosed prevalence g_{agsrt2} in year t; undiagnosed prevalence due to not being offered a test during year t, g_{agsrt3}; and undiagnosed prevalence due to not accepting a test during year t, g_{agsrt4}. Since we are considering a period prevalence over the year t, and clearly the proportions of prevalence in each of the four components will shift over time, even within the year (individuals newly diagnosed in year t are undiagnosed at the start of the year, but diagnosed by the end of the year), some approximations have to be made.

The observed number $y^{(\text{GUMCAD})}_{agsrt,\ell+1}$ at each level $\ell \in \{1, \ldots, 5\}$ (representing the tree levels **nGUMattHR, nEligSHSnotPD, nOffTest, nAccTest, nNewDiagUpp** respectively, with 6 representing **nNewDiagLow** in the notation of

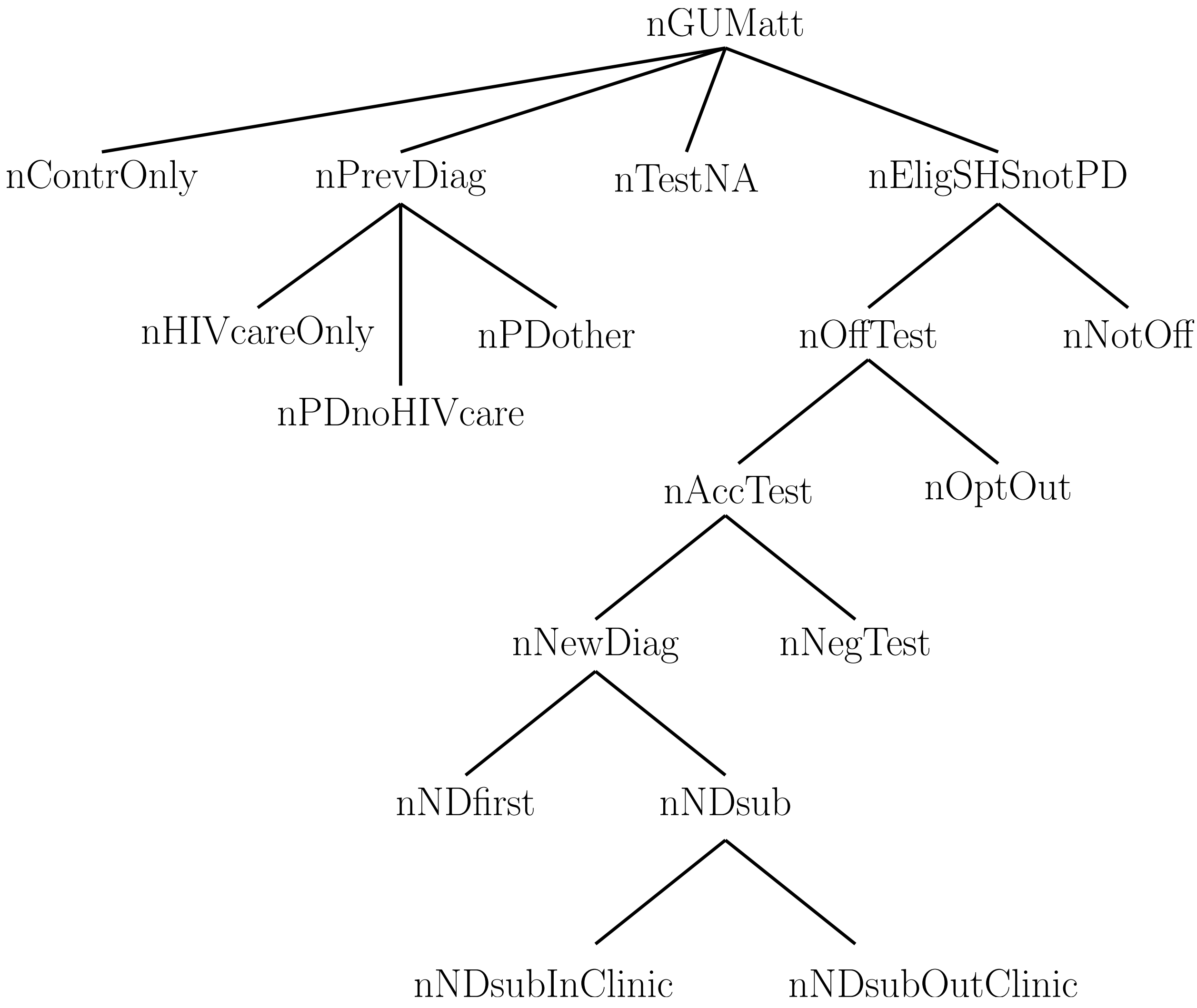

Figure 2: Tree representing GUMCAD data

Figure 2 and section 3.3.1, where **nNewDiagUpp = nNewDiag = nNDfirst + nNDsub** and **nNewDiagLow = nNDfirst + nNDsubInClinic**), is considered binomial with size parameter the number at the previous level, $y^{\text{(GUMCAD)}}_{agsrt,\ell+1}$:

$$y^{\text{(GUMCAD)}}_{agsrt,\ell+1} \sim \text{Binomial}\left(y^{\text{(GUMCAD)}}_{agsrt,\ell}, \gamma_{agsrt\ell}\right)$$

and the resulting nested proportions $\gamma_{agsrt\ell}$ are combined in various ways to obtain the diagnosed and undiagnosed prevalence components. The proportions have vaguely informative Beta prior distributions, as follows:

$$\gamma_{agsrt\ell} \sim \text{Beta}(2,1), \qquad \ell \in \{1,2,3\}$$
$$\gamma_{agsrt\ell} \sim \text{Beta}(1,2), \qquad \ell \in \{4,5\}$$

Figure 3 is a schematic diagram of how the observed counts in the tree of Figure 2 relate to the proportions $\gamma_{agsry\ell}$ and the four components of prevalence $g_{agsrt1}, g_{agsrt2}, g_{agsrt3}$ and g_{agsrt4}.

Previously diagnosed prevalence Previously diagnosed prevalence is defined as

$$g_{agsrt1} = 1 - \gamma_{agsrt,1},$$

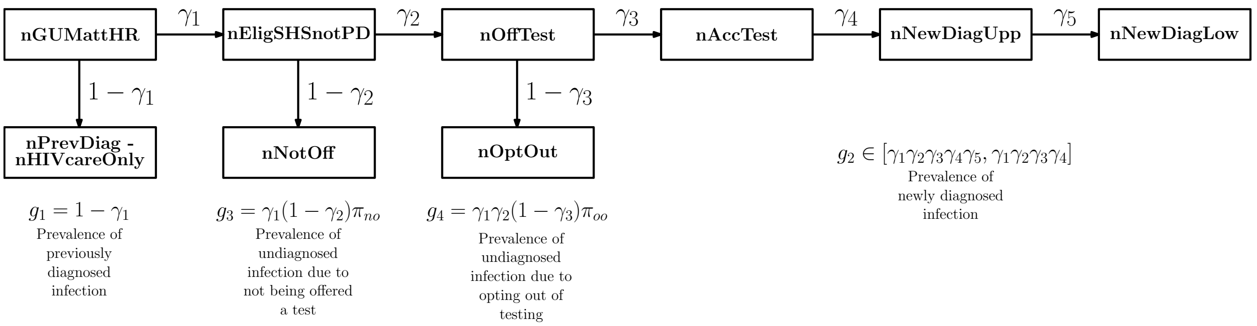

Figure 3: Nested binomial model for HIV prevalence in SHC-attendees: stratum indices have been dropped for clarity.

i.e. the proportion of our defined SHC-attending population, $y^{(\text{GUMCAD})}_{agsrt,1}$, who have a HIV diagnosis prior to their first attendance in year t.

Newly diagnosed prevalence New diagnoses observed in GUMCAD in year t are considered, at a maximum, to be given by the count **nNewDiag**, the total number of diagnoses observed among our defined population of interest in year t, $y^{(\text{GUMCAD})}_{agsrt,1}$. However, an alternative might exclude those diagnosed outside of a SHC, even if they are SHC-attendees, e.g. **nNDfirst + nNDsubInClinic**. Newly diagnosed prevalence is therefore assumed to lie in the following interval:

$$g_{agsrt2} \in \left[\prod_{\ell=1}^{5} \gamma_{agsrt\ell}, \prod_{\ell=1}^{4} \gamma_{agsrt\ell}\right].$$

However, rather than define hard limits, we instead define a Gaussian prior such that the prior mean for newly diagnosed prevalence g_{agsrt2}, on the logit scale, is halfway between the lower and upper bounds, with a prior variance σ^2_{agsrt,g_2} defined such that 95% prior mass on the logit scale lies between the lower and upper bounds:

$$\text{logit}\left(g_{agsrt2}\right) \sim \text{Normal}\left(\text{logit}\left(\frac{\prod_{\ell=1}^{5} \gamma_{agsrt\ell} + \prod_{\ell=1}^{4} \gamma_{agsrt\ell}}{2}\right), \sigma^2_{agsrt,g_2}\right).$$

Total diagnosed prevalence Similarly, since diagnosed prevalence in the SHC-attending population, d_{agsrt}, $g \in \mathcal{G}_{\text{G}}$, effectively changes over time throughout a year under consideration, but we want to consider a period prevalence over the whole year, we assume that diagnosed prevalence lies in an interval with lower bound being previously diagnosed prevalence g_{agsrt1} and an upper bound including newly diagnosed prevalence, i.e. $g_{agsrt1}+g_{agsrt2}$. Rather than define hard limits, we again define a Gaussian prior with prior variance $\sigma^2_{agsrt,d}$ such that 95% of prior mass lies between these bounds:

$$\text{logit}\left(d_{agsrt}\right) \sim \text{Normal}\left(\text{logit}\left(\frac{g_{agsrt1} + g_{agsrt1} + g_{agsrt2}}{2}\right), \sigma^2_{agsrt,d}\right).$$

'Not offered' component of undiagnosed prevalence Undiagnosed prevalence due to not being offered a test is estimated as the proportion $\gamma_{agsrt,1}(1-\gamma_{agsrt,2})$, out of the observed population $y^{(\text{GUMCAD})}_{agsrt,1}$ of interest, that are not offered a test, multiplied by the proportion HIV-positive $\pi^{(\text{NO})}_{agsrt}$ among these individuals:

$$g_{agsrt3} = \gamma_{agsrt,1}(1-\gamma_{agsrt,2})\pi^{(\text{NO})}_{agsrt}$$

where the relevant proportion HIV-positive is

$$\pi^{(\text{no})}_{agsrt} = \text{expit}\left(\text{logit}\left(\pi^{(\text{test})}_{agsrt}\right) + \log\left(\alpha^{(\text{no})}_{agsrt}\right)\right),$$

related to the proportion HIV-positive among those who accept testing, $\pi^{\text{(test)}}_{agsrt}$, but with extra uncertainty given by a Gaussian prior for the log-odds ratio $\log(\alpha^{\text{(no)}}_{agsrt})$, which specifies the odds ratio $\alpha^{\text{(no)}}_{agsrt}$ lying approximately in the interval $(0.5, 2)$ with mean 1.

The positivity among those tested is assumed to lie between the lower and upper bounds of $\gamma_{ary,4}\gamma_{ary,5}$ and $\gamma_{ary,4}$, representing again the uncertainty around the number of new diagnoses observed in GUMCAD, in the observed interval

[**nNDfirst + nNDsubInClinic**, **nNewDiag**].

To achieve this uncertainty, we assume a Gaussian prior on the logit scale for $\pi^{\text{(test)}}_{agsrt}$:

$$\text{logit}(\pi^{\text{(test)}}_{agsrt}) \sim \text{Normal}\left(\text{logit}\left(\gamma_{ary,4}(1+\gamma_{ary,5})/2\right), \sigma^{2\text{(test)}}_{agsrt}\right)$$

'Opt-out' component of undiagnosed prevalence Undiagnosed prevalence due to declining an offered HIV test is estimated as the proportion $\gamma_{agsrt,1}\gamma_{agsrt,2}(1-\gamma_{agsrt,3})$, out of the observed population $y^{\text{(gumcad)}}_{agsrt,1}$ of interest, that are offered a test but decline it, multiplied by the proportion HIV-positive $\pi^{\text{(oo)}}_{agsrt}$ among these individuals:

$$g_{agsrt4} = \gamma_{agsrt,1}\gamma_{agsrt,2}(1-\gamma_{agsrt,3}) \times \pi^{\text{(oo)}}_{agsrt}$$

The HIV-positive proportion among those opting out of testing is defined to be larger than the corresponding proportion positive in those who accept testing, $\pi^{\text{(test)}}_{agsrt}$, by an odds ratio $\alpha^{\text{(oo)}}_{agsrt}$:

$$\pi^{\text{(oo)}}_{agsrt} = \text{expit}\left(\text{logit}\left(\pi^{\text{(test)}}_{agsrt}\right) + \log\left(\alpha^{\text{(oo)}}_{agsrt}\right)\right)$$

The Gaussian prior for the log-odds ratio $\log(\alpha^{\text{(oo)}}_{agsrt})$ is group-dependent: for GBM, the prior mean is $\log(3.16)$ with 95% prior mass in $(\log(1), \log(10))$; for Black African heterosexuals, the prior mean is $\log(1.70)$ with 95% prior mass in $(\log(1), \log(3))$; and for other ethnicity heterosexuals, the prior mean is $\log(1.41)$ with 95% prior mass in $(\log(1), \log(2))$.

Total undiagnosed prevalence Symmetrically to total diagnosed prevalence, we consider a period prevalence over the whole year t, so that effectively we assume undiagnosed prevalence lies in an interval where the lower bound is the sum of the 'not offered' and 'opt-out' contributions to undiagnosed prevalence ($g_{agsrt3} + g_{agsrt4}$), i.e. does not include newly diagnosed prevalence; and an upper bound which does include newly diagnosed prevalence ($g_{agsrt2} + g_{agsrt3} + g_{agsrt4}$). These bounds are implemented as soft bounds via a Gaussian prior with mean half-way between the bounds and prior variance $\sigma^2_{agsrt,u}$ such that 95% prior mass lies within the bounds:

$$\text{logit}\left(u_{agsrt}\right) \sim \text{Normal}\left(\text{logit}\left(\frac{g_{agsrt2} + 2(g_{agsrt3} + g_{agsrt4})}{2}\right), \sigma^2_{agsrt,u}\right).$$

Prevalence and proportion diagnosed The HIV prevalence, π_{agsrt}, and proportion of infections diagnosed, δ_{agsrt}, in the SHC-attending groups are defined directly in terms of the parameters d_{agsrt} and u_{agsrt} representing diagnosed and undiagnosed prevalence respectively, as in equation (2) for the SG group in section 3.1.4:

$$\pi_{agsrt} = d_{agsrt} + u_{agsrt}$$
$$\delta_{agsrt} = \frac{d_{agsrt}}{d_{agsrt} + u_{agsrt}}.$$

3.4 Heterosexual groups, by ethnicity

3.4.1 Group sizes

Information on the proportion of the total population who report their ethnicity as Black African is obtained from the Office for National Statistics (ONS) Population Census from 2011 [15], stratified by age, gender and region. The observed number of Black African individuals, $y^{(\text{CENSUS})}_{a,\text{A},sr}$, is a realisation of Binomial distribution with size the total number of individuals observed, $n^{(\text{CENSUS})}_{asr}$:

$$y^{(\text{CENSUS})}_{a,\text{A},sr} \sim \text{Binomial}\left(n^{(\text{CENSUS})}_{asr}; \rho^{(\text{CENSUS})}_{a,\text{A},sr}\right),$$

and is assumed to inform the proportion $\rho^{(\text{CENSUS})}_{a,\text{A},sr}$ of the population in the Black African heterosexual group in 2012. This proportion is assigned a vaguely informative Beta(1,2) prior.

To retain non-overlapping risk groups, we assume that the proportions of non-GBM, non-PWID individuals in each group (Black African, other ethnicities) are equal to the proportions of all individuals in these groups, as measured by the 2011 Census, in each year t:

$$\begin{aligned}
\rho_{a,\text{A},srt} &= \rho^{(\text{CENSUS})}_{a,\text{A},sr}\left(1 - \sum_{g\in\{\text{GBM,PWID}\}} \rho_{agsrt}\right) \\
\rho_{a,\text{O},srt} &= \left(1 - \rho^{(\text{CENSUS})}_{a,\text{A},sr}\right)\left(1 - \sum_{g\in\{\text{GBM,PWID}\}} \rho_{agsrt}\right)
\end{aligned} \tag{3}$$

As in the GBM group, to identify the subgroups of heterosexuals who have attended a SHC in the last year, the GUMCAD dataset (Section 3.3) is used. The number of heterosexuals attending sexual health services in year t, $y^{(\text{GUMCAD})}_{agsrt,1}, g \in \{\text{A,O}\}$ is a realisation of a Binomial distribution with size the estimated number of heterosexuals in the population, $N_{asrt}\rho_{agsrt}, g \in \{\text{A,O}\}$:

$$y^{(\text{GUMCAD})}_{agsrt,1} \sim \text{Binomial}\left(N_{asrt}\rho_{agsrt};\ \xi^{(\text{SHC})}_{agsrt}\right), \quad g \in \{\text{A,O}\},$$

where each proportion $\xi^{(\text{SHC})}_{agsrt} \sim \text{Beta}(1,2)$ has a vaguely informative prior distribution. The proportions of the population in each of the SHC and non-SHC-attending heterosexual groups are then defined as

$$\begin{aligned}
\rho_{a,\text{SA},srt} &= \xi^{(\text{SHC})}_{a,\text{A},srt}\rho_{a,\text{A},srt} \\
&= \xi^{(\text{SHC})}_{a,\text{A},srt}\rho^{(\text{CENSUS})}_{a,\text{A},sr}\left(1 - \sum_{g\in\{\text{GBM,PWID}\}} \rho_{agsrt}\right) \\
\rho_{a,\text{NSA},srt} &= \left(1 - \xi^{(\text{SHC})}_{a,\text{A},srt}\right)\rho_{a,\text{A},srt} \\
&= \left(1 - \xi^{(\text{SHC})}_{a,\text{A},srt}\right)\rho^{(\text{CENSUS})}_{a,\text{A},sr}\left(1 - \sum_{g\in\{\text{GBM,PWID}\}} \rho_{agsrt}\right) \\
\rho_{a,\text{SO},srt} &= \xi^{(\text{SHC})}_{a,\text{O},srt}\rho_{a,\text{O},srt} \\
&= \xi^{(\text{SHC})}_{a,\text{O},srt}\left(1 - \rho^{(\text{CENSUS})}_{a,\text{A},sr}\right)\left(1 - \sum_{g\in\{\text{GBM,PWID}\}} \rho_{agsrt}\right) \\
\rho_{a,\text{NSO},srt} &= \left(1 - \xi^{(\text{SHC})}_{a,\text{O},srt}\right)\rho_{a,\text{O},srt} \\
&= \left(1 - \xi^{(\text{SHC})}_{a,\text{O},srt}\right)\left(1 - \rho^{(\text{CENSUS})}_{a,\text{A},sr}\right)\left(1 - \sum_{g\in\{\text{GBM,PWID}\}} \rho_{agsrt}\right).
\end{aligned}$$

Since these proportions can be defined directly in terms of $\rho^{(\text{CENSUS})}_{a,\text{A},srt}$, rather than define the quantities in equation (3) in terms of $\rho^{(\text{CENSUS})}_{a,\text{A},srt}$, they can be defined in terms of sums of the SHC and non-SHC-attending groups:

$$\rho_{a,\text{A},srt} = \rho_{a,\text{SA},srt} + \rho_{a,\text{NSA},srt}$$
$$\rho_{a,\text{O},srt} = \rho_{a,\text{SO},srt} + \rho_{a,\text{NSO},srt}$$

3.4.2 Undiagnosed prevalence in women

To inform undiagnosed prevalence in the general population of women, data on new diagnoses during pregnancy are available from the National Study of HIV in Pregnancy and Childhood (NSHPC)[16]. This dataset is a registry of all diagnosed HIV-positive pregnant women, whether they were diagnosed prior to their current pregnancy or during their current pregnancy through antenatal screening. We use the data on women newly diagnosed *during* their current pregnancy to inform prevalence of (previously) undiagnosed infection, using the number of live births in year t as a denominator. The ONS live births data are only available by mother's region of birth[17], rather than by mother's ethnicity; however, we need undiagnosed prevalence by ethnicity. We therefore use indirect information on the relationship between live birth rates in women by ethnicity and in women by region of birth to infer the number of live births by mother's ethnicity, for use as a denominator for the NSHPC new diagnosis data. This sub-model is considered only for the two youngest age groups ($a \in \{$15-34, 35-44$\}$), since the numbers of HIV-positive women giving birth in older age groups are too small, when stratified by age group, region and year, to robustly inform prevalence.

Population by region of birth Specifically, to first obtain an estimate of the population size of women by region of birth (SSA, rest of the world (RW)), we use again the ONS Population Census 2011[18]:

$$y^{(\text{CENSUS})}_{agsr} \sim \text{Binomial}\left(n^{(\text{CENSUS})}_{asr}; \rho^{(\text{CENSUS})}_{agsr}\right), \qquad g = \text{SSA}, s = \text{W}, a < 45$$

where $y^{(\text{CENSUS})}_{a,\text{SSA},\text{W},r}$ is the observed number of women born in SSA, $n^{(\text{CENSUS})}_{a,\text{W},r}$ is the total number of women in the population in 2011, and $\rho^{(\text{CENSUS})}_{a,\text{SSA},\text{W},r}$ the proportion of women born in SSA. This proportion is assumed to be distributed Beta(1,2) *a priori*.

Birth rates and number of live births The live birth rate by mother's region of birth is then informed by the ONS-observed number of live births in year t[17], $y^{(\text{LB})}_{agrt}$, $g \in \{\text{SSA,RW}\}$ and the corresponding estimated population sizes by region of birth:

$$y^{(\text{LB})}_{agrt} \sim \text{Binomial}\left(N_{asrt}\rho^{(\text{CENSUS})}_{agsr}; \zeta_{agrt}\right), \qquad g \in \{\text{SSA,RW}\}, s = \text{W}, a < 45$$

where $\rho^{(\text{CENSUS})}_{a,\text{RW},sr} = 1 - \rho^{(\text{CENSUS})}_{a,\text{SSA},sr}$ and the birth rates ζ_{agrt} are given independent Beta(1,2) prior distributions.

We then make the assumption that live birth rates in the Black African population are the same as in the sub-Saharan African-born population, $\zeta_{a,\text{A},rt} = \zeta_{a,\text{SSA},rt}$. This assumption is prompted by[19], estimating a total fertility rate of 2.32 in the Black African population; and by the ONS live births data release 2015[17], suggesting a total fertility rate in the SSA population varying between 2.4 and 2.8 in 2001-2011. The number of live births to Black African (non-PWID) mothers is then:

$$b_{agrt} = \zeta_{agrt}N_{asrt}\rho_{agsrt}, \qquad g = \text{A}, s = \text{W}$$

To ensure that the sum, over ethnicity, of estimated live births is consistent with the total number of live births, instead of making a similar equality for other heterosexuals, instead we assume

$$b_{a,\text{O},rt} = \left(\sum_{g \in \{\text{SSA,RW}\}} b_{agrt}\right) - b_{a,\text{A},rt}$$

where the numbers of live births by mother's region of birth are obtained from

$$b_{agrt} = \zeta_{agrt}N_{asrt}\rho^{(\text{CENSUS})}_{agsr}, \qquad g \in \{\text{SSA,RW}\}, s = \text{W}, a < 45.$$

Group sizes among pregnant women The proportion of pregnant women who are Black African is defined by the numbers of live births by ethnicity:

$$\rho^{(\text{PW})}_{a,\text{A},rt} = \frac{b_{a,\text{A},rt}}{b_{a,\text{A},rt} + b_{a,\text{O},rt}}$$

and the complementary proportion in the other heterosexual group is $\rho^{(\text{PW})}_{a,\text{O},rt} = \left(1 - \rho^{(\text{PW})}_{a,\text{A},rt}\right)$.

We assume that the proportion of pregnant women who attend SHCs is the same as the proportion of all heterosexual women attending, so that

$$\begin{aligned}
\rho^{(\text{PW})}_{a,\text{SA},rt} &= \xi^{(\text{SHC})}_{a,\text{A},srt}\rho^{(\text{PW})}_{a,\text{A},rt} \\
\rho^{(\text{PW})}_{a,\text{NSA},rt} &= \left(1 - \xi^{(\text{SHC})}_{a,\text{A},srt}\right)\rho^{(\text{PW})}_{a,\text{A},rt} \\
\rho^{(\text{PW})}_{a,\text{SO},rt} &= \xi^{(\text{SHC})}_{a,\text{O},srt}\left(1 - \rho^{(\text{PW})}_{a,\text{A},rt}\right) \\
\rho^{(\text{PW})}_{a,\text{NSO},rt} &= \left(1 - \xi^{(\text{SHC})}_{a,\text{O},srt}\right)\left(1 - \rho^{(\text{PW})}_{a,\text{A},rt}\right), \qquad s = \text{W}, a < 45.
\end{aligned}$$

Undiagnosed prevalence Having defined group sizes in pregnant women, assuming no PWID are pregnant and accounting for the differential fertility by ethnicity, we can now relate previously undiagnosed/newly diagnosed prevalence in pregnant women to undiagnosed prevalence in all women. The number of women newly diagnosed during their current pregnancy, $y^{(\text{NSHPC})}_{agrt}$, by ethnicity, is a binomial sample from the number of live births by ethnicity, with probability parameter $u^{(\text{PW})}_{agrt}$:

$$y^{(\text{NSHPC})}_{agrt} \sim \text{Binomial}\left(b_{agrt}; u^{(\text{PW})}_{agrt}\right), \qquad g \in \{\text{A,O}\}, a < 45.$$

We can then express the previously undiagnosed/newly diagnosed prevalences in pregnant women as weighted averages of the undiagnosed prevalences u_{agsrt} in each of the female non-PWID SHC/non-SHC-attending sub-groups:

$$\begin{aligned}
u^{(\text{PW})}_{a,\text{A},rt} &= \frac{\rho^{(\text{PW})}_{a,\text{SA},rt}u_{a,\text{SA},srt} + \rho^{(\text{PW})}_{a,\text{NSA},rt}u_{a,\text{NSA},srt}}{\rho^{(\text{PW})}_{a,\text{SA},rt} + \rho^{(\text{PW})}_{a,\text{NSA},rt}} \\
u^{(\text{PW})}_{a,\text{O},rt} &= \frac{\rho^{(\text{PW})}_{a,\text{SO},rt}u_{a,\text{SO},srt} + \rho^{(\text{PW})}_{a,\text{NSO},rt}u_{a,\text{NSO},srt}}{\rho^{(\text{PW})}_{a,\text{SO},rt} + \rho^{(\text{PW})}_{a,\text{NSO},rt}}, \qquad s = \text{W}, a < 45.
\end{aligned}$$

3.4.3 Proportion diagnosed in the Black African population

To strengthen the evidence base in lower risk Black African heterosexual men, we incorporate data informing the odds ratio of proportions diagnosed in men versus women, from Sigma Research's African Health and Sex Survey (AHSS) from 2014[20]. We assume that the proportion of surveyed individuals self-reporting a previous HIV test, while not directly informing proportions diagnosed, can indirectly act as a proxy for the male-to-female odds ratio of the proportion diagnosed. The number of individuals ever tested, $y^{(\text{AHSS})}_{sr}$, by gender and region, can be assumed to be binomially distributed with size the number of individuals surveyed, $n^{(\text{AHSS})}_{sr}$ and probability parameter θ_{sr} representing the probability of ever having tested for HIV:

$$y^{(\text{AHSS})}_{sr} \sim \text{Binomial}\left(n^{(\text{AHSS})}_{sr}; \theta_{sr}\right).$$

However, to avoid defining some parameters both stochastically and deterministically, although the AHSS data relate only to 2014, and although age stratification is not available, we repeat the above likelihood term for each age/year combination, so that

$$y^{(\text{AHSS})}_{asrt} \sim \text{Binomial}\left(n^{(\text{AHSS})}_{asrt}; \theta_{asrt}\right).$$

Then we assign a vaguely informative prior to the proportion tested in the female groups:

$$\theta_{a,\mathrm{W},rt} \sim \text{Beta}(2,1)$$

and define the corresponding proportion tested in the male groups in terms of the female groups and a log-odds ratio $\log\left(\alpha_{art}^{(\mathrm{AHSS})}\right)$:

$$\text{logit}(\theta_{a,\mathrm{M},rt}) = \text{logit}(\theta_{a,\mathrm{W},rt}) + \log\left(\alpha_{art}^{(\mathrm{AHSS})}\right).$$

Finally, to inform the male-to-female log odds ratio of proportions diagnosed, we define $\log\left(\alpha_{art}^{(\mathrm{AHSS})}\right)$ as:

$$\log\left(\alpha_{art}^{(\mathrm{AHSS})}\right) = \text{logit}\left(\delta_{ag,\mathrm{M},rt}\right) - \text{logit}\left(\delta_{ag,\mathrm{W},rt}\right), \qquad g = \mathrm{A},$$

where

$$\delta_{a,\mathrm{A},srt} = \frac{\rho_{a,\mathrm{SA},srt}\pi_{a,\mathrm{SA},srt}\delta_{a,\mathrm{SA},srt} + \rho_{a,\mathrm{NSA},srt}\pi_{a,\mathrm{NSA},srt}\delta_{a,\mathrm{NSA},srt}}{\rho_{a,\mathrm{SA},srt}\pi_{a,\mathrm{SA},srt} + \rho_{a,\mathrm{NSA},srt}\pi_{a,\mathrm{NSA},srt}}$$

is the weighted average of the proportions diagnosed in the SHC/non-SHC-attending sub-groups.

3.4.4 Prevalence in the lowest risk group

To strengthen the evidence base in lower risk heterosexual men of other ethnicity, data on HIV prevalence in blood donors[21] is used. As with the AHSS data, we don't use the prevalence data directly, assuming that blood donors in general are lower risk than even the low risk non-SHC-attending heterosexual risk group we consider. Instead, we use the observed male-to-female log-odds ratio of prevalence from the blood donor data to inform the corresponding log-odds ratio of prevalence in the NSO group. To cope with the small numerators of number of HIV infections when stratifying the blood donor data by gender, age, region and year, as well as the corresponding large denominators, we consider the data only by gender and age (two groups only, 15-44 and 45+), summing over regions, and taking the mean prevalence over years. An estimated 2.4% of new male donors and 1.5% of repeat male donors (personal communication[22]) are thought to be GBM, so are excluded from the data. The mean prevalence data are then repeated for each age/year/region combination in the model, with the numbers positive assumed to be binomially distributed with size given by the numbers of blood donors:

$$y_{asrt}^{(\mathrm{BD})} \sim \text{Binomial}\left(n_{asrt}^{(\mathrm{BD})}; \pi_{asrt}^{(\mathrm{BD})}\right)$$

The blood donor prevalence in women, $\pi_{a,\mathrm{W},rt}^{(\mathrm{BD})}$, is given a vaguely informative Beta(1,2) prior; and in men, is defined in terms of the prevalence in women and a log-odds ratio $\log\left(\alpha_{art}^{(\mathrm{BD})}\right)$:

$$\text{logit}\left(\pi_{a,\mathrm{M},rt}^{(\mathrm{BD})}\right) = \text{logit}\left(\pi_{a,\mathrm{W},rt}^{(\mathrm{BD})}\right) + \log\left(\alpha_{art}^{(\mathrm{BD})}\right).$$

This log-odds ratio is, in turn, defined in terms of the male-to-female log odds ratio of prevalence in the lowest risk, non-SHC-attending other heterosexual group:

$$\log\left(\alpha_{art}^{(\mathrm{BD})}\right) = \text{logit}\left(\pi_{a,\mathrm{NSO,M},rt}\right) - \text{logit}\left(\pi_{a,\mathrm{NSO,W},rt}\right).$$

3.4.5 Prevalences and proportion diagnosed in non-SHC-attending groups

As for the NSG group in section 3.1.4, to borrow strength across strata, the prevalence π_{agsrt} and proportion diagnosed δ_{agsrt}, for the two younger age groups in the non-SHC-attending heterosexual groups $\mathcal{G}_{\text{NShet}} = \{\text{NSA,NSO}\}$, are defined in terms of the hierarchical prior defined in section 3.6. The corresponding diagnosed and undiagnosed prevalences in these two younger age groups are then defined in terms of the prevalence and proportion diagnosed:

$$\begin{aligned} d_{agsrt} &= \pi_{agsrt}\delta_{agsrt} \\ u_{agsrt} &= \pi_{agsrt}\left(1 - \delta_{agsrt}\right), \qquad a \in \{\text{15-34, 35-44}\}, g \in \mathcal{G}_{\text{NShet}}. \end{aligned}$$

For the two older age groups, prevalence and the proportion diagnosed are defined, as for the SG group in equation (2) in section 3.1.4, in terms of the corresponding diagnosed and undiagnosed prevalences:

$$\pi_{agsrt} = d_{agsrt} + u_{agsrt}$$
$$\delta_{agsrt} = d_{agsrt}/\pi_{agsrt}, \qquad a \in \{\text{45-59, 60-74}\}, g \in \mathcal{G}_{\text{NShet}},$$

whereas, to compensate for lack of information in the older age groups, these diagnosed and undiagnosed prevalences are subject to age-ordering constraints. As for the NSG group in section 3.1.2, undiagnosed prevalence u_{agsrt} is defined in terms of the age group $a-1$ and a log-odds ratio given a prior such that undiagnosed prevalence decreases with age:

$$\text{logit}\,(u_{agsrt}) = \text{logit}\,(u_{a-1,gsrt}) + \log\left(\alpha^{(\text{age},u)}_{agsrt}\right) \qquad a \in \{\text{45-59, 60-74}\}, g \in \mathcal{G}_{\text{NShet}}$$
$$\log\left(\alpha^{(\text{age},u)}_{agsrt}\right) \sim \text{Normal}(-0.7, 0.3).$$

The age odds-ratio has prior mean 0.5 and prior standard deviation such that the odds ratio lies approximately in (0.3, 0.9).

Similarly (section 3.1.3), diagnosed prevalence d_{agsrt} in the non-SHC-attending heterosexual groups $\mathcal{G}_{\text{NShet}}$ is defined in terms of the age group $a-1$ and a log-odds ratio:

$$\text{logit}\,(d_{agsrt}) = \text{logit}\,(d_{a-1,gsrt}) + \log\left(\alpha^{(\text{age},d)}_{agsrt}\right) \qquad a \in \{\text{45-59, 60-74}\}, g \in \mathcal{G}_{\text{NShet}}$$
$$\log\left(\alpha^{(\text{age},d)}_{agsrt}\right) \sim \text{Normal}(0, 1).$$

However, in contrast to the undiagnosed prevalence age-ordering prior constraint, the age odds-ratio for diagnosed prevalence is given a flatter prior, allowing the age ordering to be determined by the available data on numbers diagnosed (section 3.5) and the rest of the indirect data informing diagnosed prevalence. This flatter prior, on the odds-ratio scale, has mean 1 and standard deviation such that the odds ratio lies approximately in (0.14, 7).

3.5 Number diagnosed, all groups

The HIV/AIDS Reporting System (HARS) database combines information on new HIV diagnoses with data on HIV patients attending clinics for their HIV care. The data therefore provide a yearly snapshot of the number of diagnosed HIV patients alive, resident in the UK, and attending for care. Due to the very high retention in care of HIV patients in the UK, the yearly snapshot is thought to provide complete information on the number of diagnosed HIV patients who are both alive and resident in the UK. Due to good data on transmission route for each of these patients, the data additionally inform the group distribution of diagnosed HIV patients. Nevertheless, to account for any uncertainty about any under-/over-reporting of cases, we allow a little extra uncertainty around the numbers diagnosed, via an adjustment parameter.

The total counts, including those with missing transmission mode information, stratified by age, gender, region and year, are assumed Poisson distributed, with means μ_{asrt}:

$$y^{(\text{HARS})}_{asrt} \sim \text{Poisson}\,(\mu_{asrt})$$

where the means are given by the sums over risk groups of the diagnosed cases:

$$\mu_{asrt} = \sum_{g \in \mathcal{G}_s} \mu_{agsrt},$$

where $\mathcal{G}_s$ refers to the Men or Women's subgroups respectively. The group-specific numbers diagnosed are in turn defined in terms of the proportions of the population at risk, group-specific HIV prevalence and proportion diagnosed.

$$\mu_{agsrt} = \nu_{agsrt} N_{asrt} \rho_{agsrt} \pi_{agsrt} \delta_{agsrt}$$

The adjustment parameter to account for any under-/over-reporting, ν_{agsrt}, is group-specific. For PWID, where under-reporting of injecting transmission risk is thought to be an issue (relative to other modes of transmission), the adjustment parameter is given an informative prior $(1-\nu_{agsrt}) \sim$ Beta(6,34), expressing a prior mean of 15% under-reporting and a 95% prior interval of between 6% and 27% under-reporting. For all other groups, the adjustment is given a prior centred on 1, expressing no adjustment, but with a 90% prior interval allowing for up to plus or minus 3% under- or over-reporting, $\log(\nu_{agsrt}) \sim \mathrm{N}(0, 0.01797^2)$.

The risk group distribution of diagnosed cases is informed by the observed distribution among cases with non-missing (NM) information on transmission mode, assumed to be multinomial:

$$y_{agsrt}^{(\text{HARS,NM})} \sim \text{Multinomial}\left(\mu_{asrt}; \left\{\xi_{agsrt}^{(\text{HARS})}, g \in \{\text{GBM,PWID,A,O}\}\right\}\right).$$

The proportions in each group, $\xi_{agsrt}^{(\text{HARS})}$, are defined in terms of the numbers in each group:

$$\xi_{agsrt}^{(\text{HARS})} = \frac{\mu_{agsrt}}{\mu_{asrt}},$$

where μ_{agsrt} is defined as a sum over the appropriate sub-groups, i.e. GBM = SG + NSG, PWID = CIDU + PIDU, A = SA + NSA, O = SO + NSO. The assumption that the risk group distribution of diagnosed cases is informed by the observed distribution among those with non-missing transmission mode implies that transmission mode is missing at random.

3.6 Hierarchical structure, non-PWID groups

To borrow strength across age-group-gender-region-year strata with more (direct) data than strata with less, and to smooth estimates of both HIV prevalence π_{agsrt} and the proportion diagnosed δ_{agsrt}, we introduce some exchangeability assumptions in a hierarchical structure. It is not thought reasonable that prevalence and proportion diagnosed are similar across strata, but that the log-odds ratios of each in non-SHC-attending groups relative to SHC-attending groups might plausibly be thought similar across strata. The hierarchy is therefore restricted to non-PWID groups, since PWID are not stratified by SHC attendance. We also restrict the hierarchy to the first two age groups, to allow for age-monotonic constraints to be implemented (see sections 3.1.4 and 3.4.5).

We define HIV prevalence and the proportion diagnosed in the non-SHC-attending groups $g \in \mathcal{G}_{\text{NS}} = \{\text{NSG,NSA,NSO}\}$ in terms of the corresponding SHC-attending groups $g \in \mathcal{G}_{\text{S}} = \{\text{SG,SA,SO}\}$ and log-odds ratios:

$$\begin{aligned}\text{logit}\left(\pi_{a,g_{\text{NS}},srt}\right) &= \text{logit}\left(\pi_{a,g_{\text{S}},srt}\right) + \log\left(\alpha_{agsrt}^{(\pi)}\right)\\ \text{logit}\left(\delta_{a,g_{\text{NS}},srt}\right) &= \text{logit}\left(\delta_{a,g_{\text{S}},srt}\right) + \log\left(\alpha_{agsrt}^{(\delta)}\right), \qquad a < 45, g \in \mathcal{G}_{\text{H}} = \{\text{GBM,A,O}\}.\end{aligned}$$

Across regions, these log-odds ratios are distributed around age-group-gender-year-specific means:

$$\begin{aligned}\log\left(\alpha_{agsrt}^{(\pi)}\right) &\sim \text{Normal}\left(\log\left(\alpha_{agst}^{(\pi,\text{region})}\right); \sigma^2_{(\pi,\text{region})}\right)\\ \log\left(\alpha_{agsrt}^{(\delta)}\right) &\sim \text{Normal}\left(\log\left(\alpha_{agst}^{(\delta,\text{region})}\right); \sigma^2_{(\delta,\text{region})}\right),\end{aligned}$$

and these means are in turn distributed around group-gender-year-specific means:

$$\begin{aligned}\log\left(\alpha_{agst}^{(\pi,\text{region})}\right) &\sim \text{Normal}\left(\log\left(\alpha_{gst}^{(\pi,\text{age})}\right); \sigma^2_{(\pi,\text{age})}\right)\\ \log\left(\alpha_{agst}^{(\delta,\text{region})}\right) &\sim \text{Normal}\left(\log\left(\alpha_{gst}^{(\delta,\text{age})}\right); \sigma^2_{(\delta,\text{age})}\right).\end{aligned}$$

Across the five gender-by-group strata (GBM, Black African heterosexual men, Black African women, heterosexual men of other ethnicities, women of other ethnicities), the means across age are distributed around year-specific means:

$$\begin{aligned}\log\left(\alpha_{gst}^{(\pi,\text{age})}\right) &\sim \text{Normal}\left(\log\left(\alpha_{t}^{(\pi,\text{group})}\right); \sigma^2_{(\pi,\text{group})}\right)\\ \log\left(\alpha_{gst}^{(\delta,\text{age})}\right) &\sim \text{Normal}\left(\log\left(\alpha_{t}^{(\delta,\text{group})}\right); \sigma^2_{(\delta,\text{group})}\right).\end{aligned}$$

Finally, to borrow strength across years, we assume the year-specific means follow a random walk:

$$\log\left(\alpha_t^{(\pi,\text{group})}\right) \sim \text{Normal}\left(\log\left(\alpha_{t-1}^{(\pi,\text{group})}\right); \sigma^2_{(\pi,\text{year})}\right)$$
$$\log\left(\alpha_t^{(\delta,\text{group})}\right) \sim \text{Normal}\left(\log\left(\alpha_{t-1}^{(\delta,\text{group})}\right); \sigma^2_{(\delta,\text{year})}\right) \qquad t \geq 2014$$

where $\sigma_{(\pi,\text{year})} = \sigma_{(\delta,\text{year})} = \log(5)/1.96$ and the initial log-odds ratios in year 2013 following a Normal prior:

$$\log\left(\alpha_{2013}^{(\pi,\text{group})}\right) \sim \text{Normal}\left(0; 2^2\right)$$
$$\log\left(\alpha_{2013}^{(\delta,\text{group})}\right) \sim \text{Normal}\left(0; 2^2\right).$$

The hierarchy standard deviations are given informative log-normal prior distributions as follows:

$$\begin{aligned}
\sigma_{(\pi,\text{region})} &\sim \text{LogNormal}\left(-1.50, 0.750^2\right)\\
\sigma_{(\delta,\text{region})} &\sim \text{LogNormal}\left(-2.00, 0.510^2\right)\\
\sigma_{(\pi,\text{age})} &\sim \text{LogNormal}\left(-1.42, 0.805^2\right)\\
\sigma_{(\delta,\text{age})} &\sim \text{LogNormal}\left(-1.42, 0.805^2\right)\\
\sigma_{(\pi,\text{group})} &\sim \text{LogNormal}\left(-1.42, 0.805^2\right)\\
\sigma_{(\delta,\text{group})} &\sim \text{LogNormal}\left(-1.50, 0.750^2\right).
\end{aligned}$$

The interpretation of these priors is as follows:

- A prior mean of $\exp(-1.5) = 0.223$ for a standard deviation σ implies odds ratios α in the interval $[0.65, 1.55]$. A prior standard deviation of 0.75 for $\log(\sigma)$ implies 95% prior mass for σ in $[\exp(-2.97), \exp(-0.03)] = [0.05, 0.97]$; the lower value 0.05 implies odds ratios α in the interval $[0.9, 1.1]$; and the upper value 0.97 implies odds ratios α in the interval $[0.15, 6.7]$.
  - So the odds ratios of prevalence $\alpha_{agsrt}^{(\pi)}$ vary *a priori* across region in the interval $[0.15, 6.7]$; and the odds ratios of proportion diagnosed $\alpha_{gst}^{(\delta,\text{age})}$ vary across group in the same interval.
- A prior mean of $\exp(-2) = 0.135$ for a standard deviation σ implies odds ratios α in the interval $[0.77, 1.3]$. A prior standard deviation of 0.51 for $\log(\sigma)$ implies 95% prior mass for σ in $[\exp(-3), \exp(-1)] = [0.05, 0.37]$; the lower value 0.05 implies odds ratios α in the interval $[0.9, 1.1]$; and the upper value 0.37 implies odds ratios α in the interval $[0.49, 2.06]$.
  - So the odds ratios of proportion diagnosed $\alpha_{agsrt}^{(\delta)}$ vary *a priori* across region in the interval $[0.49, 2.06]$.
- A prior mean of $\exp(-1.42) = 0.242$ for a standard deviation σ implies odds ratios α in the interval $[0.62, 1.61]$. A prior standard deviation of 0.805 for $\log(\sigma)$ implies 95% prior mass for σ in $[\exp(-3), \exp(0.158)] = [0.05, 1.17]$; the lower value 0.05 implies odds ratios α in the interval $[0.9, 1.1]$; and the upper value 1.17 implies odds ratios α in the interval $[0.1, 9.9]$.
  - So both the odds ratios of prevalence $\alpha_{agst}^{(\pi,\text{region})}$ and the odds ratios of proportion diagnosed $\alpha_{agst}^{(\delta,\text{region})}$ vary *a priori* across age in the interval $[0.1, 9.9]$. Likewise, the odds ratios of prevalence $\alpha_{gst}^{(\pi,\text{age})}$ vary across group in the interval $[0.1, 9.9]$.

The structure of the hierarchy for the log-odds ratios of prevalence is schematically given in Figure 4.

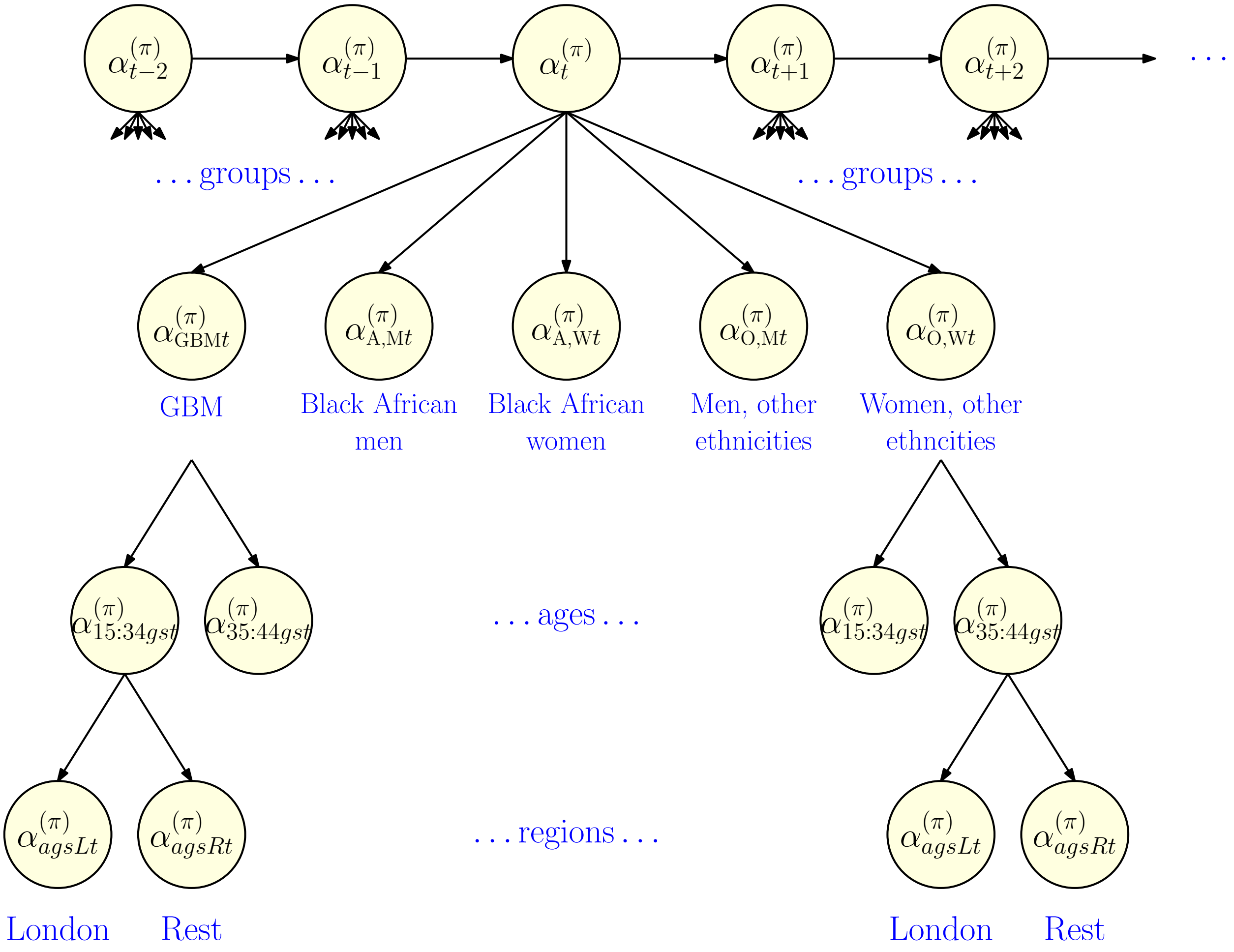

Figure 4: Schematic diagram of hierarchical structure for log-odds ratios of prevalence in non-SHC compared to SHC-attending groups.

4 Selected estimates

Selected estimates are shown in the following plots and tables. The proportion of PLWH whose infection is diagnosed is shown in Figure 5 and Table 14. The numbers of heterosexuals living with undiagnosed HIV by gender are given in Figures 6 and 7, while overall numbers undiagnosed are shown in Table 13. The total number of PLWH is given in Figure 8 and Table 9. The prevalence of undiagnosed HIV is shown in Figures 9 to 11 and Table 12. Exposure group sizes, relative and absolute respectively, are given in Tables 6 and 7. Table 8 lists HIV prevalence by exposure group, region and year. Diagnosed HIV prevalence and the number of people living with diagnosed HIV are shown in Tables 10 and 11 respectively. The proportion of PLWH who remain undiagnosed is given in Table 15.

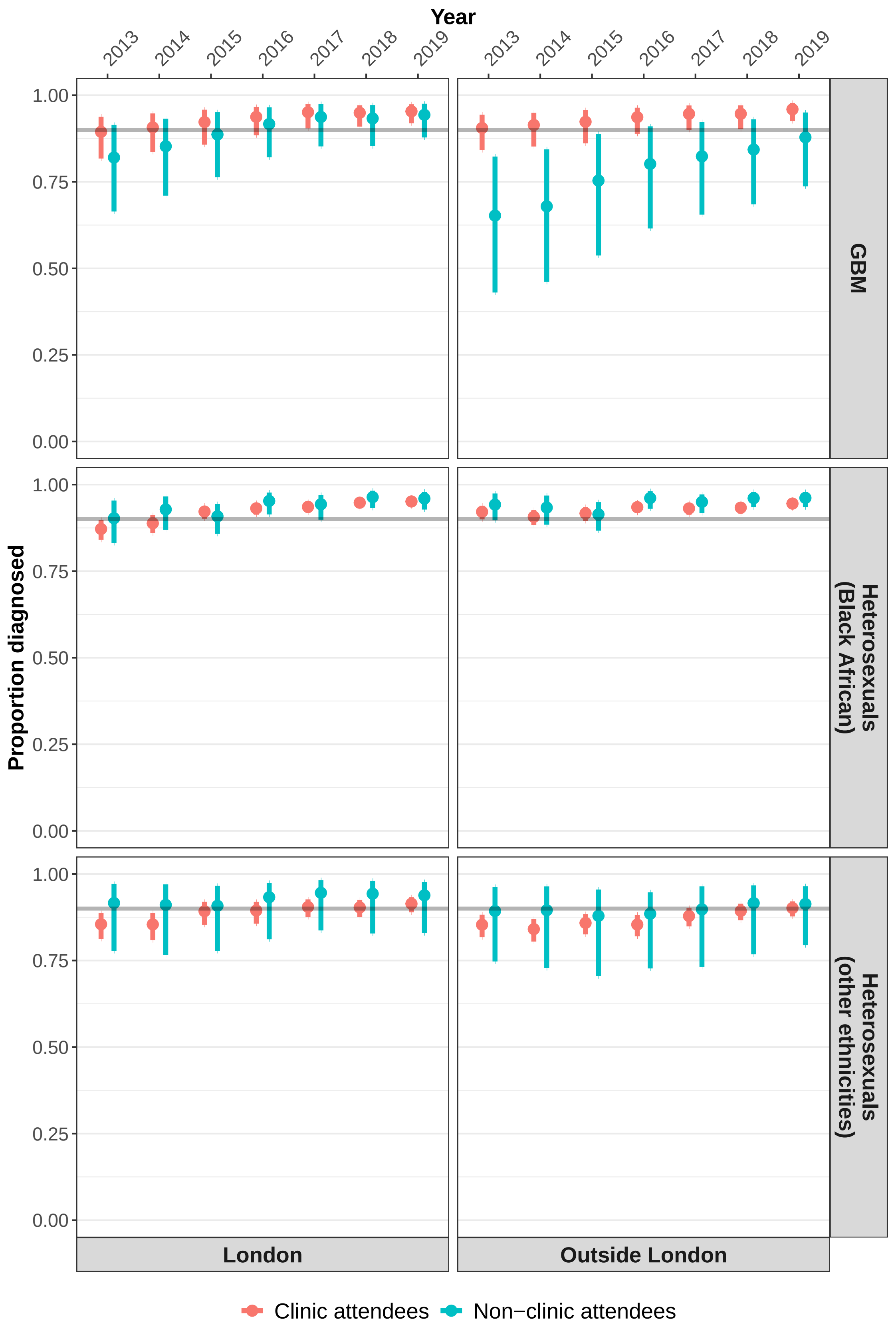

Figure 5: Proportion of PLWH whose infection is diagnosed, by exposure group, SHC attendance, region and year (2013-2019). The UNAIDS 90% proportion diagnosed target is shown by the horizontal grey line.

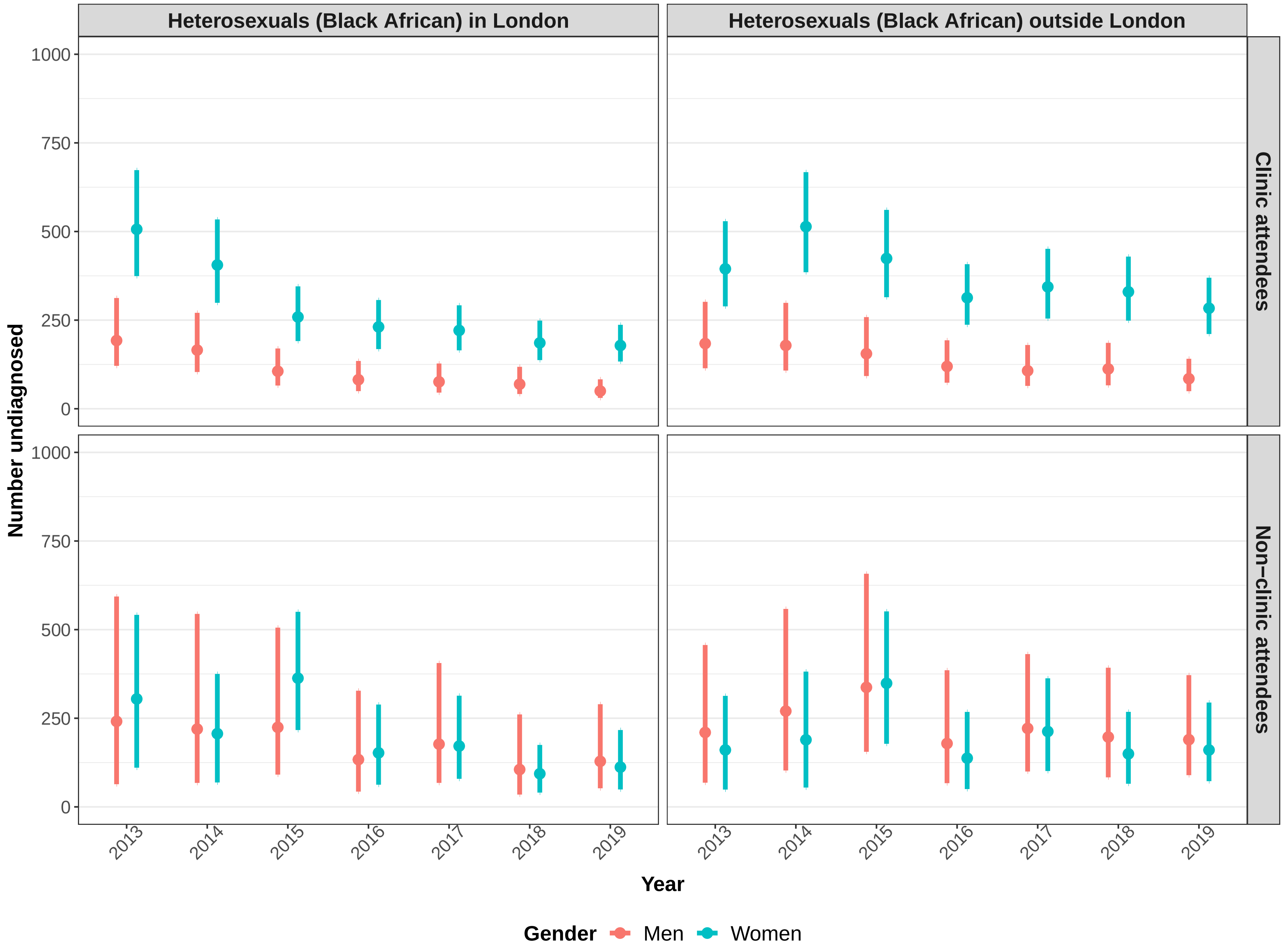

Figure 6: Number of undiagnosed Black African heterosexuals, by SHC attendance, gender, region and year (2013-2019).

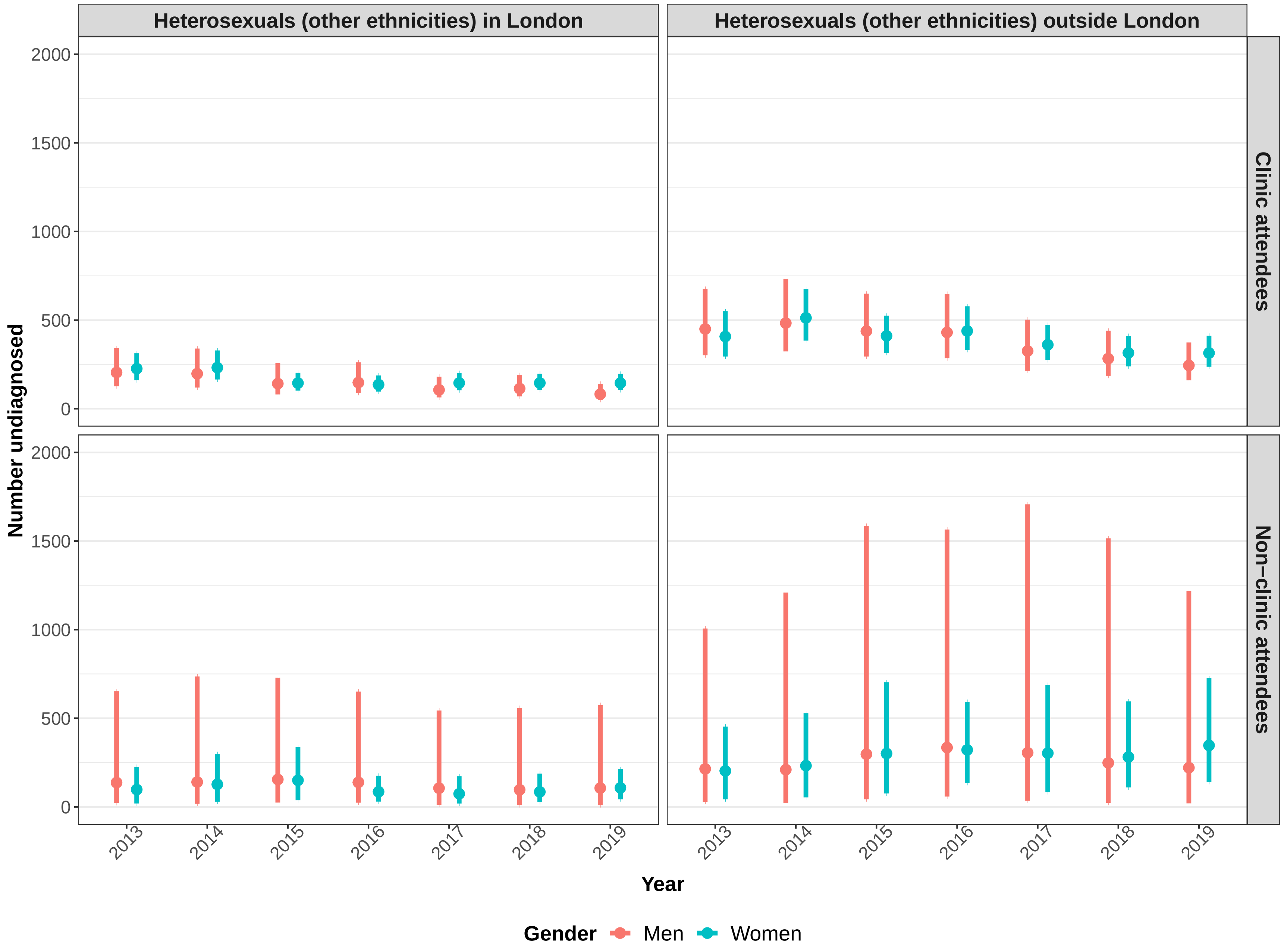

Figure 7: Number of undiagnosed other ethnicity heterosexuals, by SHC attendance, gender, region and year (2013-2019).

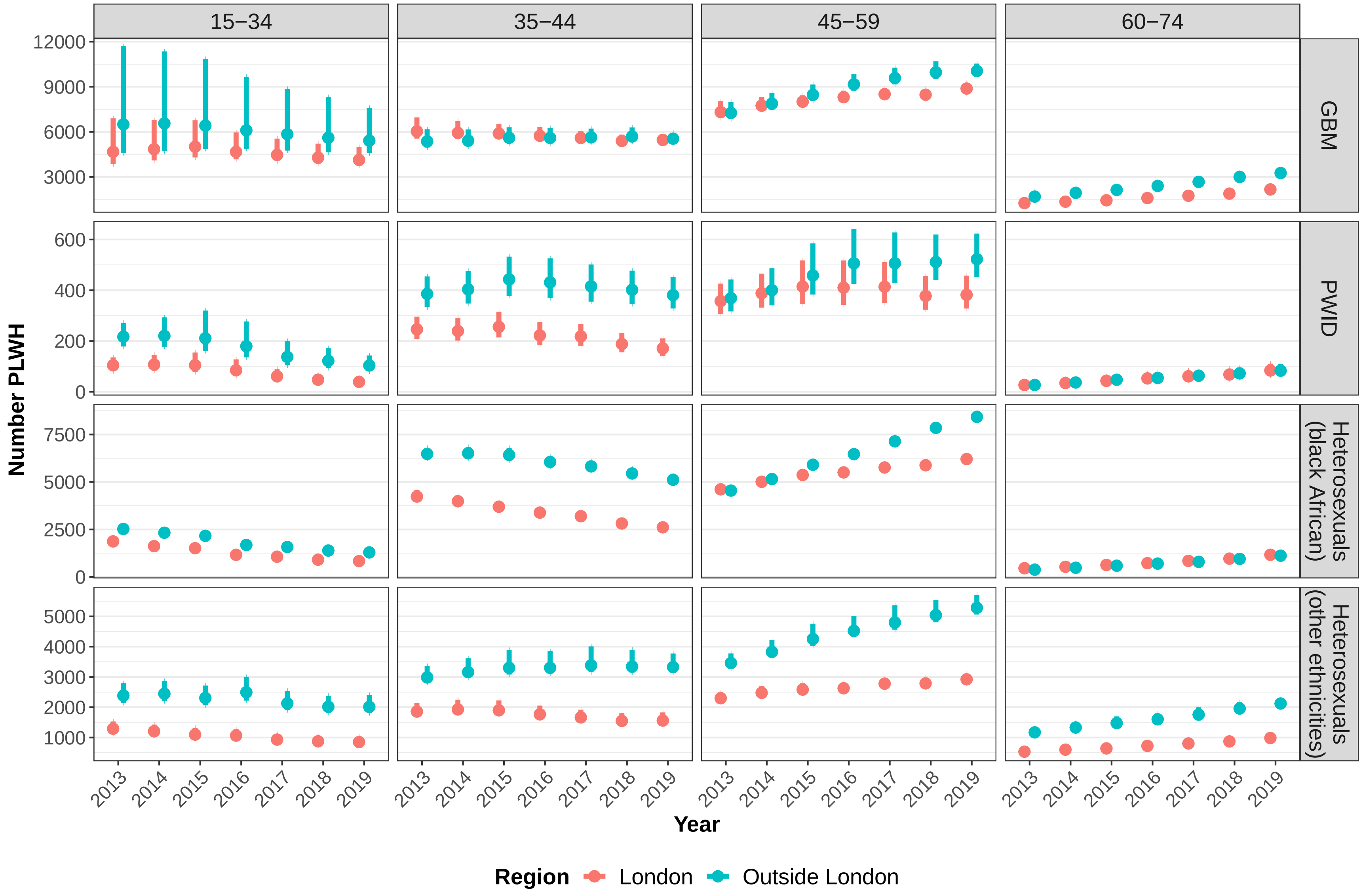

Figure 8: Number of PLWH by exposure group, region, age and year (2013-2019). Note the differing scales of the y-axes by exposure group.

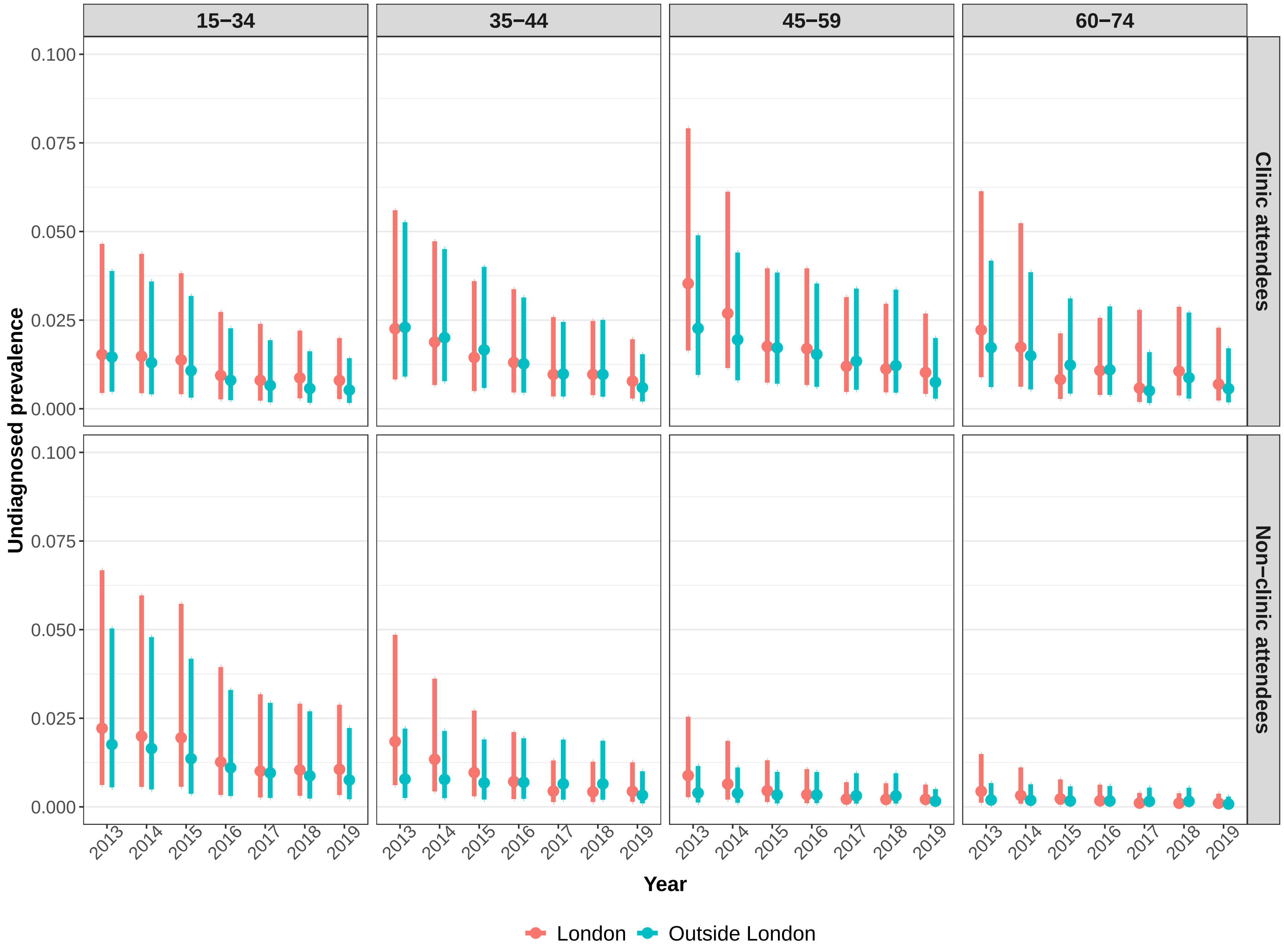

Figure 9: Prevalence of undiagnosed HIV infection among GBM, by SHC attendance, age, region and year (2013-2019).

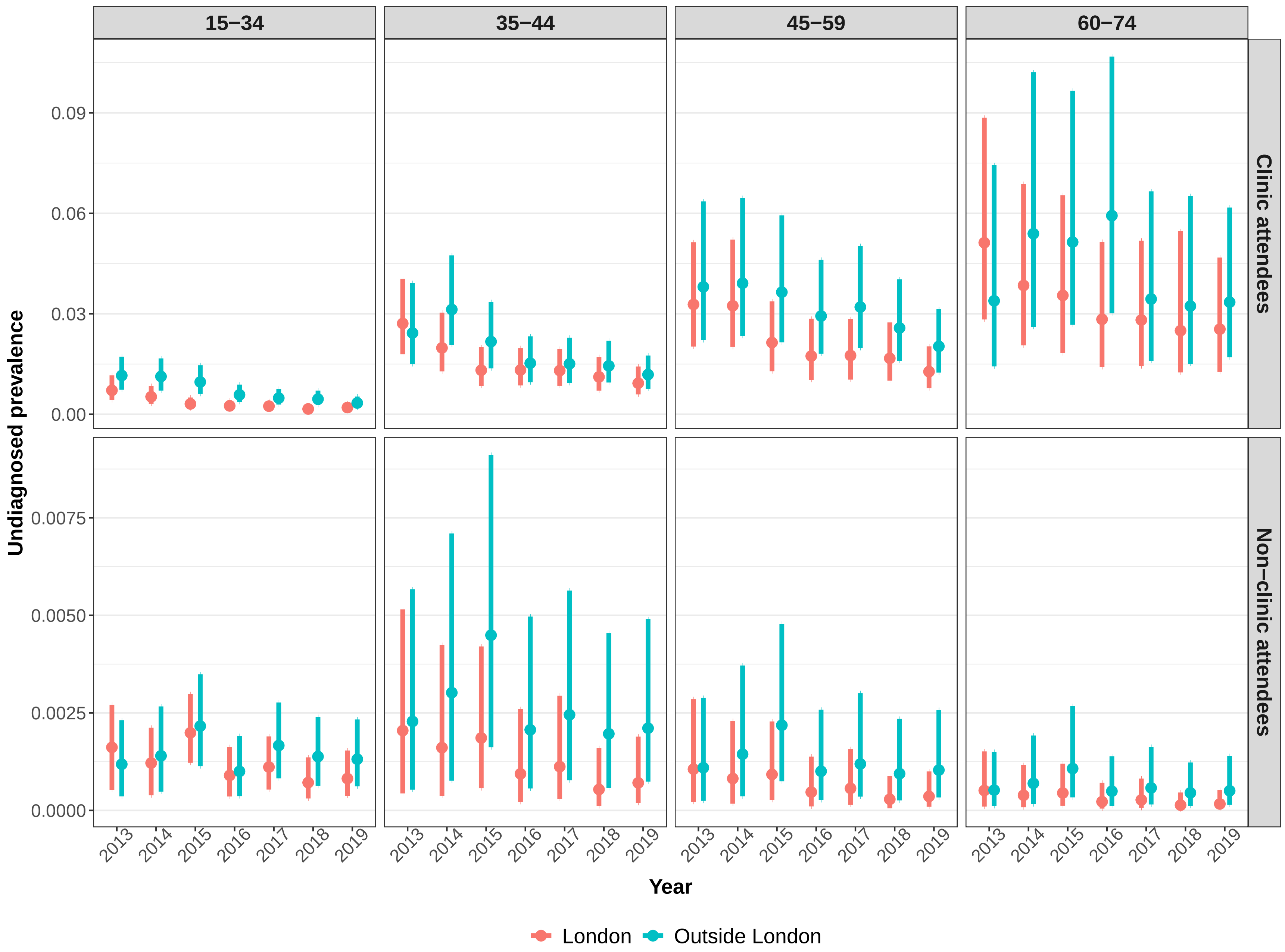

Figure 10: Prevalence of undiagnosed HIV infection among Black African heterosexuals, by SHC attendance, age, region and year (2013-2019). Note the differing y-scales by SHC attendance.

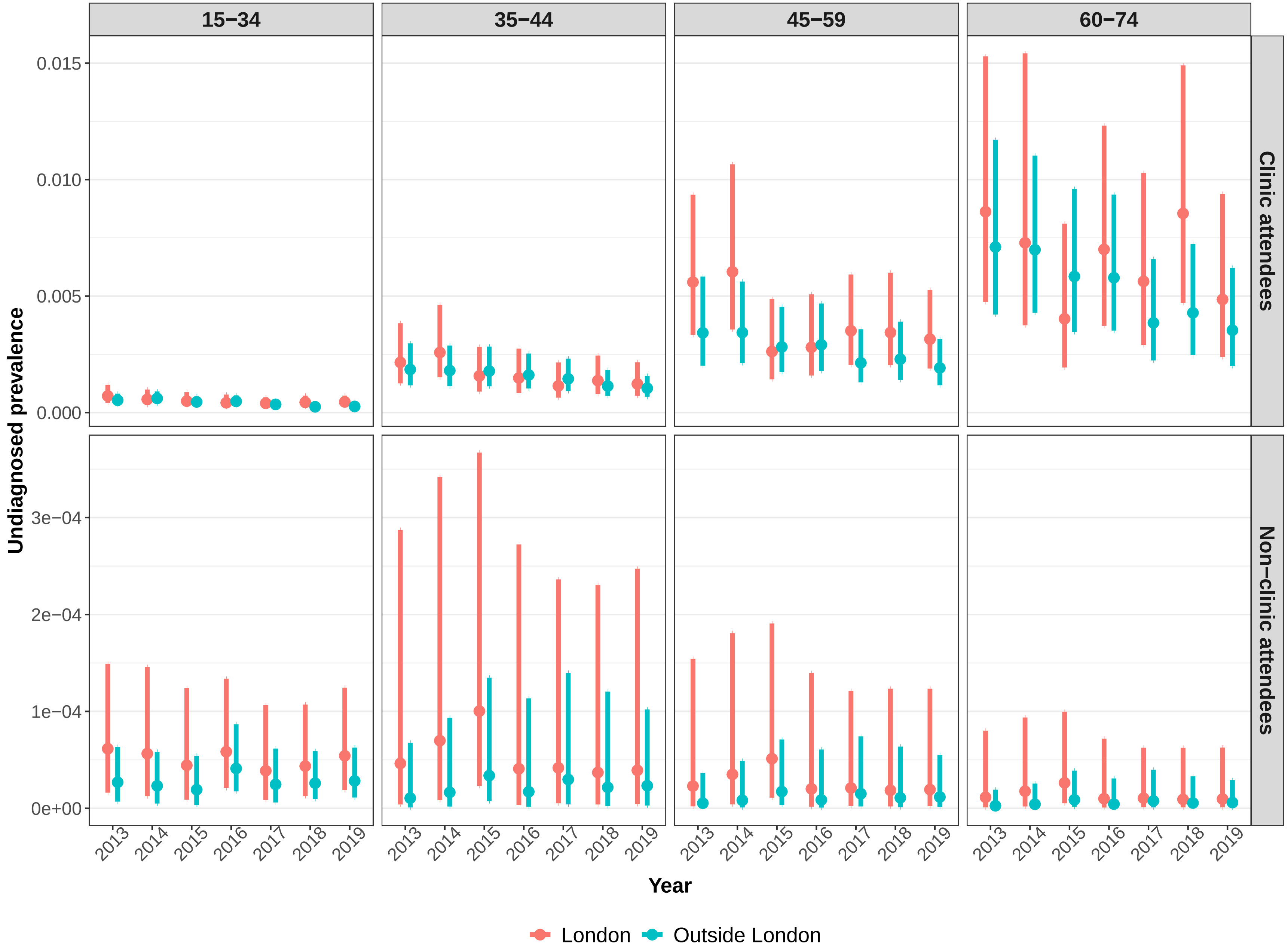

Figure 11: Prevalence of undiagnosed HIV infection among other ethnicity heterosexuals, by SHC attendance, age, region and year (2013-2019). Note the differing y-scales by SHC attendance.

Exposure group		Year	London Median	London 95% CrI		Outside London Median	Outside London 95% CrI		England Median	England 95% CrI	
GBM	Clinic attendees	2013	0.9%	0.9%	0.9%	0.2%	0.2%	0.2%	0.3%	0.3%	0.3%
		2014	1.0%	1.0%	1.0%	0.2%	0.2%	0.2%	0.3%	0.3%	0.3%
		2015	1.0%	1.0%	1.1%	0.2%	0.2%	0.2%	0.3%	0.3%	0.3%
		2016	1.1%	1.1%	1.1%	0.2%	0.2%	0.2%	0.4%	0.4%	0.4%
		2017	1.1%	1.1%	1.1%	0.2%	0.2%	0.2%	0.4%	0.4%	0.4%
		2018	1.1%	1.1%	1.1%	0.3%	0.3%	0.3%	0.4%	0.4%	0.4%
		2019	1.1%	1.1%	1.1%	0.3%	0.3%	0.3%	0.4%	0.4%	0.4%
	Non-clinic attendees	2013	1.4%	1.1%	1.8%	0.9%	0.7%	1.1%	1.0%	0.8%	1.2%
		2014	1.3%	1.0%	1.7%	0.9%	0.7%	1.1%	1.0%	0.8%	1.1%
		2015	1.2%	0.9%	1.6%	0.9%	0.7%	1.1%	0.9%	0.8%	1.1%
		2016	1.2%	0.9%	1.6%	0.9%	0.7%	1.1%	0.9%	0.8%	1.1%
		2017	1.2%	0.9%	1.6%	0.9%	0.7%	1.0%	0.9%	0.8%	1.1%
		2018	1.2%	0.9%	1.6%	0.8%	0.7%	1.0%	0.9%	0.7%	1.1%
		2019	1.2%	0.9%	1.6%	0.8%	0.6%	1.0%	0.9%	0.7%	1.1%
	Total	2013	2.3%	2.0%	2.7%	1.1%	0.9%	1.3%	1.3%	1.1%	1.5%
		2014	2.3%	2.0%	2.7%	1.1%	0.9%	1.3%	1.3%	1.1%	1.5%
		2015	2.3%	2.0%	2.7%	1.1%	0.9%	1.3%	1.3%	1.1%	1.5%
		2016	2.3%	2.0%	2.7%	1.1%	0.9%	1.3%	1.3%	1.1%	1.5%
		2017	2.3%	2.0%	2.7%	1.1%	0.9%	1.3%	1.3%	1.1%	1.5%
		2018	2.3%	2.0%	2.7%	1.1%	0.9%	1.3%	1.3%	1.1%	1.5%
		2019	2.3%	2.0%	2.7%	1.1%	0.9%	1.3%	1.3%	1.1%	1.5%
PWID		2013	0.2%	0.2%	0.2%	0.3%	0.2%	0.3%	0.3%	0.2%	0.3%
		2014	0.2%	0.2%	0.2%	0.3%	0.2%	0.3%	0.3%	0.2%	0.3%
		2015	0.2%	0.2%	0.2%	0.3%	0.2%	0.3%	0.3%	0.2%	0.3%
		2016	0.2%	0.2%	0.2%	0.3%	0.2%	0.3%	0.3%	0.2%	0.3%
		2017	0.2%	0.2%	0.2%	0.3%	0.2%	0.3%	0.3%	0.2%	0.3%
		2018	0.2%	0.2%	0.2%	0.3%	0.2%	0.3%	0.3%	0.2%	0.3%
		2019	0.2%	0.2%	0.2%	0.3%	0.2%	0.3%	0.3%	0.2%	0.3%
Heterosexuals (Black African)	Clinic attendees	2013	0.7%	0.7%	0.7%	0.1%	0.1%	0.1%	0.2%	0.2%	0.2%
		2014	0.7%	0.7%	0.7%	0.1%	0.1%	0.1%	0.2%	0.2%	0.2%
		2015	0.7%	0.7%	0.7%	0.1%	0.1%	0.1%	0.2%	0.2%	0.2%
		2016	0.7%	0.7%	0.7%	0.1%	0.1%	0.1%	0.2%	0.2%	0.2%
		2017	0.7%	0.7%	0.7%	0.1%	0.1%	0.1%	0.2%	0.2%	0.2%
		2018	0.7%	0.6%	0.7%	0.1%	0.1%	0.1%	0.2%	0.2%	0.2%
		2019	0.6%	0.6%	0.7%	0.1%	0.1%	0.1%	0.2%	0.2%	0.2%
	Non-clinic attendees	2013	5.6%	5.6%	5.6%	0.7%	0.7%	0.8%	1.5%	1.5%	1.5%
		2014	5.6%	5.6%	5.6%	0.7%	0.7%	0.7%	1.5%	1.5%	1.5%
		2015	5.6%	5.6%	5.6%	0.7%	0.7%	0.7%	1.5%	1.5%	1.5%
		2016	5.6%	5.6%	5.7%	0.7%	0.7%	0.7%	1.5%	1.5%	1.5%
		2017	5.6%	5.6%	5.7%	0.7%	0.7%	0.7%	1.5%	1.5%	1.5%
		2018	5.6%	5.6%	5.7%	0.7%	0.7%	0.7%	1.5%	1.5%	1.5%
		2019	5.6%	5.6%	5.7%	0.7%	0.7%	0.7%	1.5%	1.5%	1.5%
	Total	2013	6.3%	6.3%	6.4%	0.8%	0.8%	0.8%	1.7%	1.7%	1.7%
		2014	6.3%	6.3%	6.4%	0.8%	0.8%	0.8%	1.7%	1.7%	1.7%
		2015	6.3%	6.3%	6.3%	0.8%	0.8%	0.8%	1.7%	1.7%	1.7%
		2016	6.3%	6.3%	6.3%	0.8%	0.8%	0.8%	1.7%	1.7%	1.7%
		2017	6.3%	6.3%	6.3%	0.8%	0.8%	0.8%	1.7%	1.7%	1.7%
		2018	6.3%	6.3%	6.3%	0.8%	0.8%	0.8%	1.7%	1.7%	1.7%
		2019	6.3%	6.2%	6.3%	0.8%	0.8%	0.8%	1.7%	1.7%	1.7%
Heterosexuals (other ethnicities)	Clinic attendees	2013	4.9%	4.9%	5.0%	2.5%	2.4%	2.5%	2.8%	2.8%	2.9%
		2014	5.0%	4.9%	5.0%	2.7%	2.7%	2.7%	3.0%	3.0%	3.0%
		2015	5.2%	5.1%	5.2%	2.7%	2.7%	2.7%	3.1%	3.1%	3.1%
		2016	5.2%	5.1%	5.2%	2.7%	2.7%	2.7%	3.1%	3.1%	3.1%
		2017	4.7%	4.7%	4.8%	2.8%	2.8%	2.8%	3.1%	3.1%	3.1%
		2018	4.2%	4.2%	4.2%	2.8%	2.8%	2.8%	3.0%	3.0%	3.0%
		2019	3.9%	3.9%	3.9%	2.8%	2.8%	2.8%	3.0%	3.0%	3.0%
	Non-clinic attendees	2013	86.2%	85.9%	86.5%	95.3%	95.1%	95.5%	93.9%	93.7%	94.0%
		2014	86.2%	85.9%	86.5%	95.1%	94.9%	95.3%	93.7%	93.5%	93.9%
		2015	86.0%	85.7%	86.3%	95.1%	94.9%	95.3%	93.6%	93.5%	93.8%
		2016	86.0%	85.7%	86.3%	95.1%	94.9%	95.2%	93.6%	93.4%	93.8%
		2017	86.5%	86.1%	86.7%	95.0%	94.8%	95.2%	93.6%	93.5%	93.8%
		2018	87.0%	86.6%	87.3%	95.0%	94.8%	95.2%	93.7%	93.5%	93.9%
		2019	87.3%	86.9%	87.6%	95.0%	94.8%	95.2%	93.8%	93.6%	93.9%
	Total	2013	91.2%	90.8%	91.5%	97.8%	97.6%	98.0%	96.7%	96.6%	96.9%
		2014	91.2%	90.8%	91.5%	97.8%	97.6%	98.0%	96.7%	96.6%	96.9%
		2015	91.2%	90.8%	91.5%	97.8%	97.6%	98.0%	96.7%	96.5%	96.9%
		2016	91.2%	90.8%	91.5%	97.8%	97.6%	98.0%	96.7%	96.6%	96.9%
		2017	91.2%	90.8%	91.5%	97.8%	97.6%	98.0%	96.7%	96.6%	96.9%
		2018	91.2%	90.8%	91.5%	97.8%	97.6%	98.0%	96.7%	96.6%	96.9%
		2019	91.2%	90.8%	91.5%	97.8%	97.6%	98.0%	96.7%	96.6%	96.9%
Total		2013	100.0%	100.0%	100.0%	100.0%	100.0%	100.0%	100.0%	100.0%	100.0%
		2014	100.0%	100.0%	100.0%	100.0%	100.0%	100.0%	100.0%	100.0%	100.0%
		2015	100.0%	100.0%	100.0%	100.0%	100.0%	100.0%	100.0%	100.0%	100.0%
		2016	100.0%	100.0%	100.0%	100.0%	100.0%	100.0%	100.0%	100.0%	100.0%
		2017	100.0%	100.0%	100.0%	100.0%	100.0%	100.0%	100.0%	100.0%	100.0%
		2018	100.0%	100.0%	100.0%	100.0%	100.0%	100.0%	100.0%	100.0%	100.0%
		2019	100.0%	100.0%	100.0%	100.0%	100.0%	100.0%	100.0%	100.0%	100.0%

Table 6: Posterior estimates by exposure group, region and year of the proportion of the population in each exposure group: posterior median and 95% CrI.

			London			Outside London			England		
Exposure group		***Year***	***Median***	***95% CrI***		***Median***	***95% CrI***		***Median***	***95% CrI***	
GBM	Clinic attendees	2013	55,752	55,408	56,107	59,359	58,934	59,795	115,114	114,560	115,662
		2014	65,478	65,121	65,833	66,931	66,472	67,379	132,412	131,831	132,967
		2015	68,484	68,142	68,837	72,090	71,637	72,556	140,578	139,996	141,151
		2016	69,722	69,373	70,078	73,928	73,457	74,391	143,650	143,062	144,236
		2017	72,238	71,878	72,591	81,826	81,347	82,312	154,064	153,465	154,652
		2018	71,250	70,902	71,608	86,890	86,399	87,378	158,145	157,527	158,752
		2019	73,098	72,741	73,463	94,831	94,319	95,341	167,926	167,309	168,549
	Non-clinic attendees	2013	89,510	70,484	114,076	307,505	251,121	373,403	397,495	332,960	470,973
		2014	82,018	62,753	106,977	301,940	245,201	368,503	384,511	319,315	458,440
		2015	81,446	61,799	106,664	299,080	241,985	366,060	381,106	315,351	455,679
		2016	82,354	62,470	108,080	300,104	242,520	367,397	382,903	316,599	458,110
		2017	80,673	60,603	106,584	293,326	235,383	360,731	374,525	307,924	449,963
		2018	83,376	63,067	109,681	289,167	231,196	356,752	373,061	306,230	448,810
		2019	82,356	61,935	108,870	282,057	223,953	349,802	364,917	297,679	440,759
	Total	2013	145,273	126,277	169,842	366,823	310,422	432,688	512,591	447,891	586,063
		2014	147,499	128,214	172,431	368,905	312,095	435,354	516,925	451,640	590,986
		2015	149,912	130,278	175,214	371,214	314,026	438,227	521,683	455,892	596,129
		2016	152,075	132,151	177,746	373,984	316,399	441,333	526,567	460,213	601,744
		2017	152,875	132,891	178,847	375,143	317,299	442,721	528,564	461,984	604,051
		2018	154,594	134,345	180,866	376,054	318,133	443,696	531,294	464,350	607,076
		2019	155,441	135,019	181,946	376,877	318,853	444,629	532,924	465,693	608,841
PWID		2013	13,202	11,495	15,645	91,257	80,954	110,965	104,630	94,055	124,365
		2014	13,320	11,601	15,777	91,577	81,234	111,360	105,065	94,450	124,872
		2015	13,462	11,726	15,940	91,880	81,502	111,741	105,509	94,859	125,405
		2016	13,550	11,805	16,048	92,234	81,807	112,177	105,951	95,256	125,934
		2017	13,513	11,782	16,005	92,186	81,763	112,114	105,861	95,174	125,844
		2018	13,579	11,846	16,082	92,215	81,791	112,152	105,955	95,266	125,954
		2019	13,581	11,855	16,084	92,289	81,860	112,252	106,040	95,336	126,074
Heterosexuals (Black African)	Clinic attendees	2013	46,161	45,745	46,566	31,813	31,488	32,148	77,972	77,435	78,504
		2014	46,899	46,484	47,325	34,015	33,679	34,358	80,913	80,385	81,466
		2015	46,524	46,118	46,929	35,329	34,984	35,664	81,849	81,311	82,386
		2016	45,334	44,933	45,740	37,295	36,940	37,647	82,633	82,091	83,175
		2017	43,869	43,468	44,264	40,623	40,259	40,988	84,492	83,949	85,035
		2018	43,767	43,371	44,155	43,688	43,313	44,068	87,453	86,906	88,012
		2019	43,401	43,009	43,781	46,495	46,101	46,881	89,896	89,341	90,444
	Non-clinic attendees	2013	356,759	354,990	358,426	251,676	250,472	252,923	608,434	606,184	610,604
		2014	360,724	358,914	362,376	249,647	248,432	250,889	610,365	608,082	612,544
		2015	366,502	364,684	368,199	249,007	247,771	250,255	615,506	613,199	617,730
		2016	371,260	369,421	372,999	247,564	246,315	248,803	618,809	616,500	621,029
		2017	373,647	371,790	375,381	244,085	242,853	245,328	617,734	615,414	619,981
		2018	376,595	374,724	378,326	241,135	239,893	242,398	617,729	615,359	619,962
		2019	378,446	376,574	380,204	238,614	237,380	239,863	617,046	614,702	619,332
	Total	2013	402,915	401,072	404,584	283,491	282,278	284,724	686,401	684,077	688,615
		2014	407,621	405,754	409,316	283,666	282,453	284,900	691,282	688,939	693,513
		2015	413,026	411,139	414,743	284,334	283,119	285,573	697,357	694,991	699,607
		2016	416,596	414,685	418,331	284,857	283,635	286,098	701,449	699,062	703,709
		2017	417,526	415,609	419,272	284,711	283,487	285,952	702,235	699,838	704,498
		2018	420,358	418,428	422,115	284,827	283,603	286,068	705,178	702,770	707,460
		2019	421,847	419,910	423,610	285,107	283,884	286,349	706,948	704,529	709,242
Heterosexuals (other ethnicities)	Clinic attendees	2013	314,360	312,943	315,664	825,313	822,569	827,878	1,139,651	1,136,487	1,142,770
		2014	319,935	318,485	321,240	899,534	896,612	902,157	1,219,451	1,216,074	1,222,525
		2015	337,190	335,739	338,532	918,815	915,981	921,409	1,255,995	1,252,685	1,259,098
		2016	341,075	339,599	342,438	937,047	934,261	939,616	1,278,097	1,274,849	1,281,213
		2017	313,823	312,454	315,116	962,775	960,034	965,287	1,276,578	1,273,466	1,279,570
		2018	280,749	279,509	281,922	971,745	969,104	974,260	1,252,496	1,249,465	1,255,405
		2019	263,426	262,277	264,539	966,593	963,976	969,078	1,230,006	1,227,112	1,232,861
	Non-clinic attendees	2013	5,484,345	5,462,068	5,501,924	32,077,741	32,012,158	32,133,632	37,561,361	37,490,573	37,625,091
		2014	5,551,885	5,529,280	5,569,603	32,180,287	32,114,579	32,236,307	37,731,435	37,659,824	37,795,443
		2015	5,617,607	5,594,617	5,635,779	32,369,614	32,303,289	32,426,221	37,986,387	37,914,481	38,051,409
		2016	5,676,663	5,653,243	5,694,980	32,581,754	32,514,926	32,638,964	38,257,566	38,185,236	38,323,066
		2017	5,729,684	5,705,827	5,748,271	32,692,485	32,625,412	32,749,907	38,421,258	38,348,337	38,486,578
		2018	5,814,987	5,790,648	5,833,844	32,790,418	32,722,817	32,848,133	38,604,461	38,531,295	38,670,651
		2019	5,865,841	5,841,505	5,885,100	32,875,116	32,807,233	32,932,871	38,740,100	38,666,516	38,806,422
	Total	2013	5,798,777	5,775,709	5,816,769	32,903,130	32,835,940	32,960,211	38,701,104	38,627,912	38,766,728
		2014	5,871,826	5,848,351	5,890,148	33,079,880	33,012,212	33,137,476	38,950,803	38,877,345	39,017,005
		2015	5,954,848	5,930,925	5,973,483	33,288,482	33,220,438	33,346,402	39,242,362	39,168,421	39,309,159
		2016	6,017,707	5,993,447	6,036,691	33,518,849	33,450,129	33,577,332	39,535,624	39,460,959	39,602,933
		2017	6,043,460	6,019,010	6,062,679	33,655,293	33,586,188	33,713,965	39,697,818	39,623,079	39,765,506
		2018	6,095,672	6,070,897	6,115,161	33,762,173	33,693,024	33,821,060	39,856,920	39,781,904	39,924,821
		2019	6,129,264	6,104,218	6,148,857	33,841,719	33,772,473	33,900,741	39,970,028	39,894,936	40,038,025
Total		2013	6,360,300	6,360,300	6,360,300	33,645,600	33,645,600	33,645,600	40,005,900	40,005,900	40,005,900
		2014	6,440,400	6,440,400	6,440,400	33,824,900	33,824,900	33,824,900	40,265,300	40,265,300	40,265,300
		2015	6,531,400	6,531,400	6,531,400	34,036,800	34,036,800	34,036,800	40,568,200	40,568,200	40,568,200
		2016	6,600,100	6,600,100	6,600,100	34,270,800	34,270,800	34,270,800	40,870,900	40,870,900	40,870,900
		2017	6,627,600	6,627,600	6,627,600	34,408,200	34,408,200	34,408,200	41,035,800	41,035,800	41,035,800
		2018	6,684,439	6,684,439	6,684,439	34,516,167	34,516,167	34,516,167	41,200,606	41,200,606	41,200,606
		2019	6,720,345	6,720,345	6,720,345	34,596,877	34,596,877	34,596,877	41,317,222	41,317,222	41,317,222

Table 7: Posterior estimates by exposure group, region and year of the size of the population in each exposure group: posterior median and 95% CrI.

			London			Outside London			England		
Exposure group		***Year***	***Median***	***95% CrI***		***Median***	***95% CrI***		***Median***	***95% CrI***	
GBM	Clinic attendees	2013	21.07%	19.62%	23.35%	20.01%	18.87%	21.67%	20.56%	19.57%	21.94%
		2014	20.46%	19.29%	22.33%	19.23%	18.16%	20.77%	19.87%	19.02%	21.11%
		2015	19.63%	18.65%	21.25%	18.11%	17.18%	19.53%	18.88%	18.14%	19.96%
		2016	19.68%	18.87%	20.95%	17.52%	16.78%	18.62%	18.58%	18.01%	19.42%
		2017	19.86%	19.20%	20.95%	16.69%	16.04%	17.69%	18.19%	17.70%	18.92%
		2018	19.91%	19.30%	20.92%	15.87%	15.25%	16.79%	17.71%	17.24%	18.37%
		2019	19.02%	18.44%	19.87%	15.41%	14.95%	16.08%	17.00%	16.61%	17.51%
	Non-clinic attendees	2013	8.62%	6.68%	11.26%	2.99%	2.23%	4.47%	4.27%	3.42%	5.63%
		2014	8.06%	6.16%	10.58%	3.03%	2.29%	4.44%	4.12%	3.31%	5.40%
		2015	8.61%	6.61%	11.29%	3.28%	2.53%	4.59%	4.42%	3.61%	5.64%
		2016	8.12%	6.20%	10.64%	3.51%	2.77%	4.65%	4.50%	3.71%	5.60%
		2017	7.49%	5.69%	9.92%	3.51%	2.77%	4.59%	4.36%	3.58%	5.41%
		2018	7.07%	5.39%	9.27%	3.70%	2.95%	4.75%	4.45%	3.68%	5.45%
		2019	8.24%	6.26%	10.83%	3.48%	2.77%	4.42%	4.55%	3.77%	5.56%
	Total	2013	13.41%	11.33%	15.91%	5.78%	4.74%	7.34%	7.96%	6.84%	9.39%
		2014	13.60%	11.56%	15.92%	6.00%	4.98%	7.52%	8.17%	7.07%	9.59%
		2015	13.67%	11.67%	15.90%	6.18%	5.15%	7.61%	8.34%	7.25%	9.70%
		2016	13.44%	11.50%	15.57%	6.29%	5.26%	7.63%	8.36%	7.30%	9.62%
		2017	13.36%	11.43%	15.42%	6.40%	5.38%	7.68%	8.41%	7.33%	9.64%
		2018	13.01%	11.12%	15.01%	6.53%	5.53%	7.75%	8.41%	7.37%	9.62%
		2019	13.31%	11.38%	15.31%	6.49%	5.51%	7.68%	8.48%	7.44%	9.68%
PWID		2013	5.58%	4.59%	6.69%	1.09%	0.89%	1.29%	1.66%	1.38%	1.91%
		2014	5.80%	4.74%	6.98%	1.16%	0.93%	1.39%	1.75%	1.45%	2.04%
		2015	6.11%	4.96%	7.54%	1.27%	1.01%	1.56%	1.88%	1.54%	2.26%
		2016	5.71%	4.60%	7.05%	1.28%	1.02%	1.57%	1.84%	1.51%	2.21%
		2017	5.60%	4.50%	6.87%	1.22%	0.98%	1.47%	1.78%	1.47%	2.10%
		2018	5.03%	4.12%	6.12%	1.20%	0.97%	1.43%	1.69%	1.41%	1.98%
		2019	4.99%	4.09%	6.02%	1.18%	0.95%	1.40%	1.67%	1.39%	1.93%
Heterosexuals (Black African)	Clinic attendees	2013	11.88%	11.35%	12.48%	23.39%	22.64%	24.25%	16.58%	16.12%	17.09%
		2014	10.96%	10.50%	11.47%	22.00%	21.24%	22.82%	15.60%	15.17%	16.07%
		2015	10.13%	9.78%	10.52%	19.91%	19.27%	20.61%	14.35%	14.00%	14.74%
		2016	10.14%	9.80%	10.51%	17.92%	17.43%	18.46%	13.65%	13.36%	13.97%
		2017	10.62%	10.29%	10.97%	16.24%	15.78%	16.75%	13.33%	13.04%	13.63%
		2018	11.27%	10.95%	11.63%	15.32%	14.89%	15.79%	13.30%	13.03%	13.58%
		2019	10.85%	10.54%	11.17%	14.52%	14.15%	14.92%	12.75%	12.50%	13.00%
	Non-clinic attendees	2013	1.59%	1.47%	1.75%	2.58%	2.42%	2.76%	2.00%	1.89%	2.14%
		2014	1.67%	1.56%	1.80%	2.80%	2.63%	3.01%	2.13%	2.02%	2.26%
		2015	1.77%	1.67%	1.89%	3.23%	3.05%	3.44%	2.36%	2.25%	2.49%
		2016	1.66%	1.58%	1.77%	3.32%	3.17%	3.49%	2.33%	2.24%	2.43%
		2017	1.66%	1.57%	1.77%	3.58%	3.42%	3.76%	2.42%	2.33%	2.53%
		2018	1.50%	1.42%	1.58%	3.71%	3.55%	3.88%	2.36%	2.28%	2.45%
		2019	1.61%	1.53%	1.70%	3.86%	3.70%	4.03%	2.48%	2.40%	2.57%
	Total	2013	2.77%	2.67%	2.90%	4.92%	4.78%	5.07%	3.66%	3.57%	3.77%
		2014	2.74%	2.65%	2.85%	5.10%	4.96%	5.27%	3.71%	3.62%	3.82%
		2015	2.71%	2.63%	2.82%	5.31%	5.15%	5.49%	3.77%	3.68%	3.88%
		2016	2.59%	2.51%	2.68%	5.23%	5.10%	5.37%	3.66%	3.59%	3.75%
		2017	2.60%	2.53%	2.70%	5.39%	5.26%	5.54%	3.73%	3.66%	3.82%
		2018	2.51%	2.45%	2.59%	5.49%	5.36%	5.63%	3.72%	3.65%	3.79%
		2019	2.56%	2.50%	2.64%	5.60%	5.47%	5.74%	3.79%	3.72%	3.87%
Heterosexuals (other ethnicities)	Clinic attendees	2013	0.96%	0.90%	1.02%	0.71%	0.68%	0.75%	0.78%	0.75%	0.82%
		2014	0.93%	0.88%	1.01%	0.70%	0.67%	0.74%	0.76%	0.73%	0.80%
		2015	0.80%	0.76%	0.85%	0.66%	0.63%	0.69%	0.70%	0.67%	0.72%
		2016	0.80%	0.76%	0.85%	0.64%	0.61%	0.67%	0.68%	0.66%	0.71%
		2017	0.85%	0.81%	0.90%	0.59%	0.57%	0.62%	0.66%	0.64%	0.68%
		2018	0.97%	0.92%	1.02%	0.58%	0.56%	0.61%	0.67%	0.65%	0.69%
		2019	1.01%	0.97%	1.06%	0.59%	0.57%	0.62%	0.68%	0.66%	0.70%
	Non-clinic attendees	2013	0.05%	0.05%	0.06%	0.01%	0.01%	0.02%	0.02%	0.02%	0.02%
		2014	0.06%	0.05%	0.07%	0.01%	0.01%	0.02%	0.02%	0.02%	0.02%
		2015	0.06%	0.06%	0.07%	0.02%	0.01%	0.02%	0.02%	0.02%	0.03%
		2016	0.06%	0.06%	0.07%	0.02%	0.02%	0.02%	0.02%	0.02%	0.03%
		2017	0.06%	0.06%	0.07%	0.02%	0.02%	0.02%	0.03%	0.02%	0.03%
		2018	0.06%	0.05%	0.07%	0.02%	0.02%	0.02%	0.03%	0.02%	0.03%
		2019	0.06%	0.06%	0.07%	0.02%	0.02%	0.02%	0.03%	0.03%	0.03%
	Total	2013	0.10%	0.10%	0.11%	0.03%	0.03%	0.03%	0.04%	0.04%	0.04%
		2014	0.11%	0.10%	0.12%	0.03%	0.03%	0.04%	0.04%	0.04%	0.05%
		2015	0.10%	0.10%	0.11%	0.03%	0.03%	0.04%	0.04%	0.04%	0.05%
		2016	0.10%	0.10%	0.11%	0.04%	0.03%	0.04%	0.05%	0.04%	0.05%
		2017	0.10%	0.10%	0.11%	0.04%	0.03%	0.04%	0.05%	0.04%	0.05%
		2018	0.10%	0.10%	0.11%	0.04%	0.04%	0.04%	0.05%	0.04%	0.05%
		2019	0.10%	0.10%	0.11%	0.04%	0.04%	0.04%	0.05%	0.05%	0.05%
Total		2013	0.59%	0.56%	0.63%	0.14%	0.13%	0.15%	0.21%	0.20%	0.22%
		2014	0.59%	0.57%	0.63%	0.14%	0.14%	0.16%	0.22%	0.21%	0.23%
		2015	0.59%	0.57%	0.62%	0.15%	0.14%	0.16%	0.22%	0.21%	0.23%
		2016	0.58%	0.56%	0.60%	0.15%	0.14%	0.16%	0.22%	0.21%	0.23%
		2017	0.58%	0.56%	0.60%	0.15%	0.15%	0.16%	0.22%	0.22%	0.23%
		2018	0.56%	0.55%	0.58%	0.16%	0.15%	0.16%	0.22%	0.22%	0.23%
		2019	0.57%	0.56%	0.59%	0.16%	0.15%	0.16%	0.22%	0.22%	0.23%

Table 8: Posterior estimates by exposure group, region and year of HIV prevalence: posterior median and 95% CrI.

			London			Outside London			England		
Exposure group		*Year*	*Median*	*95% CrI*		*Median*	*95% CrI*		*Median*	*95% CrI*	
GBM	Clinic attendees	2013	11,746	10,942	13,006	11,880	11,200	12,879	23,663	22,517	25,260
		2014	13,401	12,627	14,634	12,869	12,154	13,912	26,309	25,180	27,956
		2015	13,444	12,760	14,563	13,057	12,376	14,077	26,543	25,492	28,054
		2016	13,720	13,155	14,604	12,948	12,397	13,765	26,695	25,867	27,903
		2017	14,348	13,865	15,135	13,659	13,122	14,473	28,028	27,248	29,152
		2018	14,190	13,745	14,906	13,785	13,242	14,592	28,001	27,259	29,066
		2019	13,902	13,469	14,533	14,616	14,168	15,256	28,541	27,879	29,427
	Non-clinic attendees	2013	7,650	6,631	9,525	9,078	7,117	13,731	16,863	14,337	21,907
		2014	6,561	5,740	7,963	9,041	7,220	13,284	15,689	13,459	20,260
		2015	6,976	6,206	8,179	9,687	8,090	13,564	16,738	14,788	20,890
		2016	6,654	6,023	7,588	10,424	9,027	13,541	17,111	15,496	20,367
		2017	6,014	5,484	6,748	10,178	8,921	12,811	16,230	14,766	19,005
		2018	5,876	5,337	6,560	10,574	9,392	12,989	16,473	15,115	19,058
		2019	6,768	6,226	7,440	9,698	8,753	11,543	16,502	15,324	18,448
	Total	2013	19,391	18,058	22,047	20,956	18,635	26,262	40,523	37,422	46,614
		2014	19,956	18,777	22,212	21,913	19,717	26,851	42,016	39,210	47,670
		2015	20,418	19,374	22,345	22,730	20,817	27,301	43,276	40,820	48,384
		2016	20,363	19,521	21,870	23,356	21,729	26,975	43,817	41,830	47,838
		2017	20,350	19,669	21,644	23,833	22,383	26,999	44,258	42,483	47,730
		2018	20,067	19,383	21,174	24,355	22,986	27,318	44,472	42,827	47,637
		2019	20,668	20,015	21,667	24,311	23,223	26,569	45,036	43,628	47,533
PWID		2013	737	663	831	1,002	910	1,122	1,741	1,613	1,906
		2014	773	691	881	1,066	959	1,213	1,839	1,690	2,051
		2015	822	722	968	1,166	1,026	1,384	1,988	1,789	2,313
		2016	773	676	920	1,178	1,040	1,407	1,952	1,750	2,282
		2017	757	667	885	1,128	1,006	1,311	1,886	1,714	2,159
		2018	683	610	783	1,112	1,001	1,271	1,795	1,650	2,015
		2019	678	607	772	1,094	992	1,240	1,773	1,636	1,970
Heterosexuals (Black African)	Clinic attendees	2013	5,485	5,239	5,765	7,443	7,187	7,725	12,930	12,553	13,337
		2014	5,141	4,920	5,384	7,482	7,213	7,770	12,625	12,267	13,017
		2015	4,714	4,545	4,901	7,035	6,801	7,290	11,747	11,459	12,075
		2016	4,599	4,439	4,769	6,682	6,489	6,896	11,282	11,032	11,556
		2017	4,659	4,506	4,818	6,599	6,400	6,807	11,260	11,008	11,523
		2018	4,933	4,787	5,096	6,695	6,499	6,902	11,629	11,386	11,892
		2019	4,710	4,570	4,855	6,754	6,573	6,945	11,464	11,226	11,700
	Non-clinic attendees	2013	5,680	5,245	6,231	6,494	6,084	6,940	12,181	11,493	12,991
		2014	6,012	5,614	6,499	6,991	6,550	7,502	13,003	12,334	13,816
		2015	6,491	6,108	6,941	8,054	7,590	8,580	14,552	13,872	15,334
		2016	6,178	5,858	6,555	8,217	7,841	8,632	14,396	13,866	15,021
		2017	6,209	5,874	6,611	8,731	8,340	9,167	14,941	14,380	15,606
		2018	5,631	5,346	5,944	8,943	8,562	9,353	14,578	14,072	15,114
		2019	6,106	5,803	6,442	9,203	8,820	9,610	15,315	14,799	15,869
	Total	2013	11,170	10,773	11,693	13,936	13,568	14,371	25,111	24,510	25,860
		2014	11,149	10,790	11,624	14,477	14,073	14,959	25,627	25,039	26,363
		2015	11,207	10,851	11,646	15,088	14,648	15,611	26,301	25,677	27,034
		2016	10,776	10,477	11,145	14,899	14,550	15,306	25,680	25,187	26,273
		2017	10,867	10,550	11,257	15,331	14,970	15,757	26,204	25,677	26,822
		2018	10,565	10,294	10,873	15,641	15,275	16,040	26,207	25,737	26,728
		2019	10,814	10,528	11,141	15,957	15,602	16,351	26,775	26,286	27,317
Heterosexuals (other ethnicities)	Clinic attendees	2013	3,012	2,835	3,215	5,896	5,606	6,222	8,908	8,561	9,301
		2014	2,983	2,801	3,219	6,290	5,986	6,646	9,283	8,907	9,702
		2015	2,698	2,556	2,870	6,026	5,766	6,334	8,729	8,424	9,083
		2016	2,715	2,576	2,881	5,991	5,717	6,309	8,712	8,396	9,065
		2017	2,682	2,556	2,825	5,698	5,464	5,965	8,384	8,112	8,679
		2018	2,718	2,594	2,853	5,646	5,421	5,893	8,365	8,103	8,646
		2019	2,667	2,548	2,797	5,728	5,515	5,961	8,397	8,146	8,665
	Non-clinic attendees	2013	2,975	2,691	3,524	4,119	3,697	4,962	7,094	6,501	8,369
		2014	3,226	2,917	3,888	4,491	4,035	5,523	7,720	7,072	9,245
		2015	3,528	3,222	4,143	5,329	4,789	6,648	8,859	8,121	10,640
		2016	3,471	3,202	4,014	5,951	5,430	7,234	9,418	8,758	11,118
		2017	3,503	3,252	3,981	6,375	5,833	7,837	9,878	9,190	11,703
		2018	3,376	3,123	3,854	6,722	6,221	8,037	10,094	9,458	11,760
		2019	3,656	3,396	4,160	7,030	6,522	8,120	10,686	10,043	12,167
	Total	2013	5,988	5,712	6,543	10,018	9,582	10,894	16,010	15,427	17,304
		2014	6,215	5,923	6,871	10,785	10,312	11,832	17,002	16,365	18,538
		2015	6,228	5,929	6,840	11,357	10,822	12,712	17,592	16,859	19,376
		2016	6,187	5,930	6,734	11,944	11,431	13,264	18,137	17,490	19,803
		2017	6,187	5,943	6,663	12,074	11,540	13,581	18,261	17,594	20,100
		2018	6,092	5,845	6,575	12,367	11,877	13,666	18,453	17,850	20,113
		2019	6,324	6,076	6,824	12,759	12,281	13,838	19,085	18,477	20,553
Total		2013	37,350	35,855	40,047	46,004	43,500	51,344	83,507	80,219	89,626
		2014	38,170	36,817	40,523	48,335	45,952	53,412	86,618	83,493	92,605
		2015	38,744	37,522	40,807	50,495	48,291	55,262	89,343	86,528	94,588
		2016	38,158	37,176	39,760	51,509	49,619	55,338	89,752	87,390	93,957
		2017	38,212	37,364	39,579	52,497	50,737	55,905	90,779	88,630	94,579
		2018	37,443	36,637	38,634	53,605	51,963	56,715	91,100	89,104	94,488
		2019	38,521	37,725	39,685	54,211	52,848	56,655	92,769	91,021	95,583

Table 9: Posterior estimates by exposure group, region and year of number of people living with HIV: posterior median and 95% CrI.

Exposure group		Year	London Median	London 95% CrI		Outside London Median	Outside London 95% CrI		England Median	England 95% CrI	
GBM	Clinic attendees	2013	18.81%	17.98%	19.68%	18.09%	17.40%	18.85%	18.45%	17.86%	19.07%
		2014	18.54%	17.92%	19.19%	17.55%	16.87%	18.26%	18.04%	17.54%	18.55%
		2015	18.10%	17.53%	18.65%	16.69%	16.12%	17.30%	17.38%	16.94%	17.81%
		2016	18.43%	17.96%	18.91%	16.39%	15.90%	16.91%	17.38%	17.02%	17.75%
		2017	18.87%	18.48%	19.26%	15.77%	15.34%	16.26%	17.22%	16.92%	17.55%
		2018	18.90%	18.53%	19.31%	15.00%	14.59%	15.46%	16.76%	16.46%	17.08%
		2019	18.12%	17.76%	18.52%	14.78%	14.46%	15.12%	16.23%	15.98%	16.50%
	Non-clinic attendees	2013	7.00%	5.46%	8.94%	1.92%	1.55%	2.39%	3.06%	2.54%	3.72%
		2014	6.80%	5.17%	8.98%	2.04%	1.63%	2.54%	3.04%	2.52%	3.71%
		2015	7.56%	5.75%	10.05%	2.44%	1.96%	3.06%	3.53%	2.91%	4.29%
		2016	7.38%	5.60%	9.74%	2.78%	2.25%	3.46%	3.76%	3.13%	4.58%
		2017	6.97%	5.24%	9.28%	2.86%	2.30%	3.58%	3.74%	3.09%	4.57%
		2018	6.54%	4.96%	8.70%	3.08%	2.47%	3.87%	3.85%	3.18%	4.71%
		2019	7.73%	5.82%	10.29%	3.01%	2.41%	3.83%	4.07%	3.35%	5.01%
	Total	2013	11.53%	9.87%	13.29%	4.55%	3.84%	5.37%	6.52%	5.70%	7.47%
		2014	12.02%	10.28%	13.84%	4.85%	4.11%	5.73%	6.89%	6.03%	7.88%
		2015	12.37%	10.59%	14.27%	5.21%	4.41%	6.17%	7.26%	6.35%	8.32%
		2016	12.45%	10.65%	14.32%	5.47%	4.63%	6.48%	7.48%	6.54%	8.56%
		2017	12.59%	10.76%	14.48%	5.68%	4.81%	6.71%	7.67%	6.71%	8.78%
		2018	12.24%	10.46%	14.11%	5.83%	4.95%	6.91%	7.69%	6.74%	8.80%
		2019	12.61%	10.78%	14.54%	5.98%	5.06%	7.08%	7.90%	6.92%	9.05%
PWID		2013	5.33%	4.39%	6.34%	1.04%	0.85%	1.22%	1.58%	1.32%	1.80%
		2014	5.43%	4.47%	6.45%	1.08%	0.88%	1.27%	1.63%	1.36%	1.86%
		2015	5.43%	4.47%	6.46%	1.12%	0.91%	1.30%	1.67%	1.39%	1.89%
		2016	5.07%	4.18%	6.01%	1.12%	0.91%	1.31%	1.63%	1.36%	1.85%
		2017	5.12%	4.20%	6.11%	1.11%	0.90%	1.30%	1.63%	1.35%	1.85%
		2018	4.77%	3.94%	5.70%	1.14%	0.93%	1.33%	1.60%	1.34%	1.82%
		2019	4.81%	3.97%	5.73%	1.14%	0.92%	1.33%	1.61%	1.34%	1.83%
Heterosexuals (Black African)	Clinic attendees	2013	10.36%	10.00%	10.73%	21.55%	20.96%	22.15%	14.92%	14.60%	15.26%
		2014	9.72%	9.38%	10.09%	19.94%	19.41%	20.49%	14.02%	13.71%	14.34%
		2015	9.34%	9.06%	9.64%	18.25%	17.79%	18.71%	13.19%	12.92%	13.45%
		2016	9.45%	9.17%	9.74%	16.74%	16.34%	17.16%	12.74%	12.50%	12.99%
		2017	9.93%	9.65%	10.22%	15.12%	14.75%	15.51%	12.43%	12.20%	12.67%
		2018	10.68%	10.40%	10.98%	14.30%	13.95%	14.66%	12.49%	12.26%	12.72%
		2019	10.32%	10.04%	10.60%	13.73%	13.41%	14.04%	12.08%	11.87%	12.29%
	Non-clinic attendees	2013	1.44%	1.36%	1.52%	2.43%	2.30%	2.56%	1.85%	1.77%	1.92%
		2014	1.54%	1.46%	1.62%	2.61%	2.48%	2.74%	1.98%	1.91%	2.05%
		2015	1.61%	1.53%	1.68%	2.95%	2.82%	3.08%	2.15%	2.08%	2.22%
		2016	1.58%	1.51%	1.66%	3.19%	3.06%	3.32%	2.23%	2.16%	2.29%
		2017	1.57%	1.49%	1.64%	3.39%	3.26%	3.53%	2.29%	2.22%	2.36%
		2018	1.44%	1.37%	1.51%	3.56%	3.42%	3.70%	2.27%	2.20%	2.34%
		2019	1.55%	1.48%	1.62%	3.70%	3.57%	3.85%	2.38%	2.31%	2.45%
	Total	2013	2.46%	2.40%	2.52%	4.57%	4.48%	4.67%	3.33%	3.28%	3.39%
		2014	2.49%	2.43%	2.54%	4.69%	4.59%	4.79%	3.39%	3.34%	3.44%
		2015	2.48%	2.42%	2.54%	4.85%	4.75%	4.95%	3.45%	3.39%	3.50%
		2016	2.44%	2.38%	2.50%	4.96%	4.86%	5.06%	3.46%	3.41%	3.52%
		2017	2.44%	2.39%	2.50%	5.07%	4.97%	5.17%	3.51%	3.45%	3.56%
		2018	2.40%	2.35%	2.46%	5.21%	5.10%	5.32%	3.54%	3.48%	3.59%
		2019	2.45%	2.39%	2.51%	5.34%	5.23%	5.45%	3.61%	3.56%	3.67%
Heterosexuals (other ethnicities)	Clinic attendees	2013	0.82%	0.78%	0.86%	0.61%	0.59%	0.63%	0.67%	0.65%	0.69%
		2014	0.80%	0.76%	0.84%	0.59%	0.57%	0.61%	0.64%	0.62%	0.66%
		2015	0.71%	0.68%	0.75%	0.56%	0.54%	0.58%	0.60%	0.59%	0.62%
		2016	0.71%	0.68%	0.74%	0.55%	0.53%	0.57%	0.59%	0.57%	0.61%
		2017	0.77%	0.74%	0.81%	0.52%	0.50%	0.54%	0.58%	0.57%	0.60%
		2018	0.87%	0.84%	0.91%	0.52%	0.50%	0.54%	0.60%	0.58%	0.61%
		2019	0.92%	0.89%	0.96%	0.53%	0.52%	0.55%	0.62%	0.60%	0.63%
	Non-clinic attendees	2013	0.05%	0.05%	0.05%	0.01%	0.01%	0.01%	0.02%	0.02%	0.02%
		2014	0.05%	0.05%	0.06%	0.01%	0.01%	0.01%	0.02%	0.02%	0.02%
		2015	0.06%	0.05%	0.06%	0.01%	0.01%	0.02%	0.02%	0.02%	0.02%
		2016	0.06%	0.05%	0.06%	0.02%	0.02%	0.02%	0.02%	0.02%	0.02%
		2017	0.06%	0.05%	0.06%	0.02%	0.02%	0.02%	0.02%	0.02%	0.02%
		2018	0.05%	0.05%	0.06%	0.02%	0.02%	0.02%	0.02%	0.02%	0.03%
		2019	0.06%	0.06%	0.06%	0.02%	0.02%	0.02%	0.03%	0.02%	0.03%
	Total	2013	0.09%	0.09%	0.09%	0.03%	0.03%	0.03%	0.04%	0.04%	0.04%
		2014	0.09%	0.09%	0.10%	0.03%	0.03%	0.03%	0.04%	0.04%	0.04%
		2015	0.09%	0.09%	0.10%	0.03%	0.03%	0.03%	0.04%	0.04%	0.04%
		2016	0.09%	0.09%	0.10%	0.03%	0.03%	0.03%	0.04%	0.04%	0.04%
		2017	0.09%	0.09%	0.10%	0.03%	0.03%	0.03%	0.04%	0.04%	0.04%
		2018	0.09%	0.09%	0.09%	0.03%	0.03%	0.03%	0.04%	0.04%	0.04%
		2019	0.10%	0.09%	0.10%	0.03%	0.03%	0.03%	0.04%	0.04%	0.04%
Total		2013	0.51%	0.51%	0.52%	0.12%	0.12%	0.12%	0.18%	0.18%	0.18%
		2014	0.53%	0.52%	0.54%	0.12%	0.12%	0.12%	0.19%	0.19%	0.19%
		2015	0.54%	0.53%	0.55%	0.13%	0.13%	0.13%	0.20%	0.19%	0.20%
		2016	0.54%	0.53%	0.54%	0.13%	0.13%	0.14%	0.20%	0.20%	0.20%
		2017	0.54%	0.53%	0.55%	0.14%	0.14%	0.14%	0.20%	0.20%	0.20%
		2018	0.53%	0.52%	0.54%	0.14%	0.14%	0.14%	0.20%	0.20%	0.21%
		2019	0.54%	0.54%	0.55%	0.15%	0.14%	0.15%	0.21%	0.21%	0.21%

Table 10: Posterior estimates by exposure group, region and year of diagnosed HIV prevalence: posterior median and 95% CrI.

Exposure group		Year	London Median	London 95% CrI		Outside London Median	Outside London 95% CrI		England Median	England 95% CrI	
GBM	Clinic attendees	2013	10,490	10,022	10,975	10,739	10,327	11,197	21,233	20,555	21,952
		2014	12,138	11,727	12,569	11,745	11,284	12,227	23,888	23,220	24,574
		2015	12,399	12,005	12,775	12,034	11,611	12,475	24,432	23,812	25,054
		2016	12,853	12,514	13,189	12,116	11,749	12,512	24,968	24,440	25,513
		2017	13,627	13,340	13,922	12,906	12,544	13,317	26,537	26,065	27,054
		2018	13,466	13,188	13,765	13,034	12,671	13,445	26,499	26,026	27,024
		2019	13,246	12,972	13,542	14,014	13,700	14,349	27,261	26,831	27,710
	Non-clinic attendees	2013	6,266	5,695	6,820	5,926	5,352	6,452	12,188	11,330	13,024
		2014	5,581	5,058	6,090	6,146	5,539	6,721	11,724	10,865	12,544
		2015	6,157	5,653	6,687	7,290	6,711	7,863	13,444	12,643	14,271
		2016	6,076	5,614	6,554	8,333	7,782	8,890	14,418	13,658	15,182
		2017	5,619	5,196	6,052	8,374	7,799	8,942	13,992	13,253	14,717
		2018	5,463	5,021	5,892	8,907	8,323	9,477	14,367	13,618	15,093
		2019	6,365	5,913	6,819	8,502	7,963	9,046	14,865	14,141	15,604
	Total	2013	16,755	16,409	17,114	16,664	16,325	17,012	33,421	32,930	33,924
		2014	17,717	17,344	18,096	17,891	17,528	18,265	35,607	35,082	36,143
		2015	18,554	18,170	18,948	19,323	18,941	19,710	37,881	37,328	38,428
		2016	18,932	18,545	19,320	20,451	20,056	20,864	39,382	38,822	39,943
		2017	19,246	18,873	19,630	21,285	20,868	21,702	40,534	39,964	41,107
		2018	18,929	18,545	19,308	21,940	21,517	22,373	40,868	40,300	41,435
		2019	19,615	19,218	19,996	22,516	22,090	22,960	42,133	41,536	42,722
PWID		2013	704	638	781	955	875	1,054	1,661	1,554	1,782
		2014	723	656	804	994	908	1,095	1,719	1,608	1,846
		2015	731	663	811	1,027	941	1,129	1,759	1,646	1,889
		2016	686	621	763	1,039	953	1,147	1,728	1,616	1,854
		2017	692	627	769	1,028	942	1,134	1,722	1,610	1,850
		2018	648	588	722	1,052	965	1,159	1,702	1,594	1,829
		2019	654	591	730	1,053	965	1,160	1,710	1,598	1,835
Heterosexuals (Black African)	Clinic attendees	2013	4,781	4,610	4,958	6,855	6,657	7,057	11,635	11,371	11,909
		2014	4,561	4,395	4,734	6,782	6,591	6,982	11,346	11,083	11,614
		2015	4,344	4,208	4,488	6,448	6,276	6,626	10,793	10,567	11,022
		2016	4,282	4,149	4,419	6,245	6,082	6,409	10,528	10,317	10,744
		2017	4,357	4,230	4,491	6,144	5,985	6,305	10,502	10,294	10,713
		2018	4,675	4,544	4,811	6,248	6,088	6,413	10,924	10,717	11,137
		2019	4,478	4,353	4,605	6,382	6,227	6,541	10,859	10,658	11,062
	Non-clinic attendees	2013	5,122	4,836	5,410	6,111	5,779	6,433	11,232	10,781	11,679
		2014	5,569	5,284	5,856	6,517	6,189	6,848	12,086	11,656	12,536
		2015	5,893	5,624	6,160	7,354	7,029	7,665	13,246	12,821	13,658
		2016	5,881	5,611	6,150	7,888	7,571	8,209	13,771	13,349	14,201
		2017	5,847	5,586	6,113	8,284	7,967	8,608	14,134	13,712	14,560
		2018	5,421	5,162	5,684	8,584	8,254	8,919	14,006	13,584	14,428
		2019	5,852	5,598	6,125	8,839	8,511	9,181	14,694	14,269	15,126
	Total	2013	9,903	9,678	10,135	12,965	12,696	13,238	22,869	22,513	23,230
		2014	10,129	9,903	10,363	13,300	13,034	13,576	23,432	23,081	23,792
		2015	10,238	10,011	10,471	13,802	13,518	14,079	24,040	23,676	24,403
		2016	10,162	9,936	10,396	14,133	13,856	14,418	24,296	23,934	24,668
		2017	10,205	9,975	10,446	14,427	14,151	14,720	24,636	24,265	25,019
		2018	10,096	9,866	10,334	14,834	14,538	15,136	24,928	24,565	25,311
		2019	10,330	10,097	10,570	15,220	14,925	15,529	25,552	25,168	25,944
Heterosexuals (other ethnicities)	Clinic attendees	2013	2,572	2,446	2,703	5,028	4,839	5,225	7,601	7,366	7,841
		2014	2,548	2,417	2,688	5,285	5,093	5,491	7,833	7,591	8,084
		2015	2,405	2,300	2,515	5,172	4,995	5,357	7,578	7,364	7,795
		2016	2,425	2,320	2,534	5,114	4,936	5,300	7,540	7,328	7,757
		2017	2,426	2,323	2,529	5,004	4,839	5,175	7,429	7,227	7,635
		2018	2,453	2,355	2,553	5,040	4,877	5,208	7,493	7,301	7,687
		2019	2,436	2,339	2,537	5,163	5,006	5,325	7,599	7,411	7,787
	Non-clinic attendees	2013	2,714	2,522	2,912	3,669	3,401	3,937	6,383	6,045	6,725
		2014	2,930	2,723	3,139	4,005	3,728	4,282	6,938	6,582	7,299
		2015	3,193	3,008	3,383	4,671	4,392	4,943	7,863	7,524	8,198
		2016	3,223	3,041	3,417	5,253	4,970	5,532	8,479	8,128	8,825
		2017	3,299	3,116	3,489	5,715	5,436	5,988	9,015	8,664	9,359
		2018	3,170	2,985	3,354	6,143	5,849	6,421	9,311	8,962	9,653
		2019	3,418	3,238	3,611	6,408	6,124	6,703	9,829	9,476	10,182
	Total	2013	5,287	5,134	5,443	8,698	8,495	8,904	13,986	13,727	14,241
		2014	5,479	5,325	5,640	9,291	9,086	9,505	14,772	14,511	15,040
		2015	5,599	5,445	5,761	9,842	9,622	10,056	15,442	15,175	15,712
		2016	5,650	5,491	5,813	10,368	10,141	10,593	16,017	15,742	16,293
		2017	5,726	5,568	5,888	10,719	10,490	10,948	16,445	16,163	16,728
		2018	5,622	5,464	5,784	11,181	10,943	11,420	16,803	16,516	17,091
		2019	5,855	5,693	6,019	11,569	11,335	11,815	17,427	17,137	17,722
Total		2013	32,649	32,216	33,122	39,284	38,788	39,787	71,932	71,276	72,635
		2014	34,050	33,578	34,523	41,482	40,973	42,001	75,533	74,836	76,233
		2015	35,123	34,652	35,614	43,997	43,479	44,542	79,122	78,403	79,851
		2016	35,433	34,944	35,916	45,993	45,450	46,558	81,432	80,690	82,166
		2017	35,872	35,400	36,351	47,465	46,895	48,034	83,338	82,596	84,090
		2018	35,298	34,831	35,777	49,013	48,433	49,583	84,310	83,560	85,062
		2019	36,460	35,969	36,940	50,363	49,795	50,960	86,824	86,079	87,608

Table 11: Posterior estimates by exposure group, region and year of number of people living with diagnosed HIV: posterior median and 95% CrI.

			London			Outside London			England		
Exposure group		***Year***	***Median***	***95% CrI***		***Median***	***95% CrI***		***Median***	***95% CrI***	
GBM	Clinic attendees	2013	2.219%	1.239%	4.233%	1.883%	1.073%	3.393%	2.091%	1.391%	3.306%
		2014	1.901%	1.023%	3.648%	1.646%	0.931%	3.058%	1.808%	1.175%	2.937%
		2015	1.517%	0.789%	3.023%	1.386%	0.747%	2.683%	1.486%	0.937%	2.477%
		2016	1.228%	0.646%	2.418%	1.108%	0.611%	2.058%	1.195%	0.769%	1.940%
		2017	0.972%	0.496%	2.008%	0.896%	0.477%	1.757%	0.958%	0.598%	1.628%
		2018	1.010%	0.560%	1.884%	0.851%	0.449%	1.633%	0.942%	0.612%	1.514%
		2019	0.882%	0.489%	1.601%	0.625%	0.333%	1.198%	0.755%	0.485%	1.198%
	Non-clinic attendees	2013	1.531%	0.666%	3.373%	1.027%	0.424%	2.501%	1.173%	0.585%	2.418%
		2014	1.176%	0.508%	2.684%	0.959%	0.391%	2.325%	1.031%	0.505%	2.188%
		2015	0.967%	0.410%	2.240%	0.798%	0.316%	2.042%	0.855%	0.408%	1.916%
		2016	0.670%	0.273%	1.581%	0.689%	0.281%	1.700%	0.699%	0.337%	1.525%
		2017	0.469%	0.189%	1.159%	0.612%	0.247%	1.473%	0.591%	0.275%	1.299%
		2018	0.469%	0.202%	1.078%	0.572%	0.235%	1.377%	0.562%	0.268%	1.214%
		2019	0.466%	0.204%	1.041%	0.417%	0.165%	1.034%	0.438%	0.210%	0.944%
	Total	2013	1.812%	0.934%	3.544%	1.169%	0.549%	2.569%	1.386%	0.796%	2.533%
		2014	1.509%	0.778%	2.977%	1.091%	0.515%	2.414%	1.242%	0.711%	2.289%
		2015	1.229%	0.614%	2.477%	0.917%	0.422%	2.135%	1.032%	0.583%	1.982%
		2016	0.934%	0.468%	1.900%	0.777%	0.362%	1.728%	0.839%	0.479%	1.587%
		2017	0.713%	0.349%	1.515%	0.677%	0.315%	1.503%	0.704%	0.390%	1.352%
		2018	0.724%	0.386%	1.410%	0.642%	0.299%	1.395%	0.680%	0.390%	1.263%
		2019	0.670%	0.355%	1.251%	0.472%	0.218%	1.047%	0.542%	0.311%	0.983%
PWID		2013	0.233%	0.068%	0.634%	0.047%	0.014%	0.126%	0.071%	0.021%	0.186%
		2014	0.343%	0.101%	0.891%	0.073%	0.022%	0.191%	0.107%	0.033%	0.274%
		2015	0.647%	0.198%	1.578%	0.145%	0.044%	0.362%	0.211%	0.066%	0.504%
		2016	0.616%	0.192%	1.526%	0.145%	0.045%	0.359%	0.206%	0.067%	0.497%
		2017	0.437%	0.122%	1.206%	0.101%	0.030%	0.268%	0.145%	0.044%	0.377%
		2018	0.224%	0.033%	0.774%	0.057%	0.008%	0.195%	0.078%	0.012%	0.265%
		2019	0.142%	0.016%	0.591%	0.035%	0.004%	0.150%	0.049%	0.006%	0.203%
Heterosexuals (Black African)	Clinic attendees	2013	1.524%	1.178%	1.959%	1.833%	1.394%	2.409%	1.655%	1.344%	2.027%
		2014	1.225%	0.941%	1.589%	2.046%	1.579%	2.630%	1.578%	1.286%	1.917%
		2015	0.789%	0.607%	1.023%	1.651%	1.267%	2.146%	1.165%	0.947%	1.427%
		2016	0.695%	0.529%	0.900%	1.167%	0.904%	1.487%	0.912%	0.751%	1.100%
		2017	0.683%	0.525%	0.883%	1.116%	0.853%	1.445%	0.894%	0.732%	1.082%
		2018	0.588%	0.446%	0.774%	1.019%	0.790%	1.306%	0.806%	0.666%	0.974%
		2019	0.529%	0.407%	0.687%	0.796%	0.615%	1.020%	0.669%	0.554%	0.804%
	Non-clinic attendees	2013	0.155%	0.070%	0.289%	0.149%	0.064%	0.277%	0.153%	0.072%	0.269%
		2014	0.119%	0.054%	0.232%	0.186%	0.085%	0.342%	0.148%	0.071%	0.262%
		2015	0.162%	0.096%	0.265%	0.277%	0.157%	0.453%	0.211%	0.129%	0.323%
		2016	0.078%	0.037%	0.150%	0.130%	0.061%	0.240%	0.100%	0.050%	0.176%
		2017	0.094%	0.048%	0.177%	0.179%	0.099%	0.304%	0.129%	0.073%	0.214%
		2018	0.054%	0.026%	0.103%	0.146%	0.078%	0.248%	0.091%	0.051%	0.150%
		2019	0.065%	0.033%	0.120%	0.148%	0.081%	0.258%	0.098%	0.057%	0.163%
	Total	2013	0.313%	0.236%	0.433%	0.339%	0.255%	0.465%	0.325%	0.258%	0.422%
		2014	0.247%	0.184%	0.351%	0.411%	0.315%	0.555%	0.316%	0.251%	0.413%
		2015	0.233%	0.172%	0.327%	0.450%	0.340%	0.608%	0.324%	0.253%	0.419%
		2016	0.146%	0.105%	0.214%	0.268%	0.198%	0.370%	0.196%	0.151%	0.263%
		2017	0.157%	0.111%	0.233%	0.315%	0.237%	0.425%	0.222%	0.173%	0.294%
		2018	0.110%	0.079%	0.159%	0.281%	0.213%	0.375%	0.180%	0.142%	0.235%
		2019	0.113%	0.080%	0.167%	0.255%	0.192%	0.351%	0.171%	0.133%	0.229%
Heterosexuals (other ethnicities)	Clinic attendees	2013	0.139%	0.104%	0.189%	0.105%	0.081%	0.136%	0.115%	0.093%	0.141%
		2014	0.136%	0.101%	0.188%	0.111%	0.088%	0.143%	0.118%	0.097%	0.145%
		2015	0.086%	0.062%	0.123%	0.093%	0.074%	0.118%	0.091%	0.075%	0.113%
		2016	0.084%	0.062%	0.120%	0.093%	0.073%	0.120%	0.091%	0.074%	0.112%
		2017	0.081%	0.061%	0.110%	0.072%	0.056%	0.093%	0.074%	0.061%	0.092%
		2018	0.093%	0.071%	0.124%	0.062%	0.049%	0.080%	0.069%	0.057%	0.085%
		2019	0.087%	0.066%	0.116%	0.058%	0.046%	0.075%	0.065%	0.054%	0.079%
	Non-clinic attendees	2013	0.005%	0.001%	0.014%	0.001%	0.000%	0.004%	0.002%	0.001%	0.005%
		2014	0.005%	0.002%	0.016%	0.001%	0.000%	0.005%	0.002%	0.001%	0.006%
		2015	0.006%	0.002%	0.016%	0.002%	0.001%	0.006%	0.003%	0.001%	0.007%
		2016	0.004%	0.002%	0.013%	0.002%	0.001%	0.006%	0.002%	0.001%	0.007%
		2017	0.003%	0.001%	0.011%	0.002%	0.001%	0.006%	0.002%	0.001%	0.007%
		2018	0.003%	0.001%	0.011%	0.002%	0.001%	0.006%	0.002%	0.001%	0.006%
		2019	0.004%	0.001%	0.012%	0.002%	0.001%	0.005%	0.002%	0.001%	0.006%
	Total	2013	0.012%	0.008%	0.021%	0.004%	0.003%	0.007%	0.005%	0.004%	0.009%
		2014	0.012%	0.008%	0.023%	0.004%	0.003%	0.008%	0.006%	0.004%	0.010%
		2015	0.010%	0.006%	0.021%	0.005%	0.003%	0.009%	0.005%	0.004%	0.010%
		2016	0.009%	0.006%	0.018%	0.005%	0.003%	0.009%	0.005%	0.004%	0.010%
		2017	0.007%	0.005%	0.015%	0.004%	0.003%	0.008%	0.005%	0.003%	0.009%
		2018	0.008%	0.005%	0.015%	0.003%	0.002%	0.007%	0.004%	0.003%	0.008%
		2019	0.007%	0.005%	0.015%	0.003%	0.002%	0.007%	0.004%	0.003%	0.008%
Total		2013	0.074%	0.052%	0.116%	0.020%	0.013%	0.036%	0.029%	0.021%	0.044%
		2014	0.064%	0.045%	0.100%	0.020%	0.013%	0.035%	0.027%	0.020%	0.042%
		2015	0.055%	0.038%	0.086%	0.019%	0.013%	0.033%	0.025%	0.018%	0.038%
		2016	0.041%	0.029%	0.064%	0.016%	0.011%	0.027%	0.020%	0.015%	0.031%
		2017	0.035%	0.025%	0.055%	0.015%	0.010%	0.024%	0.018%	0.013%	0.027%
		2018	0.032%	0.022%	0.049%	0.013%	0.009%	0.022%	0.016%	0.012%	0.025%
		2019	0.031%	0.022%	0.046%	0.011%	0.008%	0.018%	0.014%	0.011%	0.021%

Table 12: Posterior estimates by exposure group, region and year of undiagnosed HIV prevalence: posterior median and 95% CrI.

Exposure group		Year	London Median	London 95% CrI		Outside London Median	Outside London 95% CrI		England Median	England 95% CrI	
GBM	Clinic attendees	2013	1,237	690	2,358	1,118	637	2,011	2,406	1,604	3,800
		2014	1,244	671	2,388	1,103	623	2,047	2,394	1,557	3,885
		2015	1,039	541	2,071	999	538	1,934	2,089	1,318	3,481
		2016	857	450	1,685	819	452	1,523	1,716	1,105	2,788
		2017	703	359	1,450	733	390	1,440	1,476	921	2,507
		2018	719	398	1,344	739	391	1,420	1,489	968	2,395
		2019	645	357	1,170	593	315	1,137	1,269	815	2,012
	Non-clinic attendees	2013	1,371	585	3,166	3,149	1,275	7,808	4,647	2,269	9,707
		2014	964	403	2,267	2,886	1,148	7,103	3,952	1,906	8,483
		2015	788	317	1,899	2,378	924	6,255	3,252	1,516	7,377
		2016	550	219	1,333	2,067	829	5,195	2,672	1,269	5,964
		2017	375	148	972	1,792	710	4,414	2,207	1,005	4,971
		2018	391	158	940	1,660	660	4,070	2,093	977	4,625
		2019	382	160	880	1,173	449	3,016	1,597	742	3,539
	Total	2013	2,633	1,356	5,259	4,280	2,017	9,545	7,093	4,021	13,188
		2014	2,231	1,148	4,454	4,025	1,875	8,929	6,406	3,656	12,022
		2015	1,846	907	3,789	3,406	1,541	7,946	5,384	3,011	10,453
		2016	1,418	711	2,895	2,900	1,333	6,558	4,409	2,530	8,448
		2017	1,089	532	2,308	2,540	1,165	5,684	3,715	2,059	7,200
		2018	1,122	590	2,188	2,418	1,113	5,302	3,606	2,059	6,761
		2019	1,040	550	1,947	1,778	814	4,024	2,885	1,649	5,302
PWID		2013	31	9	83	43	13	115	75	23	194
		2014	46	14	119	67	20	175	114	35	287
		2015	87	27	211	134	42	333	224	71	532
		2016	84	26	206	134	43	331	220	71	528
		2017	60	17	162	95	27	251	155	47	405
		2018	31	5	106	53	8	179	84	13	280
		2019	19	2	80	33	4	139	53	6	217
Heterosexuals (Black African)	Clinic attendees	2013	703	543	904	583	443	767	1,291	1,048	1,580
		2014	574	442	745	696	538	895	1,277	1,041	1,552
		2015	367	282	476	583	447	759	953	776	1,168
		2016	315	240	408	436	337	555	754	622	909
		2017	300	230	387	453	347	585	755	618	914
		2018	258	195	339	445	345	571	704	582	853
		2019	230	177	299	370	286	475	602	498	723
	Non-clinic attendees	2013	552	249	1,034	374	162	697	933	436	1,637
		2014	430	195	836	463	214	854	903	432	1,596
		2015	594	351	972	691	392	1,129	1,296	790	1,986
		2016	291	138	555	321	152	594	619	311	1,088
		2017	352	179	660	436	241	741	797	451	1,321
		2018	203	97	389	351	187	598	560	312	930
		2019	246	124	455	354	194	615	604	348	1,006
	Total	2013	1,261	952	1,744	961	722	1,319	2,231	1,772	2,895
		2014	1,008	752	1,431	1,166	891	1,578	2,186	1,732	2,855
		2015	963	709	1,348	1,279	965	1,729	2,255	1,767	2,922
		2016	609	437	892	763	565	1,054	1,378	1,060	1,846
		2017	655	464	975	895	673	1,211	1,555	1,213	2,068
		2018	463	331	671	800	608	1,069	1,270	998	1,657
		2019	477	338	706	727	547	999	1,209	941	1,617
Heterosexuals (other ethnicities)	Clinic attendees	2013	437	327	592	864	669	1,124	1,305	1,065	1,611
		2014	434	324	603	1,002	787	1,284	1,442	1,187	1,772
		2015	290	209	414	853	676	1,088	1,149	947	1,416
		2016	286	212	410	875	682	1,125	1,167	950	1,437
		2017	255	192	345	693	544	891	951	781	1,170
		2018	262	199	349	602	473	780	868	716	1,060
		2019	229	175	305	563	441	724	796	659	968
	Non-clinic attendees	2013	248	80	777	438	143	1,249	696	244	1,957
		2014	287	91	895	468	150	1,490	765	261	2,264
		2015	324	113	910	644	219	1,943	979	361	2,745
		2016	231	86	751	684	293	1,954	924	397	2,593
		2017	190	60	643	650	212	2,095	850	286	2,682
		2018	192	63	657	563	209	1,850	765	292	2,427
		2019	225	81	703	608	235	1,663	844	334	2,293
	Total	2013	694	481	1,230	1,313	952	2,173	2,016	1,515	3,308
		2014	733	492	1,348	1,486	1,080	2,521	2,224	1,661	3,742
		2015	621	385	1,222	1,501	1,029	2,851	2,137	1,483	3,940
		2016	528	344	1,066	1,565	1,127	2,866	2,104	1,539	3,771
		2017	450	292	909	1,346	877	2,829	1,808	1,224	3,644
		2018	459	301	926	1,175	785	2,479	1,640	1,135	3,293
		2019	459	296	942	1,178	786	2,243	1,644	1,125	3,088
Total		2013	4,691	3,289	7,356	6,698	4,311	12,036	11,568	8,347	17,683
		2014	4,101	2,871	6,429	6,840	4,543	11,886	11,067	8,087	16,961
		2015	3,597	2,509	5,620	6,474	4,363	11,201	10,218	7,502	15,431
		2016	2,713	1,895	4,241	5,504	3,706	9,332	8,299	6,113	12,565
		2017	2,330	1,645	3,638	5,025	3,388	8,382	7,430	5,410	11,210
		2018	2,136	1,497	3,270	4,578	3,047	7,662	6,786	4,926	10,135
		2019	2,052	1,452	3,080	3,822	2,648	6,232	5,927	4,427	8,706

Table 13: Posterior estimates by exposure group, region and year of number of people living with undiagnosed HIV: posterior median and 95% CrI.

			London			Outside London			England		
Exposure group		*Year*	*Median*	*95% CrI*		*Median*	*95% CrI*		*Median*	*95% CrI*	
GBM	Clinic attendees	2013	89%	82%	94%	91%	84%	94%	90%	85%	93%
		2014	91%	84%	95%	91%	85%	95%	91%	86%	94%
		2015	92%	86%	96%	92%	86%	96%	92%	88%	95%
		2016	94%	88%	97%	94%	89%	96%	94%	90%	96%
		2017	95%	90%	97%	95%	90%	97%	95%	91%	97%
		2018	95%	91%	97%	95%	90%	97%	95%	92%	96%
		2019	95%	92%	97%	96%	93%	98%	96%	93%	97%
	Non-clinic attendees	2013	82%	66%	91%	65%	43%	82%	72%	55%	84%
		2014	85%	71%	93%	68%	46%	84%	75%	58%	86%
		2015	89%	76%	95%	75%	54%	89%	80%	64%	90%
		2016	92%	82%	97%	80%	62%	91%	84%	71%	92%
		2017	94%	85%	97%	82%	66%	92%	86%	74%	93%
		2018	93%	85%	97%	84%	69%	93%	87%	76%	94%
		2019	94%	88%	98%	88%	74%	95%	90%	81%	95%
	Total	2013	86%	76%	93%	80%	64%	89%	82%	72%	89%
		2014	89%	80%	94%	82%	67%	91%	85%	75%	91%
		2015	91%	83%	95%	85%	71%	93%	88%	78%	93%
		2016	93%	87%	96%	88%	76%	94%	90%	82%	94%
		2017	95%	89%	97%	89%	79%	95%	92%	85%	95%
		2018	94%	90%	97%	90%	80%	95%	92%	86%	95%
		2019	95%	91%	97%	93%	85%	97%	94%	89%	96%
PWID		2013	96%	89%	99%	96%	89%	99%	96%	90%	99%
		2014	94%	86%	98%	94%	85%	98%	94%	86%	98%
		2015	89%	78%	96%	88%	75%	96%	89%	77%	96%
		2016	89%	77%	96%	89%	76%	96%	89%	77%	96%
		2017	92%	81%	98%	92%	80%	97%	92%	81%	97%
		2018	95%	86%	99%	95%	85%	99%	95%	86%	99%
		2019	97%	89%	100%	97%	88%	100%	97%	89%	100%
Heterosexuals (Black African)	Clinic attendees	2013	87%	84%	90%	92%	90%	94%	90%	88%	92%
		2014	89%	86%	91%	91%	88%	93%	90%	88%	92%
		2015	92%	90%	94%	92%	90%	94%	92%	90%	93%
		2016	93%	91%	95%	93%	92%	95%	93%	92%	94%
		2017	94%	92%	95%	93%	91%	95%	93%	92%	94%
		2018	95%	93%	96%	93%	92%	95%	94%	93%	95%
		2019	95%	94%	96%	95%	93%	96%	95%	94%	96%
	Non-clinic attendees	2013	90%	83%	95%	94%	90%	97%	92%	87%	96%
		2014	93%	87%	97%	93%	88%	97%	93%	88%	97%
		2015	91%	86%	94%	91%	87%	95%	91%	87%	94%
		2016	95%	91%	98%	96%	93%	98%	96%	93%	98%
		2017	94%	90%	97%	95%	92%	97%	95%	91%	97%
		2018	96%	93%	98%	96%	93%	98%	96%	94%	98%
		2019	96%	93%	98%	96%	93%	98%	96%	94%	98%
	Total	2013	89%	85%	91%	93%	91%	95%	91%	89%	93%
		2014	91%	88%	93%	92%	89%	94%	91%	89%	93%
		2015	91%	88%	94%	92%	89%	93%	91%	89%	93%
		2016	94%	92%	96%	95%	93%	96%	95%	93%	96%
		2017	94%	91%	96%	94%	92%	96%	94%	92%	95%
		2018	96%	94%	97%	95%	93%	96%	95%	94%	96%
		2019	96%	94%	97%	95%	94%	97%	95%	94%	96%
Heterosexuals (other ethnicities)	Clinic attendees	2013	85%	81%	89%	85%	82%	88%	85%	82%	88%
		2014	85%	81%	89%	84%	80%	87%	84%	82%	87%
		2015	89%	85%	92%	86%	83%	88%	87%	84%	89%
		2016	89%	86%	92%	85%	82%	88%	87%	84%	89%
		2017	90%	88%	93%	88%	85%	90%	89%	86%	90%
		2018	90%	88%	93%	89%	87%	91%	90%	88%	91%
		2019	91%	89%	93%	90%	88%	92%	91%	89%	92%
	Non-clinic attendees	2013	92%	78%	97%	89%	75%	96%	90%	77%	96%
		2014	91%	77%	97%	90%	73%	96%	90%	75%	96%
		2015	91%	78%	97%	88%	70%	96%	89%	74%	96%
		2016	93%	81%	97%	89%	73%	95%	90%	76%	96%
		2017	95%	84%	98%	90%	73%	96%	91%	77%	97%
		2018	94%	83%	98%	92%	77%	97%	92%	79%	97%
		2019	94%	83%	98%	91%	79%	96%	92%	81%	97%
	Total	2013	88%	81%	92%	87%	80%	90%	87%	81%	90%
		2014	88%	80%	92%	86%	79%	90%	87%	80%	90%
		2015	90%	82%	94%	87%	78%	91%	88%	80%	91%
		2016	91%	84%	94%	87%	78%	90%	88%	81%	91%
		2017	93%	86%	95%	89%	79%	92%	90%	82%	93%
		2018	92%	86%	95%	90%	82%	93%	91%	84%	94%
		2019	93%	86%	95%	91%	84%	94%	91%	85%	94%
Total		2013	87%	82%	91%	85%	77%	90%	86%	80%	90%
		2014	89%	84%	92%	86%	78%	90%	87%	82%	90%
		2015	91%	86%	93%	87%	80%	91%	89%	84%	91%
		2016	93%	89%	95%	89%	83%	93%	91%	87%	93%
		2017	94%	91%	96%	90%	85%	93%	92%	88%	94%
		2018	94%	92%	96%	91%	86%	94%	93%	89%	94%
		2019	95%	92%	96%	93%	89%	95%	94%	91%	95%

Table 14: Posterior estimates by exposure group, region and year of proportion of people living with diagnosed HIV: posterior median and 95% CrI.

Exposure group		*Year*	**London**			**Outside London**			**England**		
			Median	*95% CrI*		*Median*	*95% CrI*		*Median*	*95% CrI*	
GBM	Clinic attendees	2013	11%	6%	18%	9%	6%	16%	10%	7%	15%
		2014	9%	5%	16%	9%	5%	15%	9%	6%	14%
		2015	8%	4%	14%	8%	4%	14%	8%	5%	12%
		2016	6%	3%	12%	6%	4%	11%	6%	4%	10%
		2017	5%	3%	10%	5%	3%	10%	5%	3%	9%
		2018	5%	3%	9%	5%	3%	10%	5%	4%	8%
		2019	5%	3%	8%	4%	2%	7%	4%	3%	7%
	Non-clinic attendees	2013	18%	9%	34%	35%	18%	57%	28%	16%	45%
		2014	15%	7%	29%	32%	16%	54%	25%	14%	42%
		2015	11%	5%	24%	25%	11%	46%	20%	10%	36%
		2016	8%	3%	18%	20%	9%	38%	16%	8%	29%
		2017	6%	3%	15%	18%	8%	34%	14%	7%	26%
		2018	7%	3%	15%	16%	7%	31%	13%	6%	24%
		2019	6%	2%	12%	12%	5%	26%	10%	5%	19%
	Total	2013	14%	7%	24%	20%	11%	36%	18%	11%	28%
		2014	11%	6%	20%	18%	9%	33%	15%	9%	25%
		2015	9%	5%	17%	15%	7%	29%	12%	7%	22%
		2016	7%	4%	13%	12%	6%	24%	10%	6%	18%
		2017	5%	3%	11%	11%	5%	21%	8%	5%	15%
		2018	6%	3%	10%	10%	5%	20%	8%	5%	14%
		2019	5%	3%	9%	7%	3%	15%	6%	4%	11%
PWID		2013	4%	1%	11%	4%	1%	11%	4%	1%	10%
		2014	6%	2%	14%	6%	2%	15%	6%	2%	14%
		2015	11%	4%	22%	12%	4%	25%	11%	4%	23%
		2016	11%	4%	23%	11%	4%	24%	11%	4%	23%
		2017	8%	2%	19%	8%	3%	20%	8%	3%	19%
		2018	5%	1%	14%	5%	1%	15%	5%	1%	14%
		2019	3%	0%	11%	3%	0%	12%	3%	0%	11%
Heterosexuals (Black African)	Clinic attendees	2013	13%	10%	16%	8%	6%	10%	10%	8%	12%
		2014	11%	9%	14%	9%	7%	12%	10%	8%	12%
		2015	8%	6%	10%	8%	6%	10%	8%	7%	10%
		2016	7%	5%	9%	7%	5%	8%	7%	6%	8%
		2017	6%	5%	8%	7%	5%	9%	7%	6%	8%
		2018	5%	4%	7%	7%	5%	8%	6%	5%	7%
		2019	5%	4%	6%	5%	4%	7%	5%	4%	6%
	Non-clinic attendees	2013	10%	5%	17%	6%	3%	10%	8%	4%	13%
		2014	7%	3%	13%	7%	3%	12%	7%	3%	12%
		2015	9%	6%	14%	9%	5%	13%	9%	6%	13%
		2016	5%	2%	9%	4%	2%	7%	4%	2%	7%
		2017	6%	3%	10%	5%	3%	8%	5%	3%	9%
		2018	4%	2%	7%	4%	2%	7%	4%	2%	6%
		2019	4%	2%	7%	4%	2%	7%	4%	2%	6%
	Total	2013	11%	9%	15%	7%	5%	9%	9%	7%	11%
		2014	9%	7%	12%	8%	6%	11%	9%	7%	11%
		2015	9%	6%	12%	8%	7%	11%	9%	7%	11%
		2016	6%	4%	8%	5%	4%	7%	5%	4%	7%
		2017	6%	4%	9%	6%	4%	8%	6%	5%	8%
		2018	4%	3%	6%	5%	4%	7%	5%	4%	6%
		2019	4%	3%	6%	5%	3%	6%	5%	4%	6%
Heterosexuals (other ethnicities)	Clinic attendees	2013	15%	11%	19%	15%	12%	18%	15%	12%	18%
		2014	15%	11%	19%	16%	13%	20%	16%	13%	18%
		2015	11%	8%	15%	14%	12%	17%	13%	11%	16%
		2016	11%	8%	14%	15%	12%	18%	13%	11%	16%
		2017	10%	7%	12%	12%	10%	15%	11%	10%	14%
		2018	10%	7%	12%	11%	9%	13%	10%	9%	12%
		2019	9%	7%	11%	10%	8%	12%	9%	8%	11%
	Non-clinic attendees	2013	8%	3%	22%	11%	4%	25%	10%	4%	23%
		2014	9%	3%	23%	10%	4%	27%	10%	4%	25%
		2015	9%	3%	22%	12%	4%	30%	11%	4%	26%
		2016	7%	3%	19%	11%	5%	27%	10%	4%	24%
		2017	5%	2%	16%	10%	4%	27%	9%	3%	23%
		2018	6%	2%	17%	8%	3%	23%	8%	3%	21%
		2019	6%	2%	17%	9%	4%	21%	8%	3%	19%
	Total	2013	12%	8%	19%	13%	10%	20%	13%	10%	19%
		2014	12%	8%	20%	14%	10%	21%	13%	10%	20%
		2015	10%	6%	18%	13%	9%	22%	12%	9%	20%
		2016	9%	6%	16%	13%	10%	22%	12%	9%	19%
		2017	7%	5%	14%	11%	8%	21%	10%	7%	18%
		2018	8%	5%	14%	10%	7%	18%	9%	6%	16%
		2019	7%	5%	14%	9%	6%	16%	9%	6%	15%
Total		2013	13%	9%	18%	15%	10%	23%	14%	10%	20%
		2014	11%	8%	16%	14%	10%	22%	13%	10%	18%
		2015	9%	7%	14%	13%	9%	20%	11%	9%	16%
		2016	7%	5%	11%	11%	7%	17%	9%	7%	13%
		2017	6%	4%	9%	10%	7%	15%	8%	6%	12%
		2018	6%	4%	8%	9%	6%	14%	7%	6%	11%
		2019	5%	4%	8%	7%	5%	11%	6%	5%	9%

Table 15: Posterior estimates by exposure group, region and year of proportion of people living with undiagnosed HIV: posterior median and 95% CrI.

5 Model assessment

5.1 Selected data and deviance summaries

Deviance summaries (posterior mean deviance $\overline{D}$, deviance evaluated at posterior mean of parameters ($D(\overline{\psi})$), effective number of parameters p_D, and deviance information criterion (DIC)) are given in Tables 16 to 22 for selected key data sources: HIV prevalence and proportion diagnosed in PWID attending services participating in the UAM; HARS numbers diagnosed; GUMCAD numbers attending, testing for HIV and being diagnosed; NSHPC women previously undiagnosed and newly diagnosed in current pregnancy).

Age	Sex	Region	Year	Observed prevalence			Posterior summaries					Deviance summaries			
				Positive	Tested	Proportion	Mean	SD	Median	95% CrI		$\overline{D}$	$D(\overline{\psi})$	p_D	DIC
15-34	Men	London	2013	2	58	0.0345	0.0127	0.0021	0.0126	0.0085	0.0169	1.56	0.00	1.56	3.12
			2014	2	50	0.0400	0.0109	0.0022	0.0108	0.0069	0.0152	2.42	0.00	2.42	4.84
			2015	0	43	0.0000	0.0110	0.0021	0.0109	0.0071	0.0152	0.95	0.00	0.95	1.90
			2016	0	10	0.0000	0.0088	0.0018	0.0087	0.0053	0.0125	0.18	0.00	0.18	0.35
			2017	2	22	0.0909	0.0074	0.0015	0.0074	0.0046	0.0105	6.60	0.00	6.60	13.20
			2018	2	36	0.0556	0.0075	0.0012	0.0074	0.0053	0.0101	4.69	0.00	4.69	9.38
			2019	2	39	0.0513	0.0060	0.0011	0.0060	0.0041	0.0083	5.19	0.00	5.19	10.38
		Outside London	2013	6	723	0.0083	0.0023	0.0004	0.0023	0.0015	0.0031	7.00	0.00	7.00	14.00
			2014	3	592	0.0051	0.0020	0.0004	0.0019	0.0012	0.0028	2.17	0.00	2.17	4.33
			2015	7	435	0.0161	0.0020	0.0004	0.0020	0.0013	0.0027	17.45	0.00	17.45	34.91
			2016	2	418	0.0048	0.0016	0.0003	0.0016	0.0010	0.0022	1.85	0.00	1.85	3.71
			2017	1	318	0.0031	0.0013	0.0003	0.0013	0.0008	0.0019	0.62	0.00	0.62	1.23
			2018	1	302	0.0033	0.0013	0.0002	0.0013	0.0009	0.0018	0.65	0.00	0.65	1.29
			2019	1	351	0.0028	0.0011	0.0002	0.0011	0.0007	0.0015	0.74	0.00	0.74	1.48
	Women	London	2013	0	26	0.0000	0.0102	0.0025	0.0100	0.0060	0.0158	0.53	0.00	0.53	1.07
			2014	0	33	0.0000	0.0088	0.0023	0.0086	0.0049	0.0138	0.58	0.00	0.58	1.16
			2015	1	27	0.0370	0.0089	0.0023	0.0086	0.0050	0.0140	1.43	0.00	1.43	2.86
			2016	1	14	0.0714	0.0071	0.0019	0.0069	0.0039	0.0113	2.96	0.00	2.96	5.91
			2017	0	13	0.0000	0.0060	0.0016	0.0058	0.0033	0.0096	0.16	0.00	0.16	0.31
			2018	0	22	0.0000	0.0060	0.0014	0.0059	0.0036	0.0092	0.27	0.00	0.27	0.53
			2019	0	24	0.0000	0.0048	0.0012	0.0047	0.0029	0.0075	0.23	0.00	0.23	0.47
		Outside London	2013	1	319	0.0031	0.0018	0.0005	0.0018	0.0011	0.0029	0.31	0.00	0.31	0.61
			2014	0	291	0.0000	0.0016	0.0004	0.0015	0.0009	0.0026	0.92	0.00	0.92	1.84
			2015	1	231	0.0043	0.0016	0.0004	0.0015	0.0009	0.0025	0.81	0.00	0.81	1.62
			2016	3	226	0.0133	0.0013	0.0004	0.0012	0.0007	0.0021	8.92	0.00	8.92	17.85
			2017	0	203	0.0000	0.0011	0.0003	0.0010	0.0006	0.0018	0.43	0.00	0.43	0.87
			2018	2	173	0.0116	0.0011	0.0003	0.0011	0.0006	0.0017	5.99	0.00	5.99	11.98
			2019	1	221	0.0045	0.0009	0.0002	0.0008	0.0005	0.0014	1.76	0.00	1.76	3.51
35-44	Men	London	2013	9	104	0.0865	0.0443	0.0085	0.0437	0.0290	0.0623	3.81	0.00	3.81	7.62
			2014	7	81	0.0864	0.0381	0.0083	0.0375	0.0236	0.0561	4.18	0.00	4.18	8.35
			2015	1	69	0.0145	0.0385	0.0084	0.0378	0.0239	0.0568	1.46	0.00	1.46	2.91
			2016	0	50	0.0000	0.0310	0.0074	0.0305	0.0182	0.0473	3.16	0.00	3.16	6.31
			2017	1	55	0.0182	0.0263	0.0063	0.0257	0.0153	0.0402	0.22	0.00	0.22	0.44
			2018	5	101	0.0495	0.0266	0.0056	0.0261	0.0171	0.0389	1.86	0.00	1.86	3.71
			2019	3	102	0.0294	0.0214	0.0048	0.0211	0.0133	0.0320	0.43	0.00	0.43	0.85
		Outside London	2013	2	725	0.0028	0.0082	0.0016	0.0081	0.0053	0.0115	3.62	0.00	3.62	7.23
			2014	3	724	0.0041	0.0070	0.0015	0.0069	0.0043	0.0104	1.15	0.00	1.15	2.29
			2015	4	649	0.0062	0.0071	0.0015	0.0070	0.0045	0.0102	0.26	0.00	0.26	0.51
			2016	3	651	0.0046	0.0057	0.0013	0.0056	0.0034	0.0085	0.30	0.00	0.30	0.60
			2017	1	545	0.0018	0.0048	0.0011	0.0047	0.0028	0.0073	1.36	0.00	1.36	2.72
			2018	2	570	0.0035	0.0048	0.0010	0.0048	0.0031	0.0070	0.32	0.00	0.32	0.64
			2019	2	729	0.0027	0.0039	0.0009	0.0038	0.0024	0.0057	0.37	0.00	0.37	0.74
	Women	London	2013	2	41	0.0488	0.0357	0.0084	0.0350	0.0215	0.0542	0.30	0.00	0.30	0.59
			2014	1	48	0.0208	0.0307	0.0078	0.0300	0.0177	0.0481	0.24	0.00	0.24	0.49
			2015	3	34	0.0882	0.0310	0.0080	0.0303	0.0178	0.0492	2.70	0.00	2.70	5.39
			2016	1	33	0.0303	0.0250	0.0068	0.0243	0.0138	0.0402	0.11	0.00	0.11	0.22
			2017	2	33	0.0606	0.0211	0.0058	0.0205	0.0116	0.0343	1.82	0.00	1.82	3.63
			2018	1	40	0.0250	0.0214	0.0053	0.0208	0.0126	0.0333	0.09	0.00	0.09	0.17
			2019	1	40	0.0250	0.0172	0.0044	0.0167	0.0099	0.0274	0.19	0.00	0.19	0.38
		Outside London	2013	1	229	0.0044	0.0066	0.0016	0.0064	0.0039	0.0101	0.25	0.00	0.25	0.50
			2014	0	217	0.0000	0.0056	0.0015	0.0055	0.0031	0.0089	2.44	0.00	2.44	4.89
			2015	0	183	0.0000	0.0057	0.0014	0.0055	0.0033	0.0089	2.08	0.00	2.08	4.16
			2016	0	237	0.0000	0.0045	0.0012	0.0044	0.0025	0.0073	2.15	0.00	2.15	4.31
			2017	2	223	0.0090	0.0038	0.0011	0.0037	0.0021	0.0062	1.27	0.00	1.27	2.54
			2018	1	232	0.0043	0.0039	0.0010	0.0038	0.0023	0.0061	0.07	0.00	0.07	0.15
			2019	1	306	0.0033	0.0031	0.0008	0.0030	0.0018	0.0049	0.07	0.00	0.07	0.14
45-59	Men	London	2013	3	91	0.0330	0.0549	0.0100	0.0542	0.0371	0.0763	1.09	0.00	1.09	2.18
			2014	5	117	0.0427	0.0473	0.0098	0.0469	0.0301	0.0683	0.29	0.00	0.29	0.57
			2015	2	111	0.0180	0.0478	0.0097	0.0471	0.0305	0.0685	2.90	0.00	2.90	5.81
			2016	2	78	0.0256	0.0386	0.0087	0.0380	0.0233	0.0575	0.51	0.00	0.51	1.02
			2017	5	98	0.0510	0.0327	0.0074	0.0322	0.0198	0.0486	1.16	0.00	1.16	2.31
			2018	8	161	0.0497	0.0331	0.0065	0.0325	0.0220	0.0471	1.51	0.00	1.51	3.01
			2019	9	160	0.0563	0.0267	0.0055	0.0263	0.0172	0.0385	4.49	0.00	4.49	8.98
		Outside London	2013	2	282	0.0071	0.0103	0.0021	0.0101	0.0066	0.0148	0.40	0.00	0.40	0.80
			2014	2	332	0.0060	0.0088	0.0020	0.0087	0.0054	0.0130	0.43	0.00	0.43	0.86
			2015	4	337	0.0119	0.0089	0.0019	0.0087	0.0056	0.0128	0.49	0.00	0.49	0.98
			2016	4	357	0.0112	0.0071	0.0017	0.0070	0.0042	0.0107	0.95	0.00	0.95	1.89
			2017	2	336	0.0060	0.0060	0.0014	0.0059	0.0035	0.0091	0.12	0.00	0.12	0.23
			2018	3	375	0.0080	0.0061	0.0013	0.0060	0.0039	0.0089	0.34	0.00	0.34	0.68
			2019	2	559	0.0036	0.0049	0.0011	0.0048	0.0030	0.0072	0.31	0.00	0.31	0.62
	Women	London	2013	0	38	0.0000	0.0444	0.0102	0.0435	0.0271	0.0672	3.46	0.00	3.46	6.91
			2014	1	39	0.0256	0.0382	0.0095	0.0375	0.0221	0.0587	0.25	0.00	0.25	0.51
			2015	0	35	0.0000	0.0386	0.0096	0.0377	0.0225	0.0600	2.76	0.00	2.76	5.52
			2016	1	30	0.0333	0.0311	0.0083	0.0304	0.0175	0.0498	0.08	0.00	0.08	0.16
			2017	0	39	0.0000	0.0263	0.0071	0.0256	0.0147	0.0421	2.08	0.00	2.08	4.17
			2018	1	31	0.0323	0.0267	0.0064	0.0261	0.0160	0.0408	0.09	0.00	0.09	0.19
			2019	1	59	0.0169	0.0215	0.0053	0.0209	0.0127	0.0333	0.12	0.00	0.12	0.25
		Outside London	2013	1	59	0.0169	0.0083	0.0021	0.0080	0.0047	0.0130	0.48	0.00	0.48	0.96
			2014	0	65	0.0000	0.0071	0.0019	0.0069	0.0039	0.0114	0.92	0.00	0.92	1.84
			2015	0	78	0.0000	0.0071	0.0019	0.0070	0.0041	0.0113	1.12	0.00	1.12	2.23
			2016	0	94	0.0000	0.0057	0.0016	0.0055	0.0031	0.0093	1.08	0.00	1.08	2.15
			2017	0	88	0.0000	0.0048	0.0014	0.0047	0.0026	0.0080	0.85	0.00	0.85	1.70
			2018	0	111	0.0000	0.0049	0.0013	0.0047	0.0028	0.0077	1.09	0.00	1.09	2.17
			2019	0	170	0.0000	0.0039	0.0010	0.0038	0.0022	0.0062	1.33	0.00	1.33	2.66

Table 16: Deviance summaries for UAM data on HIV prevalence among current PWID attending services.

Age	Sex	Year	Observed proportion aware			Posterior summaries					Deviance summaries			
			Aware	Positive	Proportion	Mean	SD	Median	95% CrI		$\overline{D}$	$D(\overline{\psi})$	p_D	DIC
15-34	Men	2013	22	22	1.00	0.96	0.02	0.96	0.90	0.99	1.83	0.00	1.83	3.65
		2014	19	19	1.00	0.94	0.03	0.95	0.86	0.99	2.22	0.00	2.22	4.43
		2015	17	17	1.00	0.90	0.05	0.91	0.78	0.97	3.65	0.00	3.65	7.31
		2016	13	13	1.00	0.89	0.06	0.91	0.76	0.97	2.94	0.00	2.94	5.89
		2017	12	12	1.00	0.92	0.05	0.93	0.79	0.98	2.13	0.00	2.13	4.27
		2018	7	7	1.00	0.94	0.05	0.96	0.82	0.99	0.82	0.00	0.82	1.64
		2019	7	7	1.00	0.96	0.04	0.97	0.85	1.00	0.58	0.00	0.58	1.16
	Women	2013	5	7	0.71	0.76	0.11	0.78	0.50	0.93	0.68	0.00	0.68	1.36
		2014	3	4	0.75	0.71	0.13	0.72	0.42	0.91	0.37	0.00	0.37	0.75
		2015	2	3	0.67	0.57	0.15	0.57	0.27	0.84	0.43	0.00	0.43	0.86
		2016	3	5	0.60	0.56	0.14	0.56	0.27	0.82	0.51	0.00	0.51	1.01
		2017	3	5	0.60	0.61	0.15	0.62	0.31	0.86	0.52	0.00	0.52	1.04
		2018	2	4	0.50	0.68	0.17	0.70	0.31	0.93	1.37	0.00	1.37	2.74
		2019	1	2	0.50	0.76	0.17	0.80	0.33	0.98	1.30	0.00	1.30	2.60
35-44	Men	2013	31	31	1.00	0.98	0.01	0.99	0.95	1.00	1.10	0.00	1.10	2.20
		2014	28	29	0.97	0.97	0.02	0.98	0.93	1.00	0.53	0.00	0.53	1.06
		2015	22	25	0.88	0.93	0.04	0.94	0.81	0.98	1.99	0.00	1.99	3.98
		2016	14	17	0.82	0.90	0.07	0.92	0.73	0.98	2.48	0.00	2.48	4.96
		2017	8	10	0.80	0.92	0.08	0.94	0.69	0.99	2.84	0.00	2.84	5.68
		2018	10	10	1.00	0.98	0.02	0.98	0.92	1.00	0.48	0.00	0.48	0.96
		2019	12	12	1.00	0.98	0.02	0.99	0.94	1.00	0.39	0.00	0.39	0.79
	Women	2013	9	9	1.00	0.90	0.06	0.91	0.76	0.98	1.95	0.00	1.95	3.89
		2014	7	7	1.00	0.86	0.07	0.88	0.70	0.97	2.09	0.00	2.09	4.17
		2015	5	6	0.83	0.76	0.11	0.78	0.52	0.93	0.50	0.00	0.50	1.01
		2016	3	4	0.75	0.75	0.12	0.76	0.47	0.93	0.34	0.00	0.34	0.68
		2017	6	7	0.86	0.80	0.10	0.82	0.59	0.95	0.50	0.00	0.50	1.00
		2018	6	6	1.00	0.88	0.08	0.89	0.68	0.98	1.62	0.00	1.62	3.23
		2019	7	7	1.00	0.91	0.07	0.93	0.74	0.99	1.32	0.00	1.32	2.64
45-59	Men	2013	17	19	0.89	0.95	0.04	0.96	0.84	0.99	2.01	0.00	2.01	4.03
		2014	17	19	0.89	0.94	0.04	0.95	0.84	0.99	1.43	0.00	1.43	2.86
		2015	14	15	0.93	0.92	0.04	0.92	0.81	0.98	0.38	0.00	0.38	0.76
		2016	16	17	0.94	0.91	0.04	0.92	0.81	0.98	0.48	0.00	0.48	0.96
		2017	15	16	0.94	0.93	0.04	0.94	0.83	0.98	0.39	0.00	0.39	0.77
		2018	19	20	0.95	0.96	0.03	0.96	0.88	0.99	0.58	0.00	0.58	1.17
		2019	23	24	0.96	0.97	0.03	0.98	0.90	1.00	0.78	0.00	0.78	1.57
	Women	2013	3	4	0.75	0.80	0.11	0.82	0.53	0.95	0.43	0.00	0.43	0.87
		2014	3	5	0.60	0.73	0.13	0.75	0.42	0.92	0.98	0.00	0.98	1.97
		2015	0	2	0.00	0.47	0.24	0.51	0.00	0.83	2.94	0.00	2.94	5.88
		2016	1	2	0.50	0.60	0.15	0.61	0.26	0.85	0.32	0.00	0.32	0.64
		2017	1	1	1.00	0.67	0.14	0.69	0.35	0.90	0.85	0.00	0.85	1.70
		2018	2	2	1.00	0.79	0.13	0.81	0.49	0.97	1.03	0.00	1.03	2.06
		2019	2	2	1.00	0.84	0.12	0.86	0.55	0.98	0.75	0.00	0.75	1.50

Table 17: Deviance summaries for UAM data on proportion aware of their infection status among HIV-positive current PWID attending services.

Age	Sex	Region	Year	Data	Posterior summaries: Mean	SD	Median	95% CrI		Deviance summaries: $\overline{D}$	$D(\overline{\psi})$	p_D	DIC
15-34	Men	London	2013	4,153	4,135	63	4,135	4,014	4,260	1.03	0.08	0.95	1.99
			2014	4,387	4,370	65	4,369	4,244	4,499	1.04	0.07	0.97	2.01
			2015	4,624	4,588	66	4,588	4,459	4,717	1.24	0.29	0.96	2.20
			2016	4,547	4,534	64	4,534	4,410	4,661	0.95	0.04	0.91	1.86
			2017	4,345	4,367	61	4,367	4,249	4,488	0.96	0.11	0.85	1.81
			2018	4,059	4,117	59	4,117	4,005	4,234	1.64	0.82	0.82	2.46
			2019	3,969	3,990	59	3,989	3,877	4,105	0.97	0.11	0.86	1.83
		Outside London	2013	4,663	4,687	67	4,686	4,557	4,820	1.08	0.12	0.96	2.04
			2014	4,859	4,898	68	4,897	4,765	5,034	1.25	0.30	0.95	2.20
			2015	5,126	5,175	70	5,175	5,040	5,315	1.41	0.47	0.94	2.35
			2016	5,213	5,247	71	5,247	5,109	5,388	1.18	0.22	0.96	2.13
			2017	5,166	5,197	72	5,197	5,058	5,339	1.17	0.19	0.98	2.16
			2018	5,034	5,060	71	5,060	4,921	5,199	1.12	0.13	0.98	2.10
			2019	4,967	5,014	70	5,013	4,878	5,152	1.39	0.43	0.96	2.35
	Women	London	2013	1,889	1,871	43	1,871	1,787	1,955	1.16	0.17	0.99	2.14
			2014	1,757	1,736	42	1,736	1,655	1,819	1.27	0.25	1.02	2.29
			2015	1,544	1,523	39	1,522	1,447	1,600	1.32	0.30	1.02	2.33
			2016	1,352	1,330	36	1,329	1,261	1,402	1.38	0.37	1.01	2.38
			2017	1,170	1,159	32	1,159	1,099	1,222	0.97	0.10	0.87	1.84
			2018	1,021	1,035	28	1,034	984	1,091	0.90	0.18	0.72	1.63
			2019	917	918	26	918	869	970	0.72	0.00	0.71	1.43
		Outside London	2013	3,042	3,061	54	3,061	2,956	3,167	1.06	0.12	0.94	2.00
			2014	2,859	2,885	52	2,885	2,786	2,988	1.15	0.24	0.91	2.07
			2015	2,608	2,647	48	2,647	2,554	2,743	1.44	0.58	0.86	2.30
			2016	2,350	2,379	46	2,378	2,293	2,471	1.22	0.36	0.86	2.08
			2017	2,116	2,132	43	2,132	2,049	2,218	0.98	0.12	0.86	1.83
			2018	1,966	1,980	41	1,980	1,899	2,063	0.96	0.10	0.86	1.82
			2019	1,811	1,865	39	1,865	1,791	1,944	2.36	1.58	0.78	3.14
35-44	Men	London	2013	7,189	7,171	85	7,170	7,003	7,338	1.05	0.05	1.01	2.06
			2014	7,136	7,119	83	7,118	6,957	7,284	1.02	0.04	0.98	2.00
			2015	7,095	7,079	83	7,079	6,921	7,247	1.01	0.03	0.98	1.99
			2016	6,835	6,833	82	6,832	6,670	6,992	0.99	0.00	0.99	1.99
			2017	6,630	6,647	79	6,647	6,490	6,802	0.99	0.04	0.94	1.93
			2018	6,311	6,332	78	6,333	6,180	6,483	1.02	0.07	0.95	1.97
			2019	6,416	6,417	79	6,416	6,265	6,574	0.97	0.00	0.97	1.93
		Outside London	2013	7,888	7,900	89	7,900	7,726	8,074	1.01	0.02	0.99	2.01
			2014	7,921	7,930	89	7,929	7,764	8,108	1.01	0.01	1.00	2.00
			2015	8,172	8,178	91	8,177	8,000	8,359	1.01	0.00	1.00	2.01
			2016	8,114	8,112	90	8,111	7,938	8,292	1.00	0.00	1.00	1.99
			2017	8,056	8,048	90	8,047	7,871	8,224	1.01	0.01	1.00	2.02
			2018	7,944	7,932	89	7,931	7,759	8,110	1.03	0.02	1.01	2.04
			2019	7,789	7,785	88	7,784	7,615	7,962	0.99	0.00	0.99	1.97
	Women	London	2013	3,861	3,858	62	3,858	3,738	3,984	1.01	0.00	1.01	2.02
			2014	3,826	3,822	62	3,821	3,704	3,943	1.01	0.00	1.00	2.01
			2015	3,677	3,677	60	3,677	3,559	3,798	0.99	0.00	0.99	1.98
			2016	3,501	3,502	59	3,502	3,387	3,617	0.98	0.00	0.98	1.96
			2017	3,333	3,337	57	3,337	3,225	3,451	0.98	0.01	0.98	1.96
			2018	3,009	3,013	54	3,013	2,907	3,119	0.98	0.00	0.97	1.95
			2019	2,840	2,844	53	2,844	2,741	2,951	1.01	0.01	1.00	2.01
		Outside London	2013	6,034	6,035	78	6,034	5,884	6,186	1.01	0.00	1.01	2.02
			2014	6,125	6,128	78	6,127	5,977	6,281	0.99	0.00	0.98	1.97
			2015	6,122	6,121	78	6,122	5,970	6,277	0.99	0.00	0.99	1.97
			2016	6,170	6,167	78	6,167	6,015	6,322	1.00	0.00	1.00	1.99
			2017	6,087	6,081	78	6,080	5,929	6,233	1.00	0.01	0.99	1.99
			2018	5,956	5,949	78	5,949	5,797	6,101	1.02	0.01	1.01	2.04
			2019	5,766	5,759	75	5,759	5,615	5,906	0.98	0.01	0.97	1.96

Age	Sex	Region	Year	Data	Posterior summaries: Mean	SD	Median	95% CrI		Deviance summaries: $\overline{D}$	$D(\overline{\psi})$	p_D	DIC
45-59	Men	London	2013	9,927	9,916	98	9,915	9,724	10,111	0.99	0.01	0.98	1.97
			2014	10,667	10,654	103	10,655	10,451	10,854	1.01	0.02	1.00	2.01
			2015	11,270	11,257	106	11,256	11,047	11,465	1.02	0.02	1.01	2.03
			2016	11,638	11,627	106	11,626	11,420	11,836	0.98	0.01	0.97	1.95
			2017	12,009	11,997	109	11,997	11,789	12,210	1.01	0.01	0.99	2.00
			2018	11,935	11,920	108	11,920	11,709	12,132	1.01	0.02	0.99	1.99
			2019	12,469	12,457	111	12,456	12,240	12,684	1.01	0.01	1.00	2.00
		Outside London	2013	10,555	10,546	102	10,546	10,348	10,749	1.00	0.01	0.99	1.99
			2014	11,528	11,521	108	11,520	11,306	11,732	1.02	0.00	1.01	2.03
			2015	12,587	12,576	111	12,577	12,359	12,795	1.00	0.01	0.99	1.98
			2016	13,666	13,657	116	13,657	13,432	13,885	0.99	0.01	0.98	1.97
			2017	14,442	14,433	118	14,434	14,200	14,664	0.98	0.01	0.97	1.95
			2018	15,257	15,248	124	15,246	15,005	15,491	1.01	0.01	1.01	2.02
			2019	15,793	15,784	125	15,782	15,540	16,027	1.00	0.01	0.99	1.99
	Women	London	2013	3,579	3,573	60	3,573	3,456	3,693	1.01	0.01	1.00	2.00
			2014	3,956	3,950	63	3,949	3,831	4,075	1.01	0.01	1.00	2.02
			2015	4,335	4,329	66	4,329	4,198	4,457	1.01	0.01	1.00	2.01
			2016	4,573	4,566	67	4,566	4,435	4,699	1.00	0.01	0.99	1.99
			2017	4,893	4,886	69	4,886	4,753	5,022	0.99	0.01	0.98	1.97
			2018	5,083	5,075	72	5,076	4,930	5,213	1.04	0.01	1.02	2.06
			2019	5,456	5,450	73	5,450	5,308	5,594	0.99	0.01	0.98	1.96
		Outside London	2013	3,959	3,954	63	3,953	3,830	4,079	1.01	0.01	1.00	2.02
			2014	4,535	4,529	66	4,529	4,401	4,660	0.99	0.01	0.98	1.96
			2015	5,246	5,242	72	5,243	5,099	5,382	1.00	0.00	1.00	2.00
			2016	5,863	5,857	76	5,857	5,713	6,008	1.00	0.01	0.99	1.99
			2017	6,457	6,452	81	6,451	6,293	6,609	1.01	0.00	1.01	2.02
			2018	7,057	7,050	84	7,050	6,886	7,213	1.01	0.01	1.00	2.01
			2019	7,683	7,678	87	7,677	7,509	7,852	0.99	0.00	0.99	1.99
60-74	Men	London	2013	1,623	1,626	40	1,625	1,550	1,706	0.98	0.00	0.98	1.96
			2014	1,826	1,829	43	1,828	1,745	1,914	1.01	0.00	1.01	2.02
			2015	2,037	2,040	45	2,040	1,952	2,129	1.01	0.00	1.00	2.01
			2016	2,292	2,295	48	2,294	2,202	2,390	1.00	0.00	1.00	2.00
			2017	2,576	2,578	51	2,578	2,478	2,679	1.02	0.00	1.02	2.03
			2018	2,779	2,779	53	2,779	2,678	2,885	1.00	0.00	1.00	2.01
			2019	3,244	3,246	57	3,246	3,134	3,358	1.00	0.00	1.00	2.01
		Outside London	2013	2,400	2,401	49	2,400	2,305	2,500	1.00	0.00	1.00	2.00
			2014	2,779	2,781	52	2,781	2,679	2,885	0.99	0.00	0.99	1.98
			2015	3,128	3,130	55	3,130	3,022	3,238	0.97	0.00	0.97	1.95
			2016	3,529	3,531	59	3,531	3,417	3,648	0.99	0.00	0.99	1.99
			2017	3,966	3,968	64	3,969	3,844	4,095	1.03	0.00	1.03	2.06
			2018	4,460	4,463	68	4,462	4,329	4,596	1.03	0.00	1.03	2.06
			2019	4,949	4,950	70	4,951	4,813	5,085	0.99	0.00	0.99	1.98
	Women	London	2013	429	434	21	433	394	475	1.03	0.05	0.98	2.01
			2014	498	501	22	501	460	545	0.99	0.02	0.97	1.96
			2015	566	569	24	569	523	617	1.01	0.02	0.99	2.00
			2016	658	661	25	661	612	711	0.99	0.02	0.97	1.96
			2017	759	762	27	762	710	818	1.00	0.01	0.98	1.98
			2018	874	878	30	878	820	938	1.05	0.02	1.03	2.08
			2019	1,033	1,036	32	1,036	975	1,099	0.98	0.01	0.97	1.95
		Outside London	2013	541	545	23	545	500	592	1.01	0.03	0.98	1.99
			2014	646	649	25	649	600	700	0.99	0.01	0.98	1.97
			2015	753	755	28	755	702	810	1.02	0.01	1.01	2.04
			2016	887	890	29	890	833	949	0.98	0.01	0.97	1.96
			2017	1,014	1,017	32	1,017	956	1,080	1.00	0.01	0.99	1.99
			2018	1,198	1,201	35	1,201	1,134	1,269	1.01	0.01	1.00	2.00
			2019	1,379	1,383	37	1,382	1,311	1,456	1.00	0.01	0.99	1.99

Table 18: Deviance summaries for HARS data on total number of individuals living with diagnosed HIV.

			Observed number diagnosed				Posterior mean number diagnosed							
Age	Region	Year	GBM	PWID	Heterosexuals (Black African)	Heterosexuals (other ethnicities)	GBM	PWID	Heterosexuals (Black African)	Heterosexuals (other ethnicities)	$\overline{D}$	$D(\overline{\psi})$	p_D	DIC
15-34	London	2013	3,489	49	235	297	3,532	65	236	298	6.65	3.76	2.89	9.54
		2014	3,761	48	220	281	3,812	61	226	272	6.26	3.32	2.94	9.20
		2015	3,975	47	210	273	4,043	60	204	269	6.16	3.24	2.92	9.09
		2016	3,928	35	183	275	4,031	46	183	276	5.28	2.67	2.60	7.88
		2017	3,762	26	175	239	3,939	37	182	242	5.18	2.92	2.26	7.44
		2018	3,511	20	147	224	3,722	34	173	231	9.04	7.21	1.83	10.87
		2019	3,397	17	139	206	3,605	29	158	214	7.08	5.11	1.96	9.04
	Outside London	2013	3,672	104	368	485	3,702	127	376	506	7.59	4.17	3.41	11.00
		2014	3,937	95	298	497	3,959	115	316	528	8.33	5.10	3.22	11.55
		2015	4,230	74	251	516	4,274	93	285	543	10.05	7.16	2.90	12.95
		2016	4,342	64	216	521	4,394	80	243	543	8.20	5.33	2.88	11.08
		2017	4,330	55	191	496	4,389	69	233	510	11.82	9.08	2.74	14.57
		2018	4,211	49	165	478	4,296	64	207	491	12.93	10.30	2.64	15.57
		2019	4,160	43	145	481	4,260	56	205	496	20.89	18.34	2.55	23.44
35-44	London	2013	5,284	149	941	683	5,358	180	958	689	9.09	4.91	4.17	13.26
		2014	5,306	146	854	689	5,388	177	870	697	8.97	4.86	4.11	13.07
		2015	5,372	149	740	684	5,465	181	756	692	9.09	4.94	4.16	13.25
		2016	5,246	131	660	642	5,368	160	676	652	8.22	4.25	3.97	12.19
		2017	5,132	130	588	595	5,323	159	606	608	7.83	3.92	3.91	11.74
		2018	4,918	108	543	531	5,126	133	568	547	6.95	3.21	3.73	10.68
		2019	5,000	96	501	544	5,220	120	529	565	7.05	3.32	3.73	10.78
	Outside London	2013	4,742	244	1,770	1,069	4,793	292	1,780	1,087	12.01	7.30	4.71	16.72
		2014	4,818	254	1,692	1,085	4,872	305	1,703	1,102	12.41	7.61	4.79	17.20
		2015	5,083	269	1,657	1,089	5,135	323	1,668	1,105	13.20	8.23	4.97	18.17
		2016	5,116	258	1,521	1,117	5,174	311	1,538	1,135	12.82	8.01	4.81	17.64
		2017	5,197	242	1,382	1,106	5,264	292	1,403	1,126	12.38	7.59	4.79	17.17
		2018	5,238	231	1,226	1,098	5,319	281	1,245	1,121	12.20	7.56	4.64	16.84
		2019	5,221	225	1,080	1,090	5,329	274	1,103	1,116	11.64	7.02	4.62	16.26
45-59	London	2013	6,661	221	1,805	1,123	6,733	260	1,822	1,134	10.04	5.38	4.65	14.69
		2014	7,185	233	1,939	1,172	7,267	275	1,961	1,184	10.34	5.60	4.74	15.08
		2015	7,554	234	2,054	1,241	7,670	276	2,085	1,260	10.25	5.48	4.78	15.03
		2016	7,878	228	2,086	1,251	8,000	270	2,118	1,270	10.31	5.50	4.81	15.11
		2017	8,140	239	2,110	1,291	8,282	283	2,148	1,313	10.63	5.75	4.88	15.51
		2018	8,083	225	2,078	1,289	8,248	267	2,121	1,314	10.16	5.40	4.76	14.91
		2019	8,451	237	2,114	1,332	8,671	283	2,168	1,366	10.53	5.67	4.85	15.38
	Outside London	2013	6,628	231	1,782	1,828	6,674	275	1,795	1,839	11.48	6.59	4.89	16.37
		2014	7,256	233	1,970	1,963	7,315	277	1,986	1,978	11.65	6.66	4.99	16.65
		2015	7,875	250	2,205	2,116	7,953	298	2,228	2,136	12.25	7.03	5.21	17.46
		2016	8,555	282	2,414	2,242	8,656	336	2,442	2,268	13.28	7.88	5.40	18.67
		2017	8,972	297	2,597	2,355	9,104	355	2,633	2,388	13.80	8.25	5.55	19.35
		2018	9,339	313	2,867	2,477	9,493	374	2,913	2,517	14.28	8.53	5.74	20.02
		2019	9,586	323	3,033	2,538	9,771	388	3,090	2,586	14.68	8.92	5.77	20.45
60-74	London	2013	1,118	18	155	312	1,134	22	157	316	3.95	0.82	3.13	7.08
		2014	1,231	23	194	353	1,250	29	197	358	4.30	1.01	3.29	7.59
		2015	1,353	28	243	379	1,377	35	248	385	4.54	1.23	3.31	7.84
		2016	1,500	33	288	426	1,532	41	295	434	4.81	1.48	3.33	8.14
		2017	1,670	39	349	469	1,704	49	356	478	5.10	1.69	3.41	8.52
		2018	1,798	43	396	491	1,833	54	402	500	5.28	1.85	3.43	8.71
		2019	2,071	49	487	569	2,118	61	497	581	5.51	2.00	3.51	9.02
	Outside London	2013	1,479	19	138	738	1,497	24	140	744	4.05	0.83	3.22	7.27
		2014	1,718	26	172	822	1,746	32	176	833	4.35	1.04	3.31	7.65
		2015	1,928	34	209	905	1,963	42	214	919	4.77	1.36	3.41	8.18
		2016	2,186	35	249	994	2,229	43	254	1,012	4.74	1.30	3.44	8.18
		2017	2,478	39	296	1,078	2,528	48	303	1,098	5.05	1.53	3.52	8.57
		2018	2,773	45	358	1,192	2,833	56	366	1,216	5.33	1.76	3.57	8.90
		2019	3,082	54	427	1,272	3,158	67	437	1,301	5.68	2.01	3.67	9.36

Table 19: Deviance summaries for HARS data on proportions of men living with diagnosed HIV who are in each exposure group.

Age	Region	Year	Observed number diagnosed: PWID	Observed number diagnosed: Heterosexuals (Black African)	Observed number diagnosed: Heterosexuals (other ethnicities)	Posterior mean number diagnosed: PWID	Posterior mean number diagnosed: Heterosexuals (Black African)	Posterior mean number diagnosed: Heterosexuals (other ethnicities)	$\overline{D}$	$D(\overline{\psi})$	p_D	DIC
15-34		2013	25	1,152	675	32	1,163	678	3.61	1.36	2.25	5.86
		2014	27	1,024	650	34	1,042	661	3.65	1.41	2.24	5.89
		2015	20	882	578	26	900	597	3.47	1.25	2.21	5.68
	London	2016	17	742	530	22	764	543	3.21	1.10	2.11	5.32
		2017	11	609	478	15	639	505	2.75	0.89	1.86	4.61
		2018	5	526	402	9	577	451	3.08	1.85	1.23	4.31
		2019	4	427	376	8	488	423	3.05	1.90	1.16	4.21
		2013	59	1,708	1,222	73	1,757	1,250	4.52	2.03	2.49	7.00
		2014	64	1,526	1,208	79	1,582	1,245	4.53	2.09	2.44	6.97
		2015	59	1,302	1,188	73	1,375	1,224	4.37	2.03	2.33	6.70
	Outside London	2016	49	1,109	1,128	61	1,167	1,169	4.00	1.72	2.28	6.27
		2017	36	951	1,060	45	999	1,099	3.56	1.44	2.12	5.69
		2018	35	815	1,033	45	879	1,068	4.23	2.15	2.08	6.31
		2019	31	710	972	40	819	1,025	6.08	4.15	1.93	8.00
35-44		2013	50	2,770	997	60	2,801	1,006	4.07	1.62	2.44	6.51
		2014	45	2,720	1,006	55	2,757	1,017	3.93	1.53	2.40	6.33
		2015	48	2,577	984	58	2,626	1,002	3.92	1.48	2.44	6.36
	London	2016	39	2,432	956	48	2,487	976	3.65	1.29	2.36	6.00
		2017	39	2,287	913	48	2,359	939	3.64	1.29	2.34	5.98
		2018	40	2,008	852	50	2,087	884	3.71	1.35	2.35	6.06
		2019	38	1,842	847	48	1,923	882	3.61	1.31	2.30	5.91
		2013	71	4,315	1,627	85	4,327	1,635	5.18	2.47	2.70	7.88
		2014	72	4,279	1,734	87	4,309	1,747	5.19	2.46	2.73	7.92
		2015	77	4,224	1,774	93	4,255	1,787	5.36	2.58	2.78	8.14
	Outside London	2016	77	4,190	1,839	93	4,227	1,858	5.39	2.62	2.77	8.16
		2017	84	4,059	1,867	102	4,101	1,890	5.63	2.82	2.81	8.44
		2018	90	3,868	1,911	109	3,913	1,938	5.96	3.09	2.87	8.83
		2019	82	3,676	1,886	100	3,743	1,927	5.60	2.82	2.78	8.38
45-59		2013	69	2,481	986	81	2,508	996	4.13	1.58	2.55	6.68
		2014	76	2,744	1,083	89	2,778	1,095	4.27	1.68	2.58	6.85
		2015	77	3,030	1,165	90	3,071	1,180	4.08	1.49	2.59	6.67
	London	2016	78	3,179	1,229	91	3,237	1,250	4.12	1.49	2.64	6.76
		2017	78	3,383	1,320	92	3,457	1,348	4.19	1.57	2.62	6.80
		2018	75	3,542	1,333	89	3,631	1,366	4.23	1.60	2.63	6.86
		2019	71	3,808	1,408	85	3,925	1,450	4.30	1.63	2.67	6.97
		2013	66	2,542	1,312	79	2,562	1,323	4.48	1.92	2.56	7.04
		2014	81	2,916	1,483	97	2,947	1,498	4.99	2.29	2.70	7.69
		2015	88	3,391	1,707	104	3,427	1,725	5.03	2.31	2.72	7.76
	Outside London	2016	92	3,799	1,898	109	3,843	1,921	5.06	2.25	2.81	7.86
		2017	89	4,231	2,046	106	4,287	2,073	5.24	2.32	2.91	8.15
		2018	93	4,691	2,179	110	4,748	2,206	5.40	2.44	2.97	8.37
		2019	98	5,091	2,371	116	5,166	2,409	5.59	2.51	3.08	8.67
60-74		2013	3	252	167	4	258	172	2.22	0.41	1.81	4.03
		2014	4	296	192	5	301	196	2.33	0.38	1.95	4.28
		2015	5	345	212	7	348	215	2.50	0.45	2.05	4.54
	London	2016	7	397	243	9	405	249	2.60	0.59	2.02	4.62
		2017	8	448	284	11	460	293	2.73	0.66	2.07	4.80
		2018	10	517	317	13	537	330	2.83	0.72	2.11	4.94
		2019	17	621	360	22	644	374	3.16	1.01	2.15	5.31
		2013	2	224	304	4	229	313	2.33	0.69	1.64	3.97
		2014	3	276	349	4	284	362	2.20	0.41	1.80	4.00
		2015	3	340	390	4	349	403	2.20	0.36	1.84	4.04
	Outside London	2016	7	408	447	9	420	463	2.59	0.48	2.10	4.69
		2017	10	458	517	13	472	534	2.75	0.67	2.08	4.84
		2018	12	548	606	16	564	625	2.87	0.72	2.15	5.03
		2019	12	638	686	16	660	710	2.90	0.71	2.20	5.10

Table 20: Deviance summaries for HARS data on proportions of women living with diagnosed HIV who are in each exposure group.

				Observed proportion eligible			Posterior summaries					Deviance summaries			
Group	Gender	Region	Year	Eligible	Attending	Proportion	Mean	SD	Median	95% CrI		$\overline{D}$	$D(\overline{\psi})$	p_D	DIC
GBM	Men	London	2013	26,990	29,707	0.91	0.91	0.00	0.91	0.90	0.91	1.02	0.00	1.02	2.03
			2014	32,940	36,054	0.91	0.91	0.00	0.91	0.91	0.92	0.98	0.00	0.98	1.95
			2015	34,940	38,156	0.92	0.92	0.00	0.92	0.91	0.92	1.02	0.00	1.02	2.04
			2016	34,853	38,410	0.91	0.91	0.00	0.91	0.90	0.91	0.94	0.00	0.94	1.89
			2017	35,259	38,937	0.91	0.91	0.00	0.91	0.90	0.91	0.99	0.00	0.99	1.98
			2018	34,363	37,872	0.91	0.91	0.00	0.91	0.91	0.91	1.12	0.00	1.12	2.25
			2019	35,173	38,504	0.91	0.91	0.00	0.91	0.91	0.92	0.89	0.00	0.89	1.77
		Outside London	2013	30,056	32,791	0.92	0.92	0.00	0.92	0.91	0.92	0.99	0.00	0.99	1.98
			2014	34,625	37,617	0.92	0.92	0.00	0.92	0.92	0.92	0.99	0.00	0.99	1.98
			2015	38,018	41,220	0.92	0.92	0.00	0.92	0.92	0.92	1.00	0.00	1.00	1.99
			2016	38,774	42,031	0.92	0.92	0.00	0.92	0.92	0.92	0.97	0.00	0.97	1.94
			2017	43,033	46,385	0.93	0.93	0.00	0.93	0.93	0.93	1.00	0.00	1.00	2.00
			2018	45,872	49,116	0.93	0.93	0.00	0.93	0.93	0.94	1.05	0.00	1.05	2.10
			2019	49,534	53,009	0.93	0.93	0.00	0.93	0.93	0.94	1.00	0.00	1.00	1.99
Heterosexuals (Black African)	Men	London	2013	11,580	11,759	0.98	0.98	0.00	0.98	0.98	0.99	0.96	0.00	0.96	1.92
			2014	11,594	11,799	0.98	0.98	0.00	0.98	0.98	0.98	0.72	0.00	0.72	1.44
			2015	11,656	11,824	0.99	0.98	0.00	0.98	0.98	0.99	1.45	0.00	1.45	2.89
			2016	11,669	11,830	0.99	0.99	0.00	0.99	0.98	0.99	0.93	0.00	0.93	1.86
			2017	11,371	11,547	0.98	0.98	0.00	0.98	0.98	0.99	0.61	0.00	0.61	1.23
			2018	11,186	11,375	0.98	0.98	0.00	0.98	0.98	0.99	2.15	0.00	2.15	4.29
			2019	10,997	11,163	0.99	0.99	0.00	0.99	0.98	0.99	1.09	0.00	1.09	2.18
		Outside London	2013	8,331	8,651	0.96	0.96	0.00	0.96	0.96	0.97	0.94	0.00	0.94	1.87
			2014	8,408	8,718	0.96	0.97	0.00	0.97	0.96	0.97	1.33	0.00	1.33	2.66
			2015	8,529	8,832	0.97	0.97	0.00	0.97	0.97	0.97	3.27	0.00	3.27	6.54
			2016	9,081	9,332	0.97	0.98	0.00	0.98	0.97	0.98	2.41	0.00	2.41	4.83
			2017	9,606	9,870	0.97	0.98	0.00	0.98	0.97	0.98	5.25	0.00	5.25	10.51
			2018	10,363	10,602	0.98	0.98	0.00	0.98	0.98	0.98	5.64	0.00	5.64	11.28
			2019	11,401	11,658	0.98	0.98	0.00	0.98	0.98	0.98	11.77	0.00	11.77	23.54
	Women	London	2013	18,246	19,035	0.96	0.96	0.00	0.96	0.96	0.96	1.08	0.00	1.08	2.16
			2014	18,818	19,536	0.96	0.96	0.00	0.96	0.96	0.97	1.20	0.00	1.20	2.39
			2015	18,937	19,577	0.97	0.97	0.00	0.97	0.96	0.97	1.36	0.00	1.36	2.73
			2016	18,274	18,880	0.97	0.97	0.00	0.97	0.96	0.97	1.20	0.00	1.20	2.40
			2017	17,861	18,431	0.97	0.97	0.00	0.97	0.97	0.97	0.88	0.00	0.88	1.75
			2018	17,685	18,231	0.97	0.97	0.00	0.97	0.97	0.97	0.68	0.00	0.68	1.37
			2019	17,607	18,075	0.97	0.97	0.00	0.97	0.97	0.98	0.65	0.00	0.65	1.30
		Outside London	2013	9,836	11,308	0.87	0.87	0.00	0.87	0.86	0.88	1.01	0.00	1.01	2.01
			2014	11,056	12,398	0.89	0.89	0.00	0.89	0.89	0.90	1.12	0.00	1.12	2.25
			2015	12,048	13,305	0.91	0.91	0.00	0.91	0.90	0.91	1.57	0.00	1.57	3.14
			2016	13,000	14,069	0.92	0.93	0.00	0.93	0.92	0.93	1.23	0.00	1.23	2.45
			2017	14,725	15,667	0.94	0.94	0.00	0.94	0.94	0.94	0.90	0.00	0.90	1.80
			2018	15,753	16,609	0.95	0.95	0.00	0.95	0.95	0.95	1.17	0.00	1.17	2.34
			2019	17,167	18,015	0.95	0.96	0.00	0.96	0.95	0.96	3.80	0.00	3.80	7.60
Heterosexuals (other ethnicities)	Men	London	2013	85,821	86,057	1.00	1.00	0.00	1.00	1.00	1.00	0.96	0.00	0.96	1.92
			2014	85,385	85,556	1.00	1.00	0.00	1.00	1.00	1.00	1.61	0.00	1.61	3.21
			2015	88,370	88,561	1.00	1.00	0.00	1.00	1.00	1.00	1.31	0.00	1.31	2.61
			2016	87,761	87,990	1.00	1.00	0.00	1.00	1.00	1.00	0.96	0.00	0.96	1.92
			2017	77,617	77,818	1.00	1.00	0.00	1.00	1.00	1.00	0.91	0.00	0.91	1.83
			2018	66,660	66,857	1.00	1.00	0.00	1.00	1.00	1.00	0.80	0.00	0.80	1.60
			2019	57,547	57,717	1.00	1.00	0.00	1.00	1.00	1.00	0.90	0.00	0.90	1.80
		Outside London	2013	256,107	256,591	1.00	1.00	0.00	1.00	1.00	1.00	1.25	0.00	1.25	2.51
			2014	261,720	262,252	1.00	1.00	0.00	1.00	1.00	1.00	1.85	0.00	1.85	3.70
			2015	254,079	254,609	1.00	1.00	0.00	1.00	1.00	1.00	1.47	0.00	1.47	2.94
			2016	248,587	249,096	1.00	1.00	0.00	1.00	1.00	1.00	1.11	0.00	1.11	2.22
			2017	237,498	237,937	1.00	1.00	0.00	1.00	1.00	1.00	0.91	0.00	0.91	1.82
			2018	231,612	232,035	1.00	1.00	0.00	1.00	1.00	1.00	0.92	0.00	0.92	1.83
			2019	224,956	225,368	1.00	1.00	0.00	1.00	1.00	1.00	0.88	0.00	0.88	1.76
	Women	London	2013	160,862	161,321	1.00	1.00	0.00	1.00	1.00	1.00	1.11	0.00	1.11	2.23
			2014	165,778	166,222	1.00	1.00	0.00	1.00	1.00	1.00	1.15	0.00	1.15	2.30
			2015	176,161	176,565	1.00	1.00	0.00	1.00	1.00	1.00	1.05	0.00	1.05	2.11
			2016	178,830	179,226	1.00	1.00	0.00	1.00	1.00	1.00	1.23	0.00	1.23	2.46
			2017	165,322	165,705	1.00	1.00	0.00	1.00	1.00	1.00	0.93	0.00	0.93	1.86
			2018	147,789	148,174	1.00	1.00	0.00	1.00	1.00	1.00	0.96	0.00	0.96	1.92
			2019	140,222	140,562	1.00	1.00	0.00	1.00	1.00	1.00	0.85	0.00	0.85	1.69
		Outside London	2013	397,360	398,280	1.00	1.00	0.00	1.00	1.00	1.00	0.94	0.00	0.94	1.88
			2014	443,898	444,869	1.00	1.00	0.00	1.00	1.00	1.00	0.99	0.00	0.99	1.97
			2015	463,121	464,047	1.00	1.00	0.00	1.00	1.00	1.00	0.98	0.00	0.98	1.96
			2016	480,840	481,744	1.00	1.00	0.00	1.00	1.00	1.00	0.96	0.00	0.96	1.92
			2017	507,052	507,889	1.00	1.00	0.00	1.00	1.00	1.00	0.94	0.00	0.94	1.89
			2018	516,027	516,842	1.00	1.00	0.00	1.00	1.00	1.00	1.08	0.00	1.08	2.17
			2019	512,679	513,502	1.00	1.00	0.00	1.00	1.00	1.00	0.90	0.00	0.90	1.81

Table 21: Deviance summaries for GUMCAD data on proportion of attendees aged 15-34 who are eligible for HIV testing.

Age	Ethnicity	Region	Year	Observed undiagnosed prevalence: New diagnoses	Live births	Proportion	Posterior summaries: Mean	SD	Median	95% CrI		Deviance summaries: $\overline{D}$	$D(\overline{\psi})$	p_D	DIC
15-34	Black African	London	2013	44	11,581	0.00380	0.00376	0.00054	0.00374	0.00276	0.00490	0.92	0.00	0.92	1.85
			2014	27	10,779	0.00250	0.00274	0.00047	0.00270	0.00189	0.00374	1.03	0.00	1.03	2.07
			2015	30	10,040	0.00299	0.00319	0.00052	0.00316	0.00226	0.00430	0.94	0.00	0.94	1.88
			2016	15	9,493	0.00158	0.00171	0.00038	0.00168	0.00105	0.00254	0.84	0.00	0.84	1.69
			2017	15	8,625	0.00174	0.00188	0.00040	0.00186	0.00119	0.00274	0.78	0.00	0.78	1.57
			2018	7	7,677	0.00091	0.00121	0.00031	0.00117	0.00068	0.00191	1.07	0.00	1.07	2.14
			2019	8	7,069	0.00113	0.00141	0.00034	0.00137	0.00085	0.00218	0.88	0.00	0.88	1.75
		Outside London	2013	32	8,915	0.00359	0.00360	0.00059	0.00356	0.00255	0.00486	0.85	0.00	0.85	1.69
			2014	35	8,566	0.00409	0.00395	0.00064	0.00392	0.00280	0.00530	0.95	0.00	0.95	1.90
			2015	41	8,687	0.00472	0.00460	0.00068	0.00456	0.00337	0.00605	0.94	0.00	0.94	1.87
			2016	20	8,681	0.00230	0.00245	0.00048	0.00242	0.00162	0.00348	0.83	0.00	0.83	1.66
			2017	24	8,096	0.00296	0.00307	0.00055	0.00303	0.00208	0.00422	0.79	0.00	0.79	1.59
			2018	21	7,828	0.00268	0.00274	0.00051	0.00270	0.00185	0.00383	0.73	0.00	0.73	1.47
			2019	19	7,891	0.00241	0.00240	0.00047	0.00236	0.00159	0.00342	0.74	0.00	0.74	1.48
	Other ethnicities	London	2013	11	82,566	0.00013	0.00014	0.00003	0.00014	0.00008	0.00022	0.71	0.00	0.71	1.41
			2014	10	81,491	0.00012	0.00013	0.00003	0.00013	0.00007	0.00021	0.70	0.00	0.70	1.41
			2015	7	82,334	0.00009	0.00008	0.00002	0.00008	0.00005	0.00014	0.55	0.00	0.55	1.11
			2016	6	80,962	0.00007	0.00010	0.00003	0.00010	0.00006	0.00017	1.23	0.00	1.23	2.45
			2017	6	78,971	0.00008	0.00009	0.00003	0.00009	0.00005	0.00015	0.66	0.00	0.66	1.33
			2018	9	74,631	0.00012	0.00010	0.00003	0.00010	0.00006	0.00016	0.96	0.00	0.96	1.92
			2019	8	72,437	0.00011	0.00012	0.00003	0.00012	0.00007	0.00019	0.61	0.00	0.61	1.21
		Outside London	2013	30	454,640	0.00007	0.00006	0.00001	0.00006	0.00004	0.00009	1.04	0.00	1.04	2.09
			2014	33	450,084	0.00007	0.00007	0.00001	0.00007	0.00005	0.00010	0.97	0.00	0.97	1.94
			2015	19	446,787	0.00004	0.00004	0.00001	0.00004	0.00003	0.00006	0.76	0.00	0.76	1.53
			2016	38	442,076	0.00009	0.00008	0.00001	0.00008	0.00006	0.00011	1.15	0.00	1.15	2.30
			2017	22	402,420	0.00005	0.00005	0.00001	0.00005	0.00003	0.00008	0.92	0.00	0.92	1.84
			2018	19	387,791	0.00005	0.00005	0.00001	0.00005	0.00003	0.00008	0.96	0.00	0.96	1.91
			2019	21	376,583	0.00006	0.00006	0.00001	0.00005	0.00004	0.00008	0.88	0.00	0.88	1.77
35-44	Black African	London	2013	20	3,816	0.00524	0.00444	0.00090	0.00437	0.00289	0.00640	1.33	0.00	1.33	2.67
			2014	9	3,709	0.00243	0.00304	0.00071	0.00296	0.00187	0.00461	0.97	0.00	0.97	1.94
			2015	12	3,782	0.00317	0.00282	0.00070	0.00274	0.00165	0.00436	0.90	0.00	0.90	1.81
			2016	9	3,618	0.00249	0.00206	0.00054	0.00198	0.00120	0.00333	0.91	0.00	0.91	1.83
			2017	11	3,534	0.00311	0.00189	0.00052	0.00182	0.00109	0.00312	3.12	0.00	3.12	6.24
			2018	3	3,275	0.00092	0.00128	0.00034	0.00124	0.00074	0.00204	0.58	0.00	0.58	1.16
			2019	6	3,219	0.00186	0.00129	0.00036	0.00124	0.00072	0.00214	1.19	0.00	1.19	2.38
		Outside London	2013	9	2,528	0.00356	0.00452	0.00108	0.00440	0.00273	0.00696	1.07	0.00	1.07	2.15
			2014	21	2,623	0.00801	0.00698	0.00134	0.00686	0.00471	0.00989	1.15	0.00	1.15	2.30
			2015	19	2,781	0.00683	0.00688	0.00140	0.00678	0.00442	0.00992	0.79	0.00	0.79	1.59
			2016	10	2,967	0.00337	0.00400	0.00091	0.00391	0.00247	0.00604	0.83	0.00	0.83	1.65
			2017	9	2,852	0.00316	0.00468	0.00106	0.00458	0.00291	0.00705	2.07	0.00	2.07	4.14
			2018	8	2,850	0.00281	0.00400	0.00085	0.00394	0.00256	0.00586	1.50	0.00	1.50	3.01
			2019	9	2,916	0.00309	0.00399	0.00091	0.00390	0.00248	0.00599	1.10	0.00	1.10	2.20
	Other ethnicities	London	2013	3	29,678	0.00010	0.00012	0.00004	0.00011	0.00006	0.00022	0.41	0.00	0.41	0.82
			2014	5	30,721	0.00016	0.00017	0.00006	0.00016	0.00009	0.00031	0.54	0.00	0.54	1.08
			2015	8	32,613	0.00025	0.00018	0.00006	0.00018	0.00009	0.00033	1.55	0.00	1.55	3.09
			2016	2	33,920	0.00006	0.00008	0.00003	0.00008	0.00004	0.00014	0.42	0.00	0.42	0.84
			2017	2	34,309	0.00006	0.00007	0.00003	0.00007	0.00003	0.00015	0.43	0.00	0.43	0.85
			2018	3	34,223	0.00009	0.00008	0.00003	0.00007	0.00004	0.00015	0.44	0.00	0.44	0.88
			2019	0	34,251	0.00000	0.00008	0.00003	0.00007	0.00004	0.00014	5.27	0.00	5.27	10.55
		Outside London	2013	3	102,620	0.00003	0.00004	0.00001	0.00004	0.00002	0.00008	0.93	0.00	0.93	1.86
			2014	5	105,131	0.00005	0.00005	0.00002	0.00005	0.00003	0.00009	0.51	0.00	0.51	1.02
			2015	6	108,491	0.00006	0.00008	0.00002	0.00007	0.00004	0.00013	1.26	0.00	1.26	2.52
			2016	4	112,133	0.00004	0.00005	0.00001	0.00005	0.00003	0.00008	0.83	0.00	0.83	1.67
			2017	7	105,590	0.00007	0.00007	0.00002	0.00006	0.00003	0.00012	0.69	0.00	0.69	1.38
			2018	4	104,961	0.00004	0.00005	0.00002	0.00005	0.00003	0.00009	0.73	0.00	0.73	1.47
			2019	8	103,690	0.00008	0.00006	0.00002	0.00006	0.00003	0.00011	1.07	0.00	1.07	2.15

Table 22: Deviance summaries for NSHPC data on previously undiagnosed (newly diagnosed during current pregnancy) HIV prevalence among pregnant women. Note that the denominator for the observed number of new diagnoses, the number of live births to women by ethnicity, is not directly observed, but is estimated in the model. The columns 'Live births' and 'Proportion' are therefore posterior means.

5.2 Selected posterior predictive checks

Posterior predictive checks compare the observed data to the posterior predictive distribution, the distribution of possible new observations generated by the same process as the one that generated the original data, conditional on the original data. Formally, if the data are $\boldsymbol{y}$, the parameters are $\boldsymbol{\psi}$ with prior distribution $p(\boldsymbol{\psi})$ and therefore the posterior distribution $p(\boldsymbol{\psi} \mid \boldsymbol{y}) \propto p(\boldsymbol{\psi})L(\boldsymbol{y} \mid \boldsymbol{\psi})$, then the posterior predictive distribution for new observations $\boldsymbol{y^{new}} \sim p(\boldsymbol{Y^{new}} \mid \boldsymbol{y})$ is

$$p(\boldsymbol{Y^{new}} \mid \boldsymbol{y}) = \int_{\Psi} p(\boldsymbol{Y^{new}} \mid \boldsymbol{\psi})p(\boldsymbol{\psi} \mid \boldsymbol{y})d\boldsymbol{\psi}$$

where $\boldsymbol{\Psi}$ is the space of possible values of the parameters $\boldsymbol{\psi} \in \boldsymbol{\Psi}$.

Ideally, to check a model, $\boldsymbol{Y^{new}} = \boldsymbol{y^{new}}$ are data that have been left out of the original inference, that are then predicted and the predictive distribution compared to the data used to fit the model $\boldsymbol{y}$, using leave-one-out or leave-k-out cross-validation. If the data $\boldsymbol{y}$ do not lie in the tails of the predictive distribution $p(\boldsymbol{Y^{new}} \mid \boldsymbol{y})$, i.e. if the posterior predictive p-value $p_{\text{pp}} = Pr(\boldsymbol{Y^{new}} \leq \boldsymbol{y} \mid \boldsymbol{y})$ is neither very small nor very large, then we can conclude that the model predicts well the data that have been left out. Very small or large values of p_{pp} indicate a lack of fit. The posterior predictive p-values are derived as the proportion of posterior predictive samples that are smaller than the observation.

However, here the model is just identifiable, so leaving out any data points to be predicted would result in an unidentified model and possible consequent lack of convergence. We therefore carry out posterior predictive checks without cross-validation, using the data twice, both to derive the posterior distribution and to compare to the posterior predictive distribution. Such checks are conservative, i.e. the data may not lie quite as far into the tails of the posterior predictive distribution than would be the case with a cross-validatory posterior predictive check. Conservative checks might miss milder cases of lack of fit, but would still pick up more substantial lack of fit.

Here, the vast majority of data points have been predicted well, with posterior predictive p-values in the range 0.1 to 0.9. We have detected some lack of fit to the prevalence (Figure 15) and group size (Figures 12 to 14) data for PWID. However, given the very small numbers of PWID living with HIV and datedness of the group size information, this lack of fit is not surprising. Work is ongoing to update the estimates of PWID group sizes.

There is also some slight lack of fit to the partitioning of HARS diagnosed cases in men aged 15-34 to the GBM, PWID and heterosexual groups in the most recent years (Figures 18 to 22). However, given the relatively large numbers of diagnosed individuals involved, the differences between observations and predictions do not have a substantial public health interpretation.

Selected posterior predictive checks for the key data sources (PWID group size, prevalence and proportion diagnosed; HARS numbers diagnosed; GUMCAD numbers attending, eligible, offered and accepting HIV tests and newly diagnosed; NSHPC previously undiagnosed infection in pregnant women) are shown in Figures 12 to 30 below.

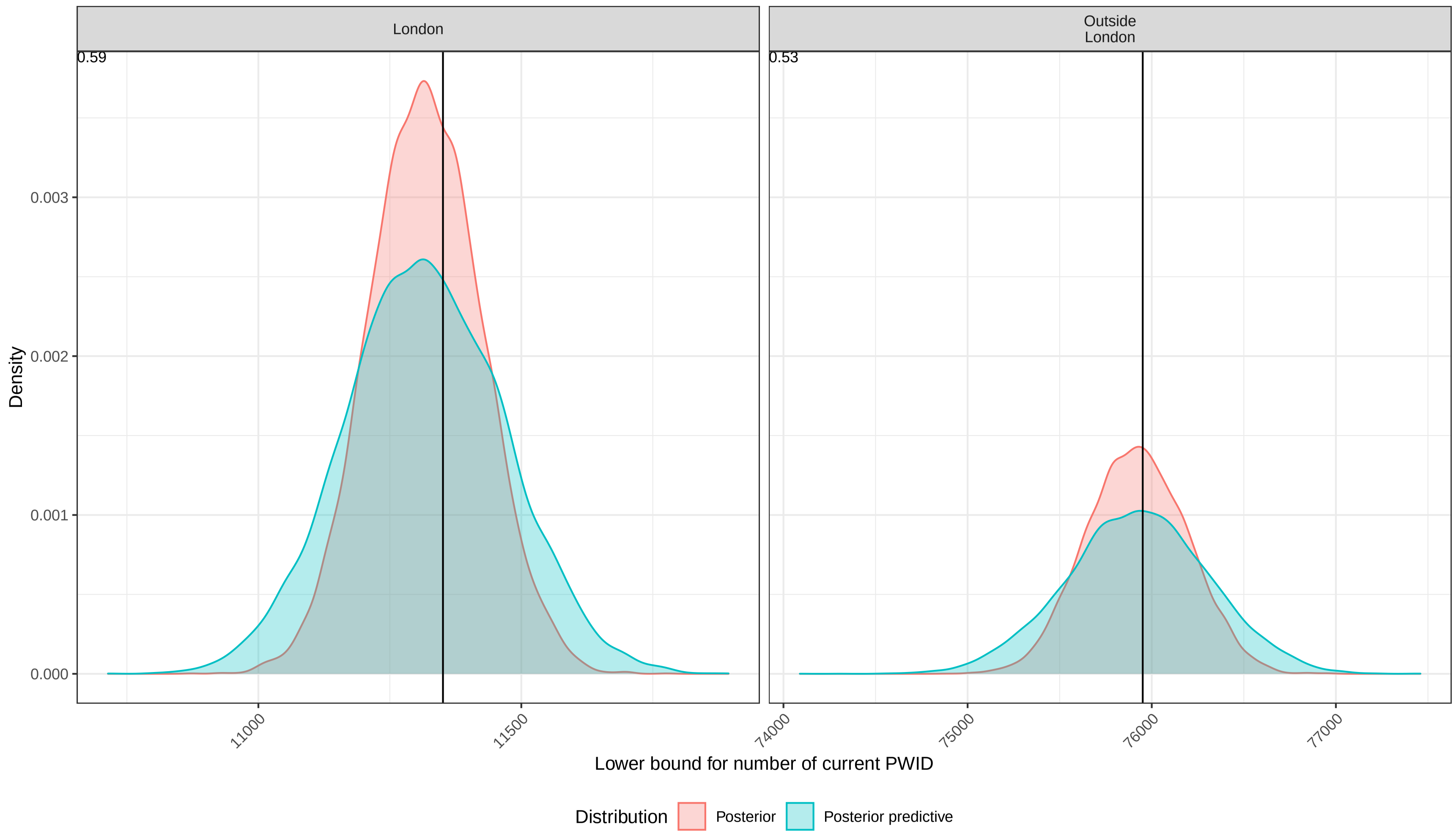

Figure 12: Posterior predictive checks of lower bound for absolute group size of current PWID. The vertical black lines are the observed data from the Home Office capture-recapture study[11]. The posterior predictive p-value is shown in the top left corner of each panel.

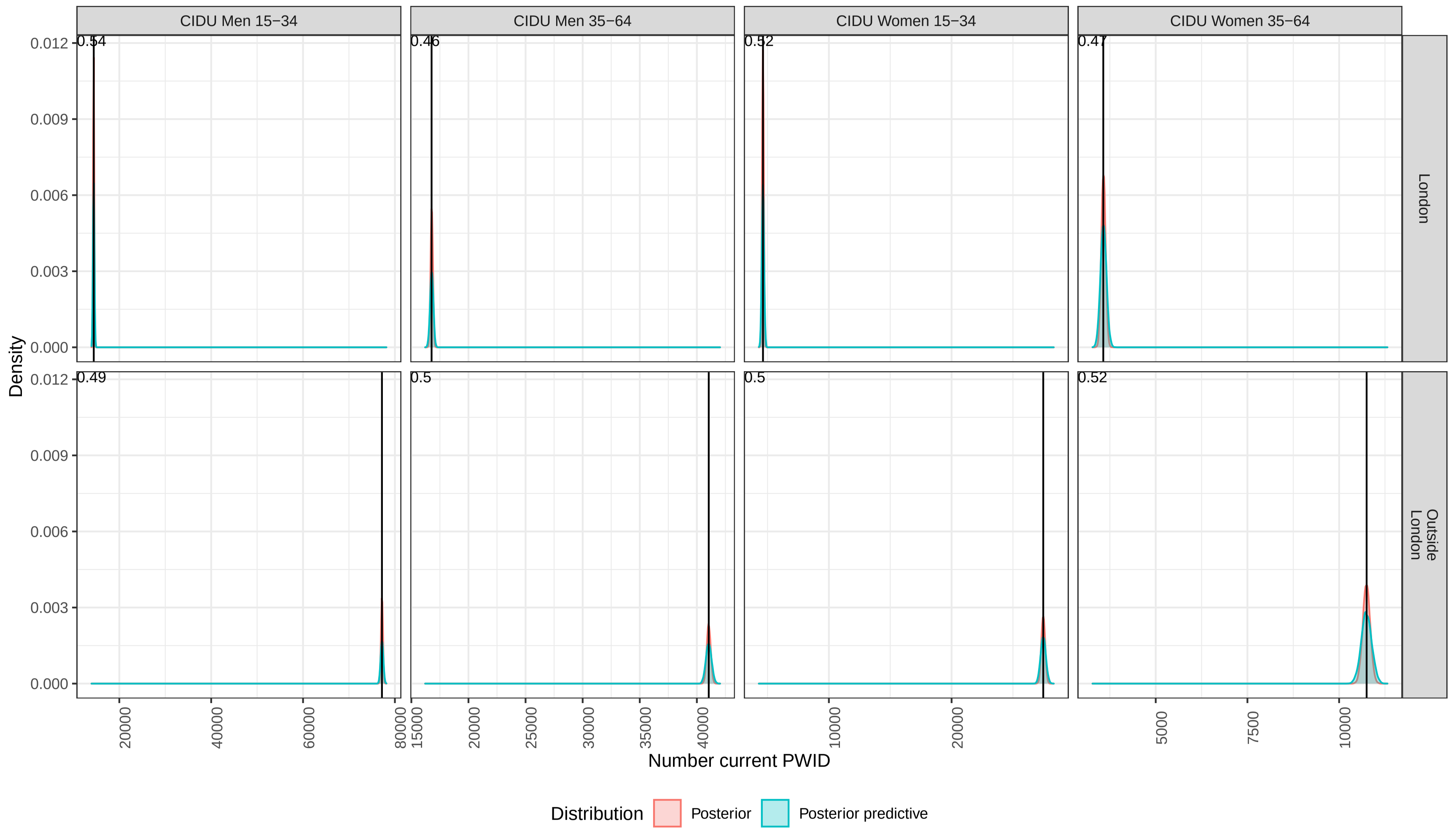

Figure 13: Posterior predictive checks of lower bound for age/gender distribution of PWID. The vertical black lines are the observed data from the King et al (2014)[12] study. The posterior predictive p-value is shown in the top left corner of each panel.

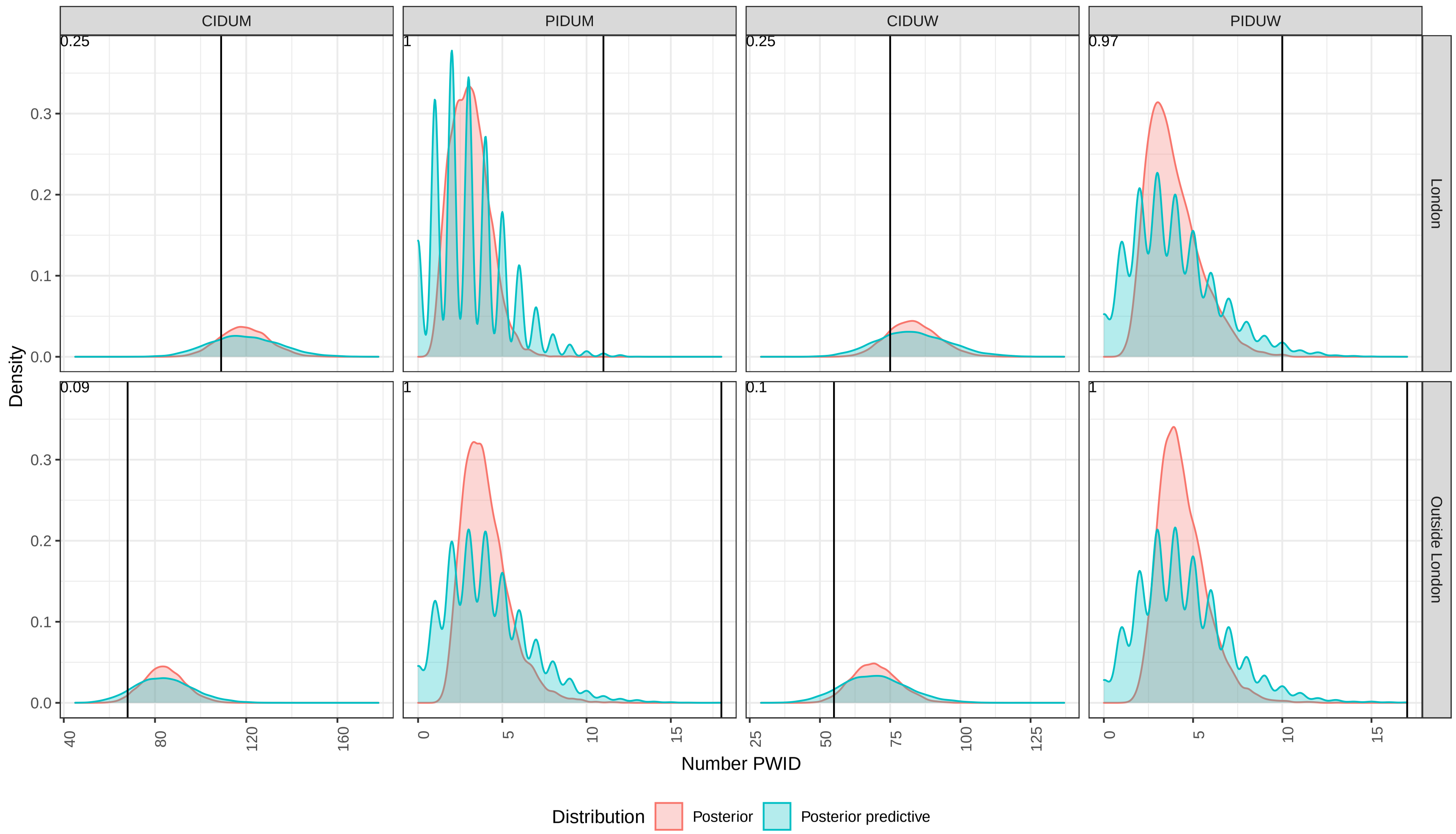

Figure 14: Posterior predictive checks of current/ex distribution of PWID. The vertical black lines are the observed data from the Sweeting et al (2009)[13] study. The posterior predictive p-value is shown in the top left corner of each panel.

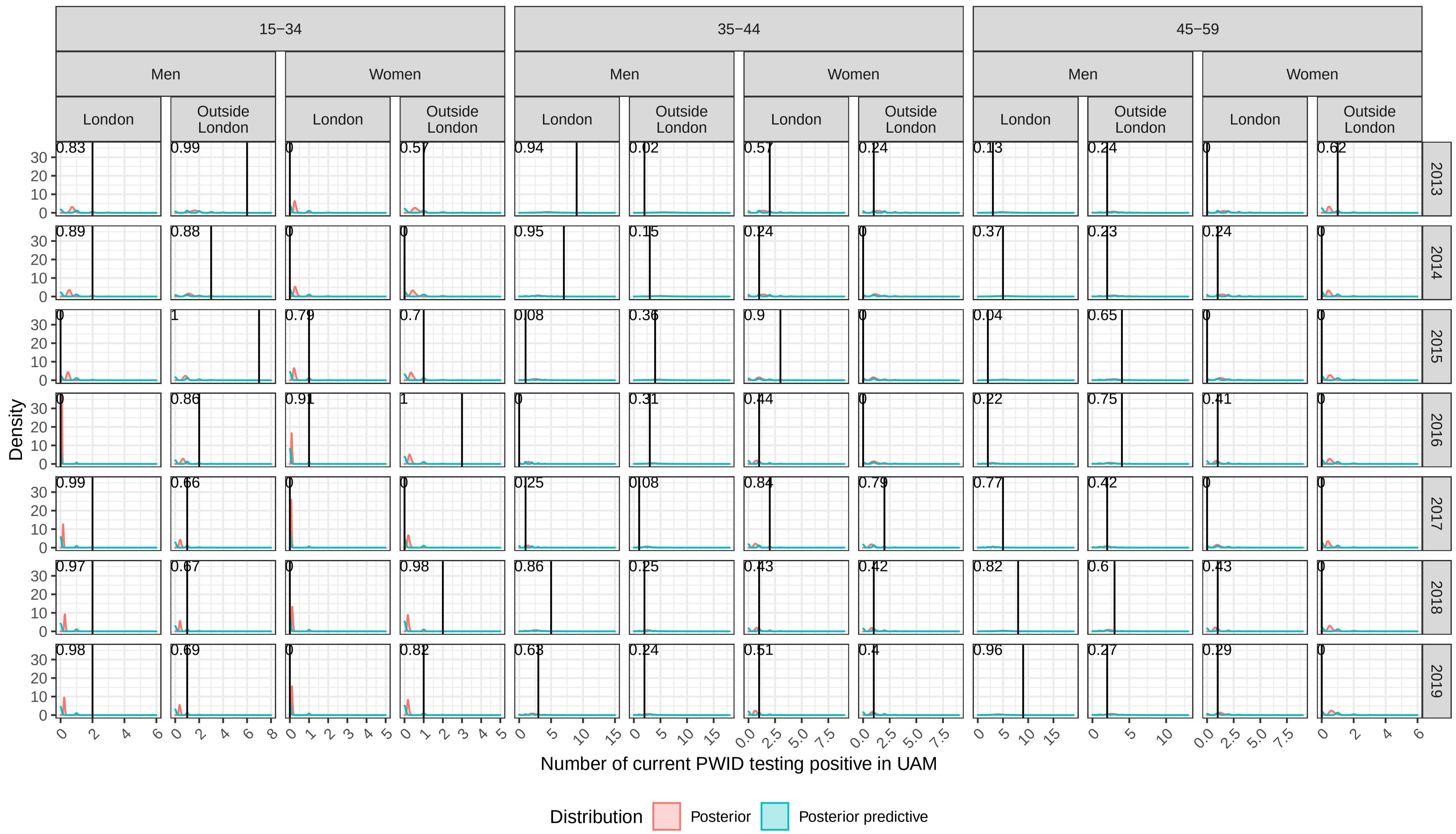

Figure 15: Posterior predictive checks of number of current PWID testing HIV positive in the UAM. The vertical black lines are the observed data from the UAM. The posterior predictive p-value is shown in the top left corner of each panel.

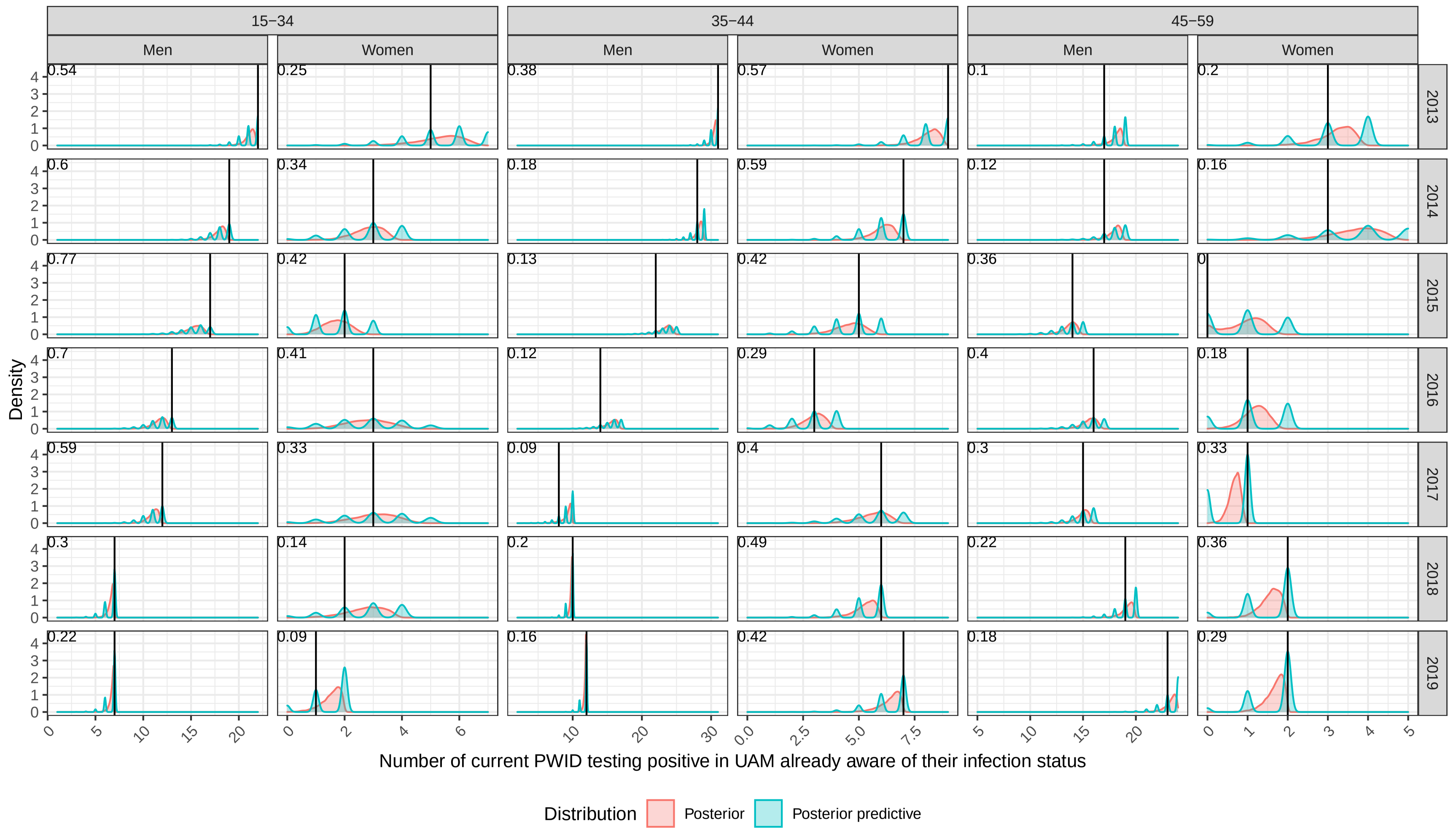

Figure 16: Posterior predictive checks of number of current PWID testing HIV positive in the UAM who were already aware of their infection status. The vertical black lines are the observed data from the UAM. The posterior predictive p-value is shown in the top left corner of each panel.

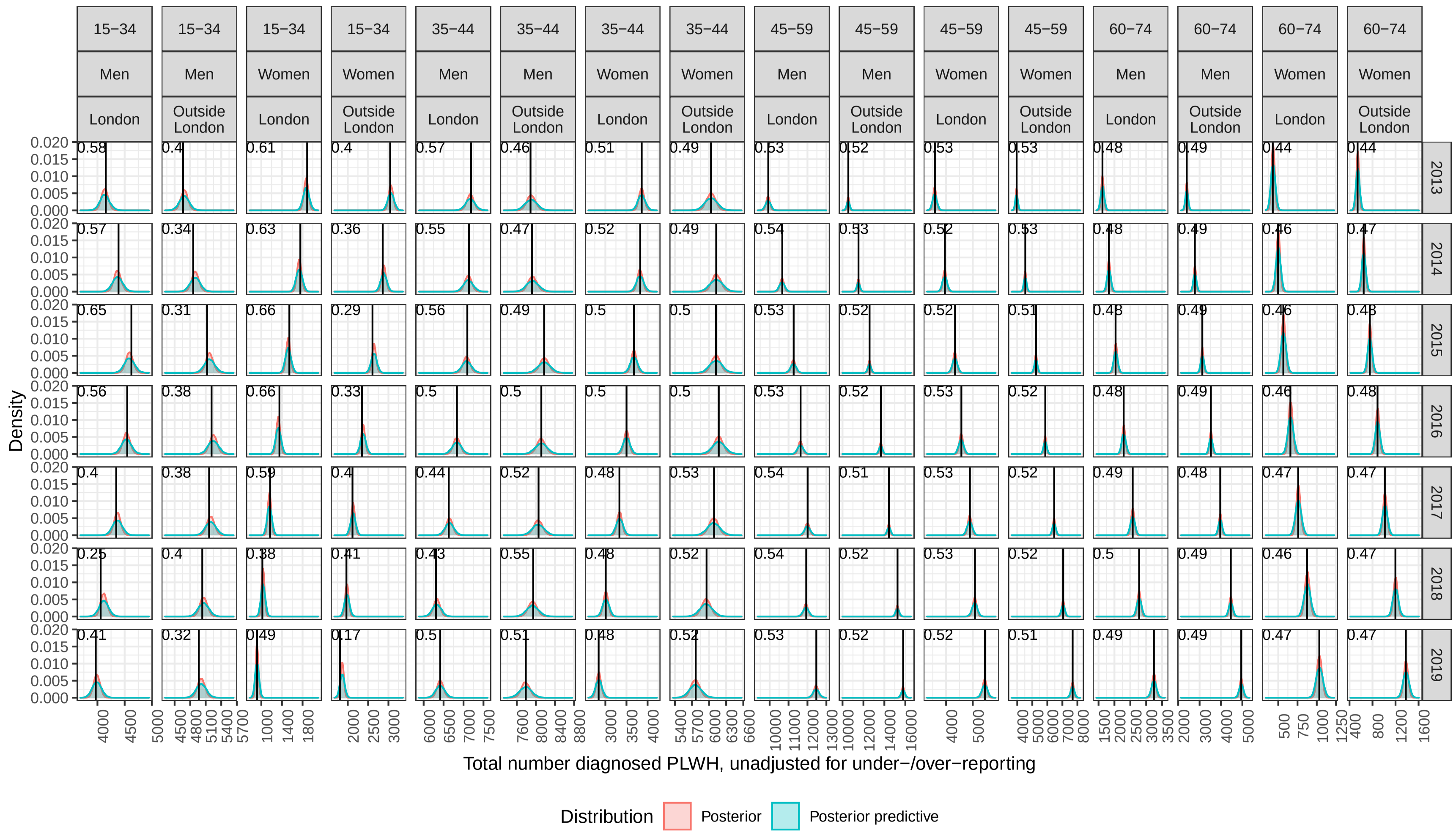

Figure 17: Posterior predictive checks of total number of people living with diagnosed HIV. The vertical black lines are the observed data from the HARS system. The posterior predictive p-value is shown in the top left corner of each panel.

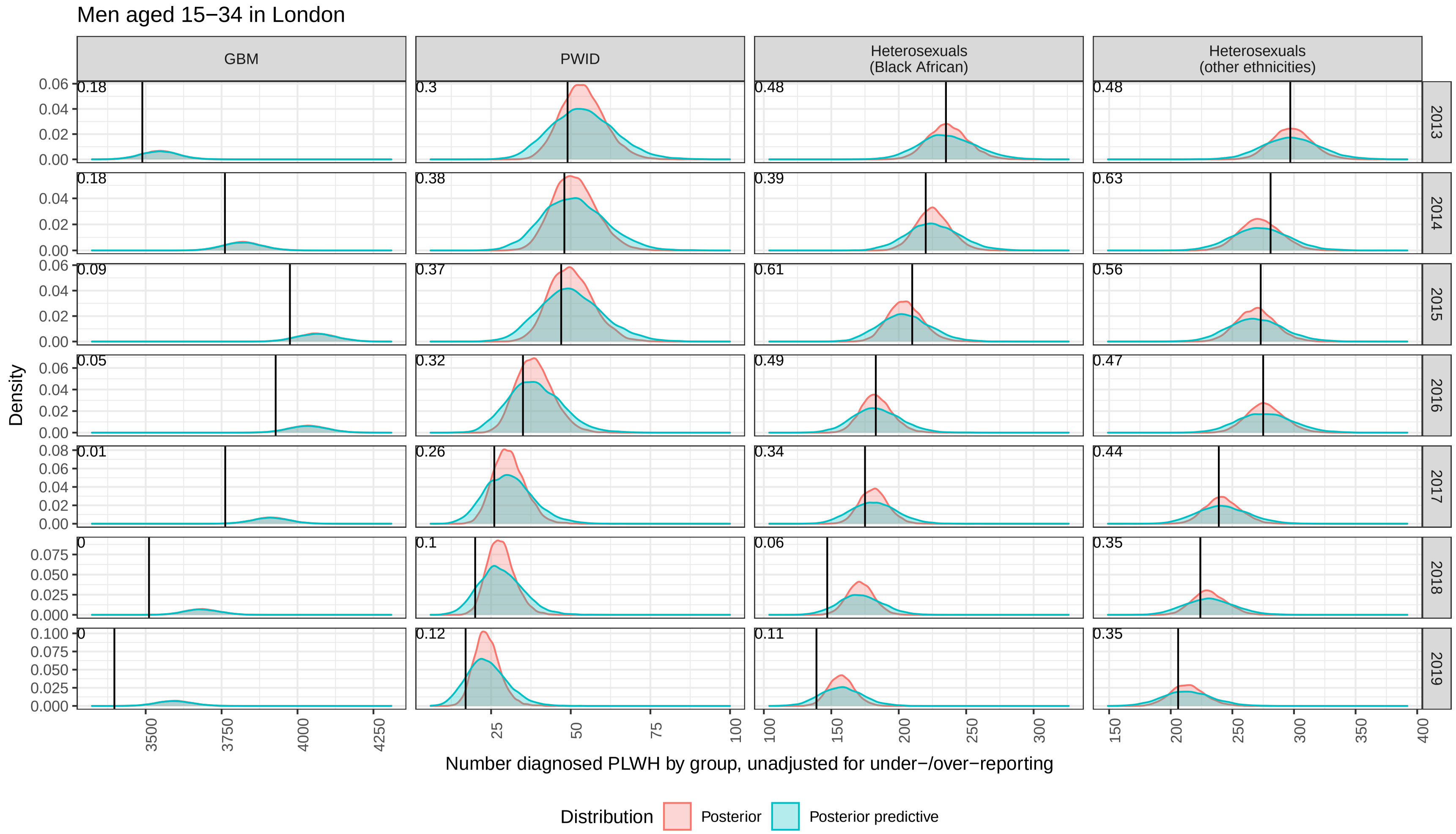

Figure 18: Posterior predictive checks of distribution across exposure groups (GBM, PWID, heterosexuals by ethnicity) of men aged 15-34 in London living with diagnosed HIV. The vertical black lines are the observed data from the HARS system. The posterior predictive p-value is shown in the top left corner of each panel.

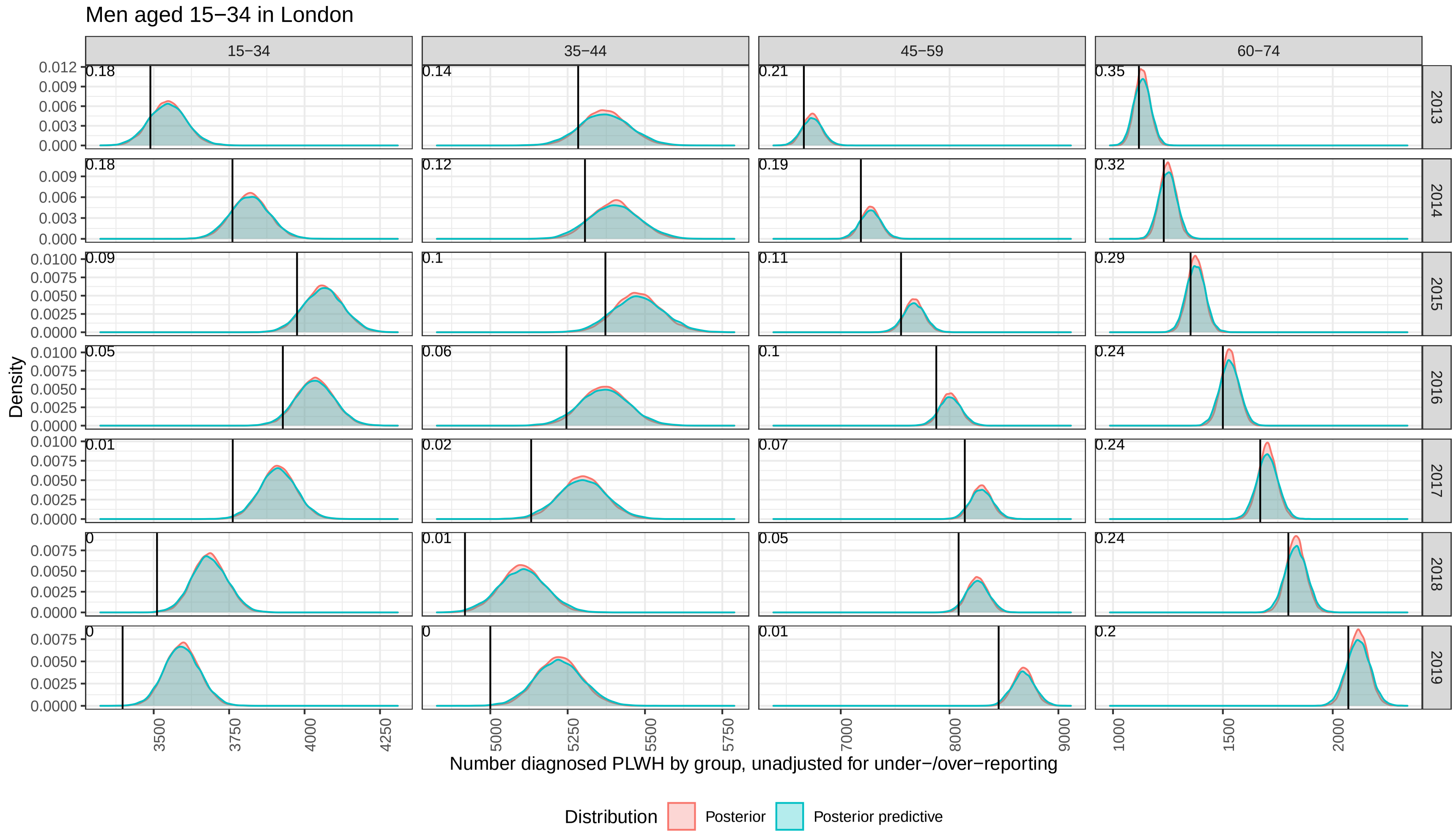

Figure 19: Posterior predictive checks of number of GBM by age group living with diagnosed HIV. The vertical black lines are the observed data from the HARS system. The posterior predictive p-value is shown in the top left corner of each panel.

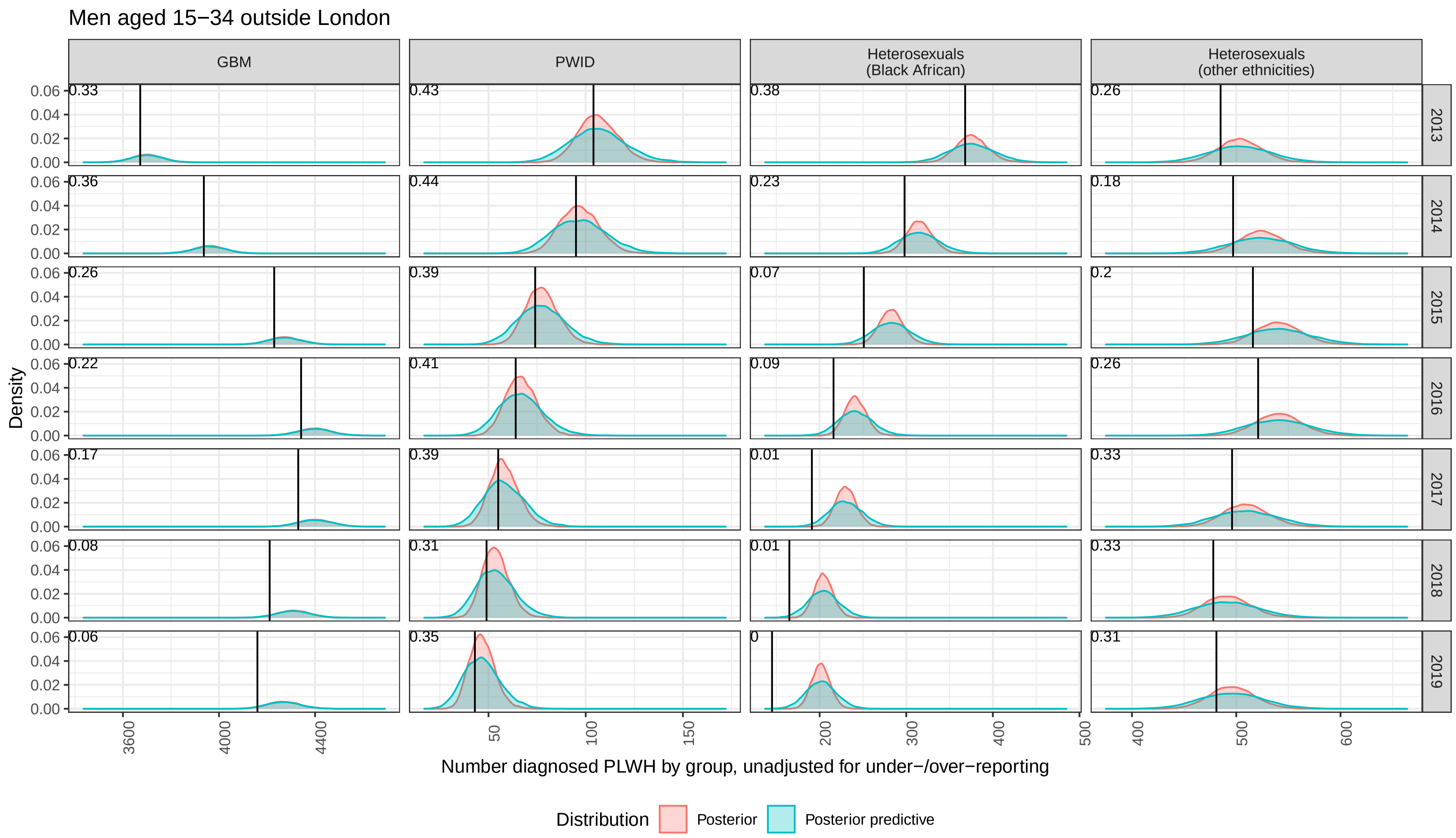

Figure 20: Posterior predictive checks of distribution across exposure groups (GBM, PWID, heterosexuals by ethnicity) of men aged 15-34 Outside London living with diagnosed HIV. The vertical black lines are the observed data from the HARS system. The posterior predictive p-value is shown in the top left corner of each panel.

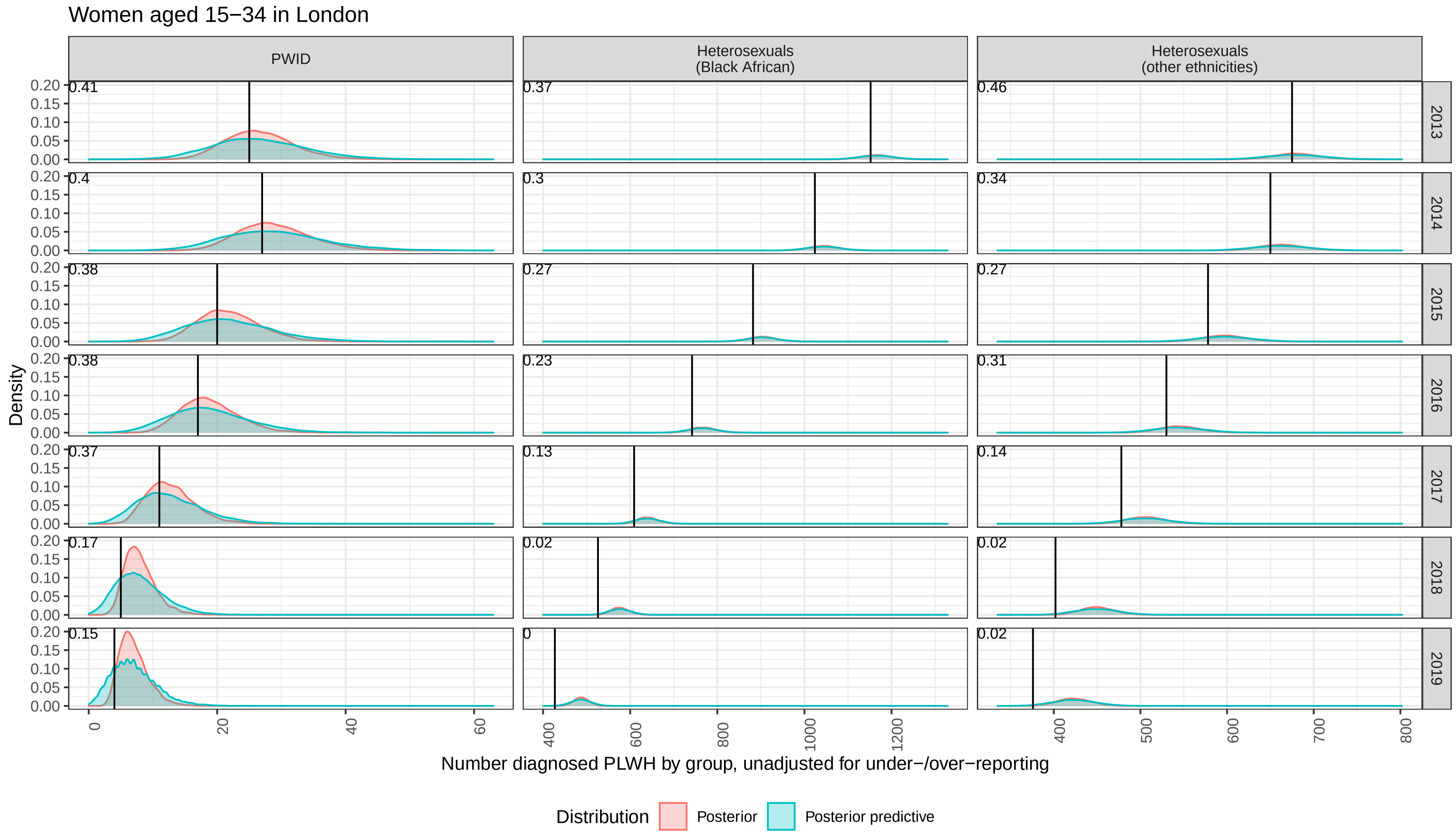

Figure 21: Posterior predictive checks of distribution across exposure groups (PWID, heterosexuals by ethnicity) of women aged 15-34 in London living with diagnosed HIV. The vertical black lines are the observed data from the HARS system. The posterior predictive p-value is shown in the top left corner of each panel.

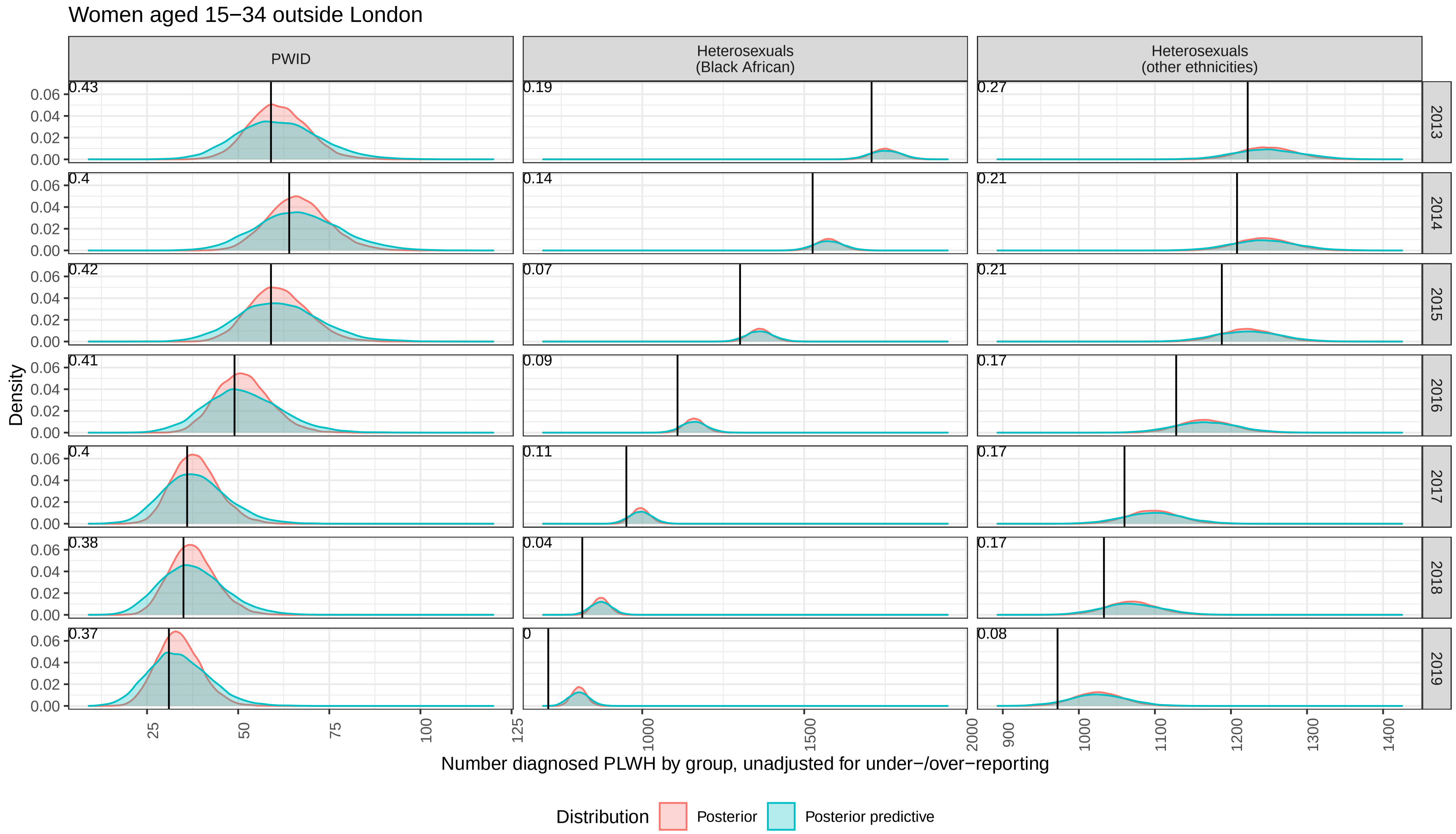

Figure 22: Posterior predictive checks of distribution across exposure groups (PWID, heterosexuals by ethnicity) of women aged 15-34 Outside London living with diagnosed HIV. The vertical black lines are the observed data from the HARS system. The posterior predictive p-value is shown in the top left corner of each panel.

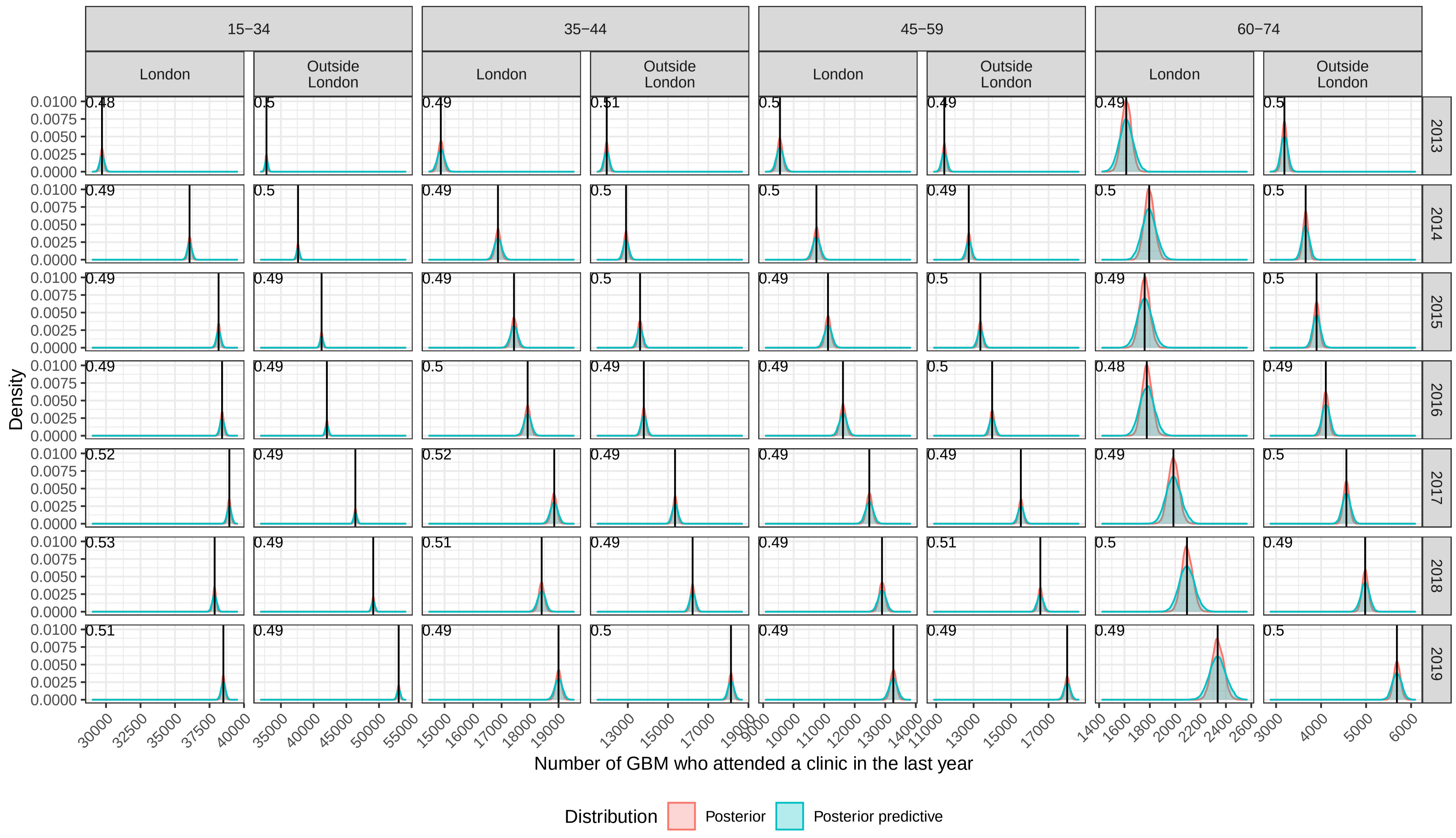

Figure 23: Posterior predictive checks of number of GBM who have attended a sexual health clinic in the last year. The vertical black lines are the observed data from GUMCAD. The posterior predictive p-value is shown in the top left corner of each panel.

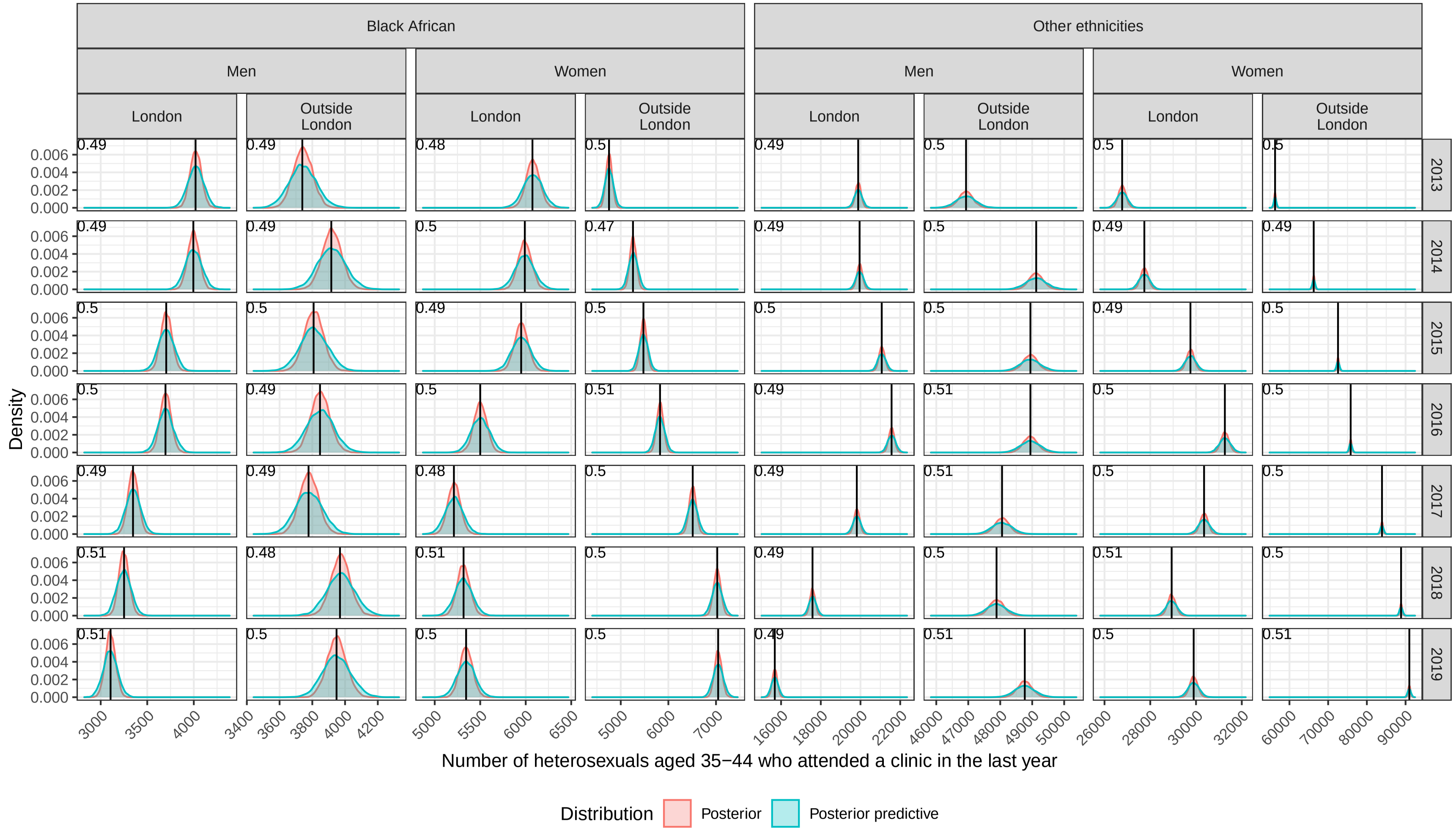

Figure 24: Posterior predictive checks of number of heterosexuals aged 35-44 who have attended a sexual health clinic in the last year, by ethnicity. The vertical black lines are the observed data from GUMCAD. The posterior predictive p-value is shown in the top left corner of each panel.

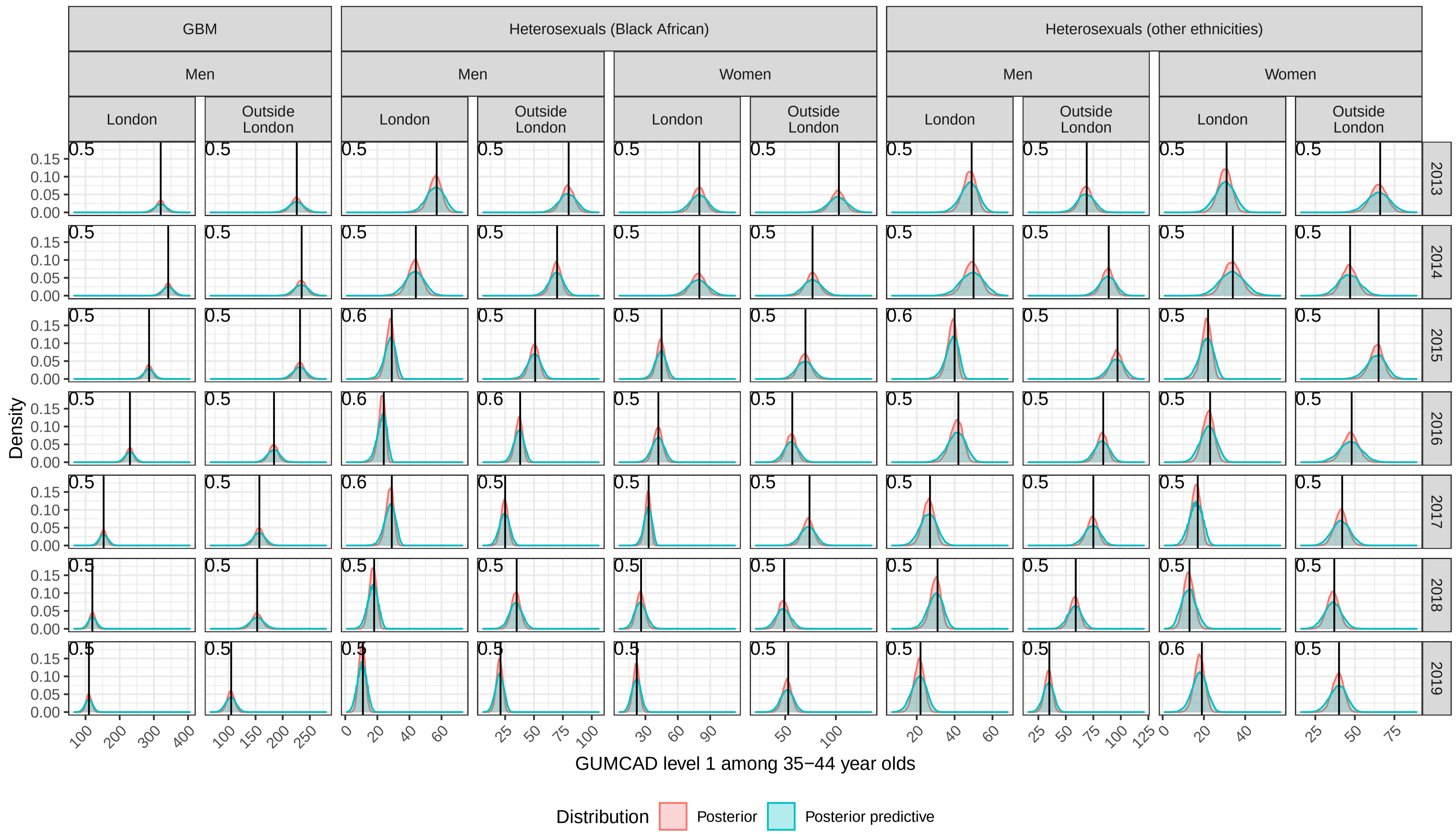

Figure 25: Posterior predictive checks of lower bound for the number of individuals aged 35-44 who are newly diagnosed with HIV in a sexual health clinic in the last year. The vertical black lines are the observed data from GUMCAD. The posterior predictive p-value is shown in the top left corner of each panel.

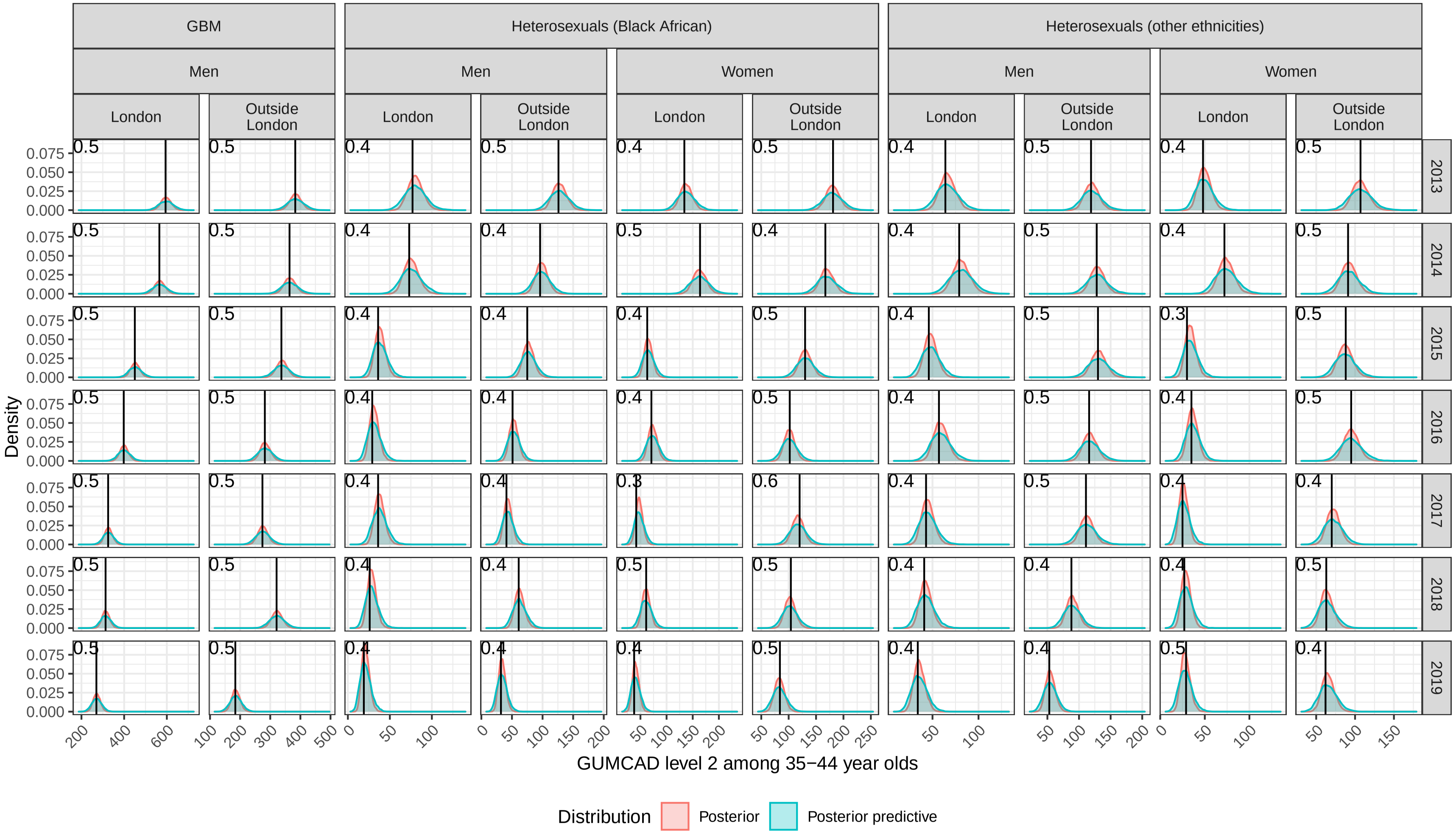

Figure 26: Posterior predictive checks of upper bound for the number of individuals aged 35-44 who are newly diagnosed with HIV in a sexual health clinic in the last year. The vertical black lines are the observed data from GUMCAD. The posterior predictive p-value is shown in the top left corner of each panel.

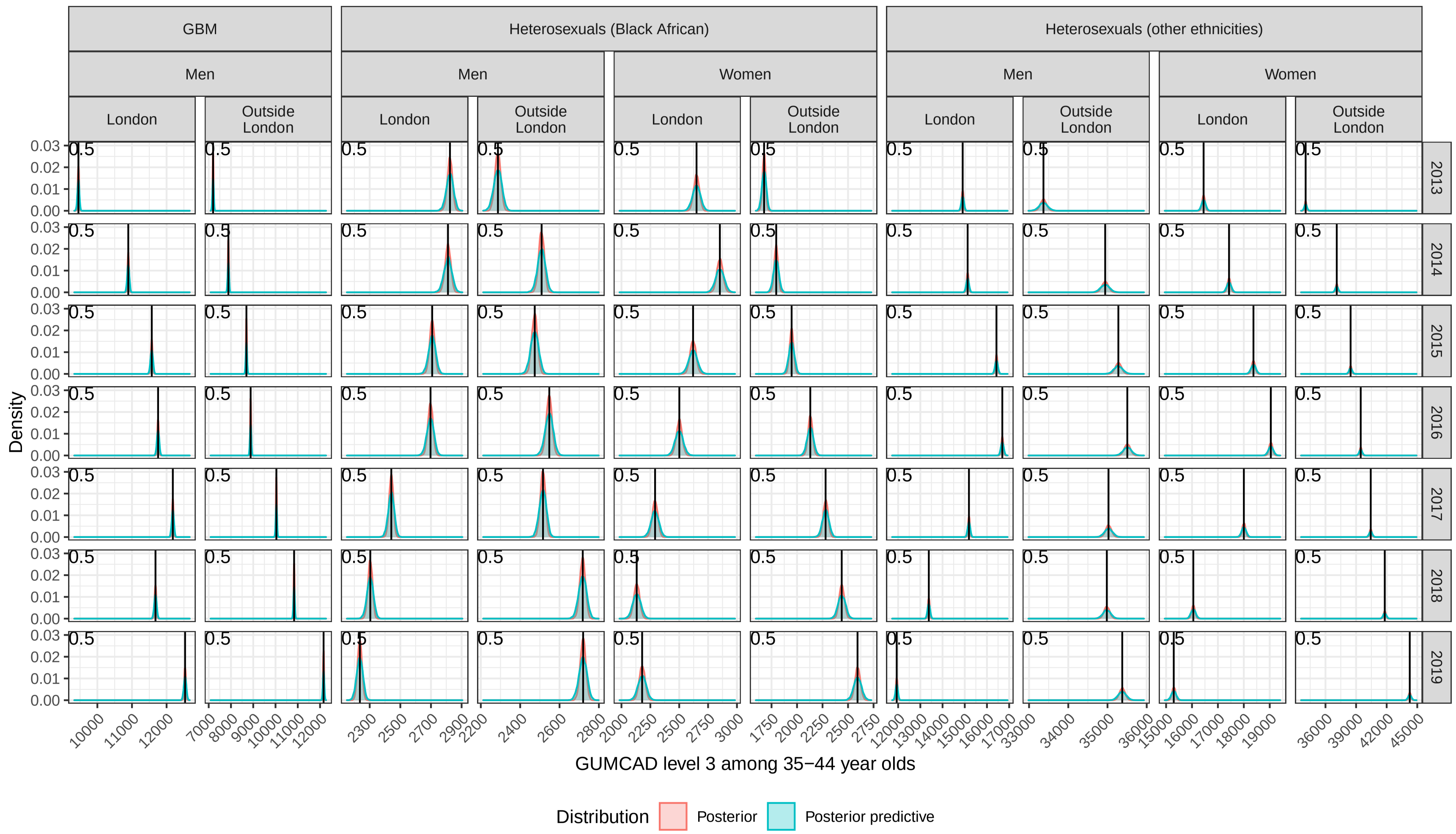

Figure 27: Posterior predictive checks of the number of individuals aged 35-44 who accept a HIV test in a sexual health clinic in the last year. The vertical black lines are the observed data from GUMCAD. The posterior predictive p-value is shown in the top left corner of each panel.

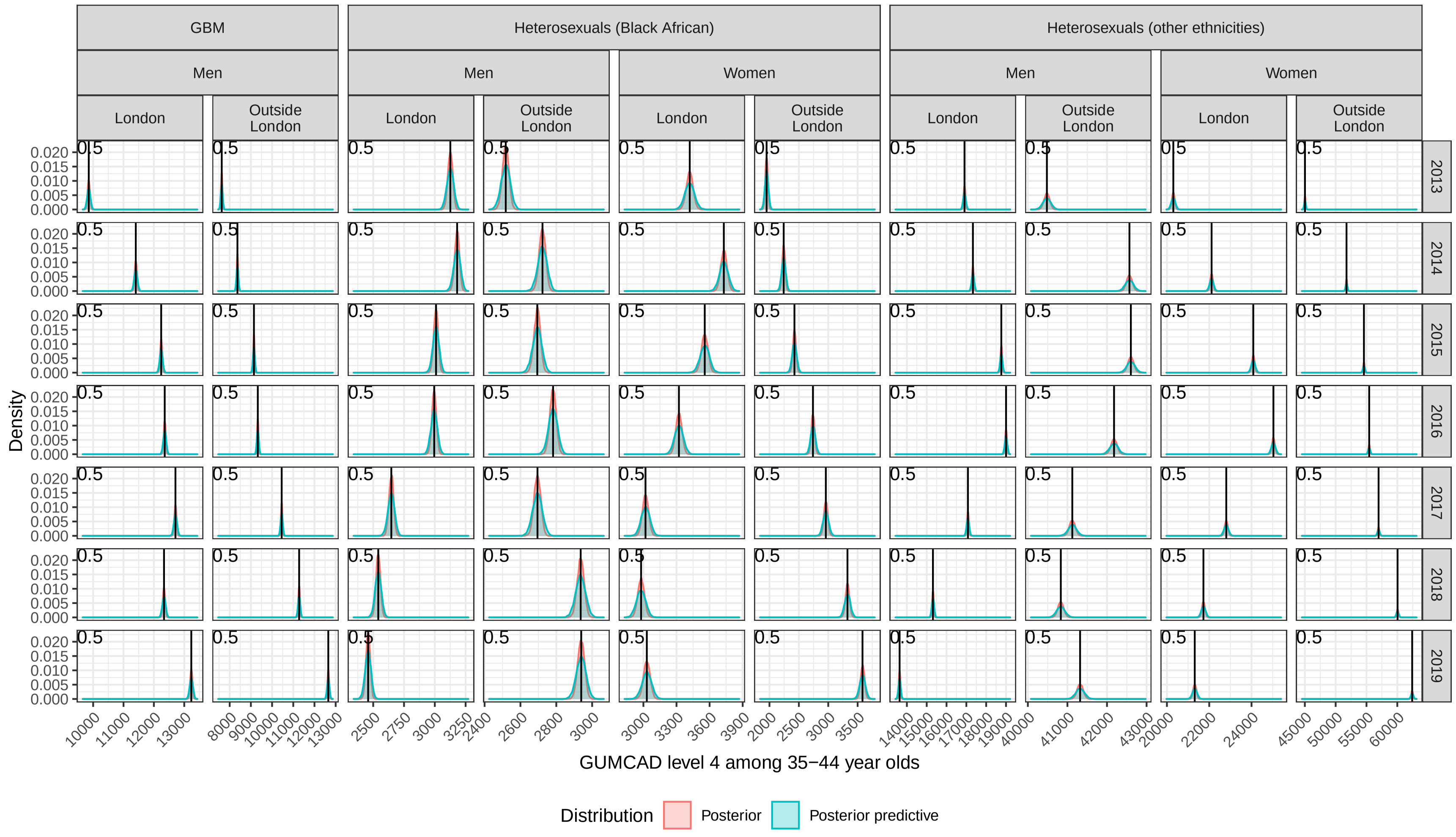

Figure 28: Posterior predictive checks of the number of individuals aged 35-44 who are offered a HIV test in a sexual health clinic in the last year. The vertical black lines are the observed data from GUMCAD. The posterior predictive p-value is shown in the top left corner of each panel.

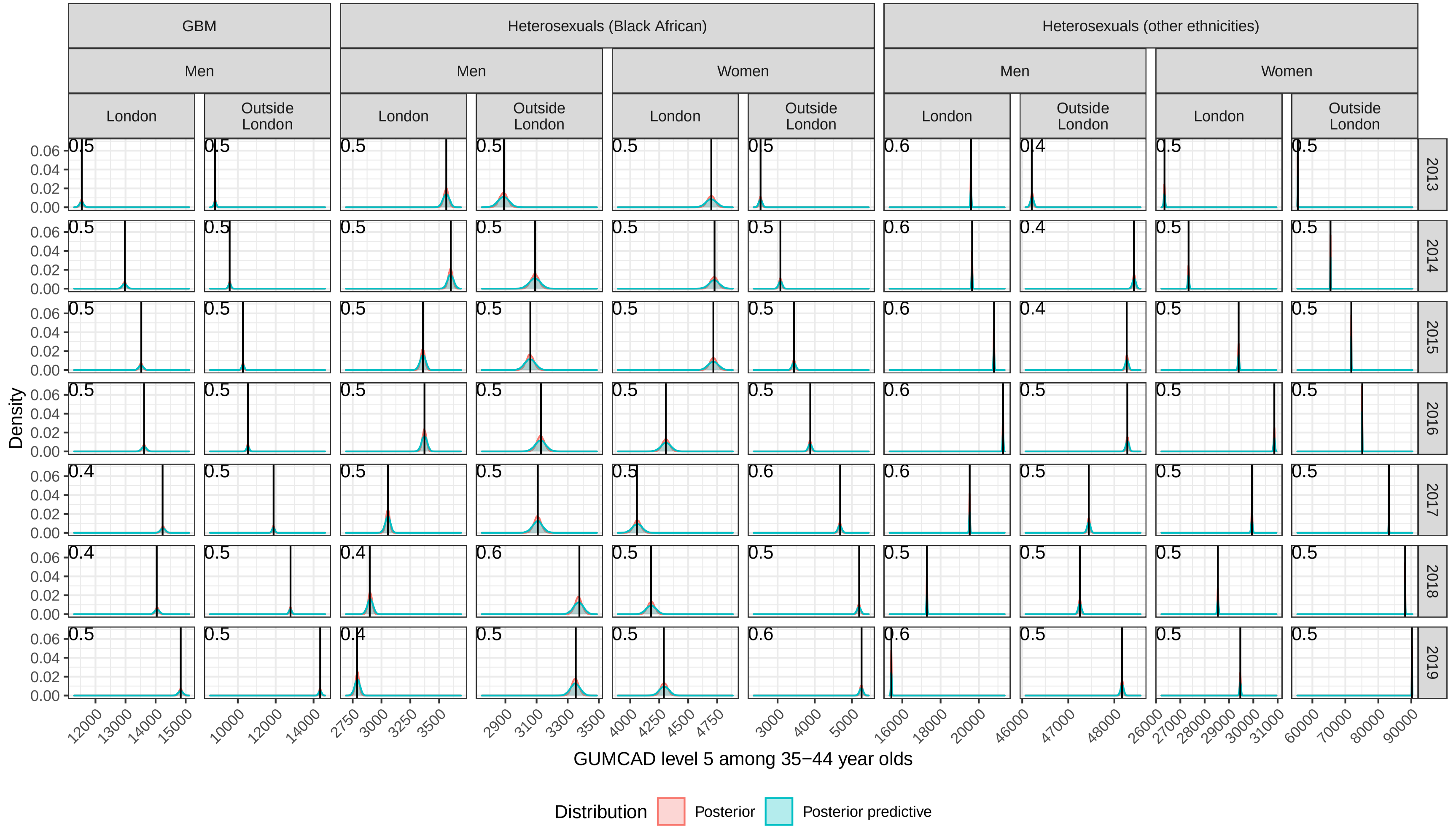

Figure 29: Posterior predictive checks of the number of individuals aged 35-44 who are eligible for a HIV test in a sexual health clinic in the last year. The vertical black lines are the observed data from GUMCAD. The posterior predictive p-value is shown in the top left corner of each panel.

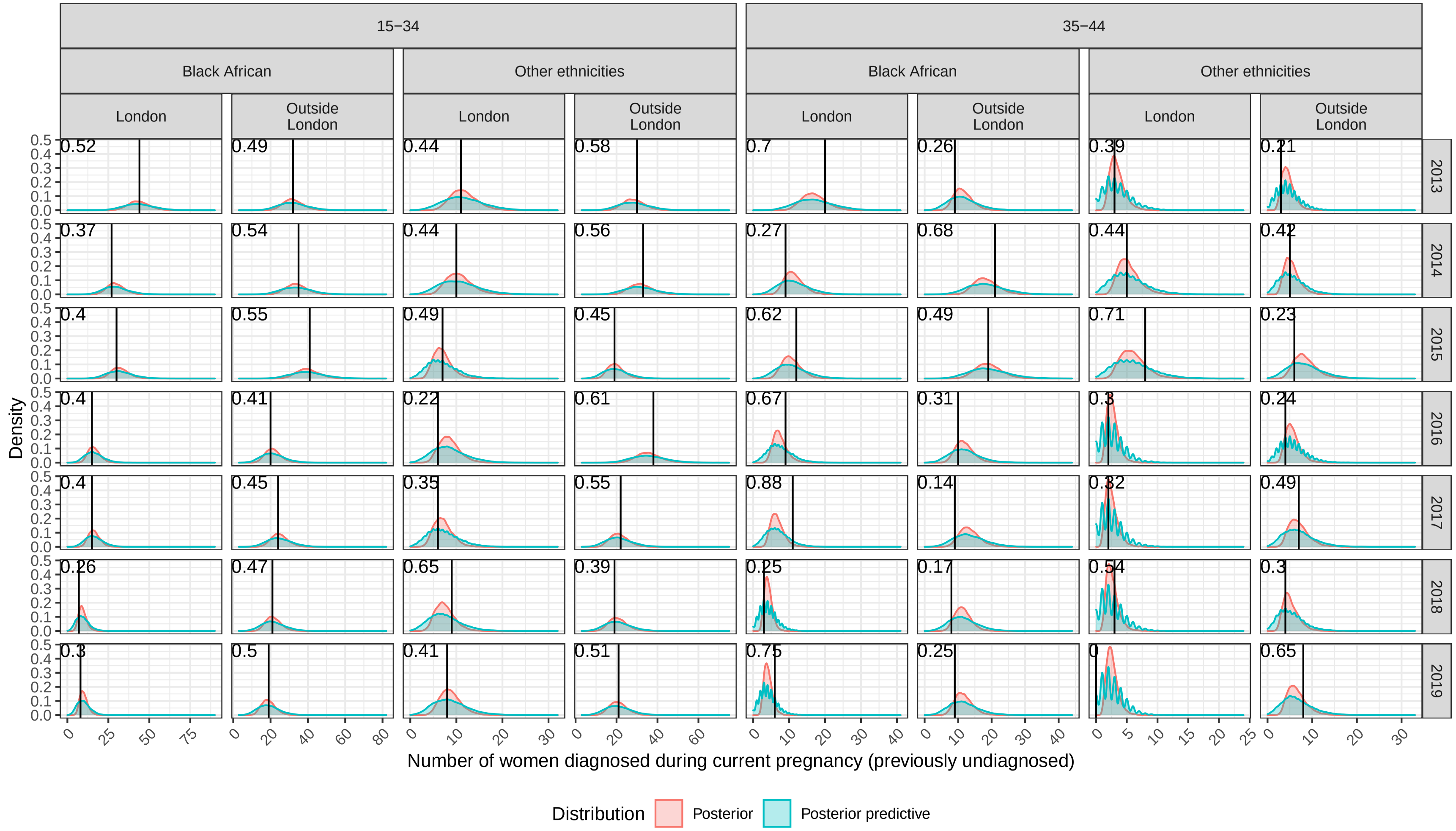

Figure 30: Posterior predictive checks of the number of women diagnosed with HIV during her current pregnancy (i.e. previously undiagnosed). The vertical black lines are the observed data from the NSHPC. The posterior predictive p-value is shown in the top left corner of each panel.

References

[1] Goubar A, Ades AE, De Angelis D, McGarrigle CA, Mercer CH, Tookey PA, et al. Estimates of human immunodeficiency virus prevalence and proportion diagnosed based on Bayesian multiparameter synthesis of surveillance data. Journal of the Royal Statistical Society Series A: Statistics in Society. 2008;171(3):541–580.

[2] Presanis AM, Gill ON, Chadborn TR, Hill C, Hope V, Logan L, et al. Insights into the rise in HIV infections, 2001 to 2008: a Bayesian synthesis of prevalence evidence. AIDS. 2010;24(18):2849–2858.

[3] De Angelis D, Presanis AM, Conti S, Ades AE. Estimation of HIV burden through Bayesian evidence synthesis. Statistical Science. 2014;29(1):9–17.

[4] Carpenter B, Gelman A, Hoffman MD, Lee D, Goodrich B, Betancourt M, et al. Stan: A Probabilistic Programming Language. Journal of Statistical Software. 2017;76(1). Available from: http://www.jstatsoft.org/v76/i01/.

[5] Spiegelhalter DJ, Best NG, Carlin BP, Van Der Linde A. Bayesian measures of model complexity and fit. Journal of the Royal Statistical Society: Series B (Statistical Methodology). 2002;64(4):583–639.

[6] R Core Team. R: A Language and Environment for Statistical Computing. Vienna, Austria; 2018. Available from: https://www.R-project.org/.

[7] Stan Development Team. RStan: the R interface to Stan; 2018. R package version 2.17.3. Available from: http://mc-stan.org/.

[8] Mercer CH, Prah P, Field N, Tanton C, Macdowall W, Clifton S, et al. The health and well-being of men who have sex with men (MSM) in Britain : Evidence from the third National Survey of Sexual Attitudes and Lifestyles (Natsal-3). BMC Public Health. 2016:1–16. Available from: http://dx.doi.org/10.1186/s12889-016-3149-z.

[9] Savage EJ, Mohammed H, Leong G, Duffell S, Hughes G. Improving surveillance of sexually transmitted infections using mandatory electronic clinical reporting: The genitourinary medicine clinic activity dataset, England, 2009 to 2013. Eurosurveillance. 2014;19(48):1–9.

[10] Aghaizu A, Wayal S, Nardone A, Parson V, Copas AJ, Mercey DE, et al. Understanding continuing high HIV incidence: trends in sexual behaviours, HIV testing and the proportion of men at risk of transmitting and acquiring HIV in London 2000-2013. A serial cross-sectional study. The Lancet HIV. 2016;3(9):e431–e440.

[11] Hay G, Gannon M, Casey J, Millar T. Estimates of the Prevalence of Opiate Use and/or Crack Cocaine Use, 2009/10: Sweep 6 report. Centre for Public Health, Liverpool John Moores University; 2011. 6. Available from: http://citeseerx.ist.psu.edu/viewdoc/download?doi=10.1.1.690.2170&rep=rep1&type=pdf.

[12] King R, Bird SM, Hay G, Hutchingson SJ. Estimating Prevalence of Injecting Drug Users and Associated Death Rates in England Using Regional Data and Incorporating Prior Information. Journal of the Royal Statistical Society: Series A (Statistics in Society). 2014;177(1):209–236. Available from: https://rss.onlinelibrary.wiley.com/doi/pdf/10.1111/rssa.12011.

[13] Sweeting MJ, De Angelis D, Ades AE, Hickman M. Estimating the prevalence of ex-injecting drug use in the population. Statistical Methods in Medical Research. 2009;18(4):381–395. Available from: http://dx.doi.org/10.1177/0962280208094704.

[14] Public Health England. Shooting Up: Infections among people who inject drugs in the UK, 2017. Public Health England; 2018. Available from: https://assets.publishing.service.gov.uk/government/uploads/system/uploads/attachment_data/file/756502/Shooting_up_2018.pdf, accessed 29th April 2019.

[15] Office for National Statistics. Ethnicity and National Identity in England and Wales: Census 2011. Office for National Statistics; 2012. Available from: https://www.ons.gov.uk/peoplepopulationandcommunity/culturalidentity/ethnicity/articles/ethnicityandnationalidentityinenglandar 12-11, accessed 29th April 2019.

[16] Peters H, Thorne C, Tookey PA, Byrne L. National audit of perinatal HIV infections in the UK, 2006–2013: what lessons can be learnt? HIV Medicine. 2018;19(4):280–289.

[17] Office for National Statistics. Births in England and Wales by Parents' Country of Birth. Office for National Statistics; 2018. Available from: https://www.ons.gov.uk/peoplepopulationandcommunity/birthsdeathsandmarriages/livebirths/bulletins/parentscountryofbirthengla accessed 29th April 2019.

[18] Office for National Statistics. Detailed country of birth and nationality analysis from the 2011 Census of England and Wales; 2013. Available from: https://www.ons.gov.uk/peoplepopulationandcommunity/populationandmigration/populationestimates/articles/detailedcountryofbi 05-13/pdf, accessed 29th April 2019.

[19] Coleman D, Dubuc S. The fertility of ethnic minorities in the UK, 1960s-2006. Population studies. 2010;64(1):19–41.

[20] Bourne A, Reid D, Weatherburn P. African Health and Sex Survey 2013-2014: headline findings. London, England: Sigma Research, London School of Hygiene and Tropical Medicine; 2014. Available from: http://www.sigmaresearch.org.uk/files/report2014c.pdf, accessed 29th April 2019.

[21] National Health Service Blood and Transplant, Public Health England. Safe supplies 2017: data sources and methods. NHS Blood and Transplant, Public Health England; 2017. Available from: https://www.gov.uk/government/publications/safe-supplies-annual-review, accessed 29th April 2019.

[22] Davison, K L and Reynolds, C A and Andrews, N and Brailsford, S R and & on behalf of the UK Blood Donor Survey Steering. Getting personal with blood donors - the rationale for, methodology of and an overview of participants in the UK blood donor survey. Transfusion Medicine. 2015;25(4):265–275.
